# Supplementary material for: Closing the Empirical Loop: Autonomous AI Agents Conduct End‐to‐end Research With Human Participants
Source: Adv Sci (Weinh). 2026 Sep 14;13(56):e76675. doi: 10.1002/advs.76675 (PMC13573717; doi:10.1002/advs.76675)
Supplement: Supplementary file 1 [file ADVS-13-e76675-s001.pdf]

Closing the empirical loop: autonomous AI agents conduct end-to-end research with human participants

Supplementary Material

Table of Contents

1. Study 1 Autonomously Generated Manuscript - *Independence of visual working memory precision and mental rotation performance: theoretical and methodological implications*

2. Study 2 Autonomously Generated Manuscript - *Imagery vividness fails to predict serial dependence in visual working memory and mental rotation*

3. Study 3 Autonomously Generated Manuscript - *Visual memory precision shows negligible spatial task links*

Independence of visual working memory precision and mental rotation performance: theoretical and methodological implications

Explore Science
research@explorescience.ai

July 22, 2025

AUTONOMOUSLY GENERATED

Abstract

Resource theories of visual cognition propose that visual working memory and mental rotation rely on shared representational constraints, with individual differences in imagery vividness potentially moderating these relationships. However, systematic investigation of performance patterns across these domains remains limited. We tested whether visual working memory precision and mental rotation performance exhibit correlated decline patterns under increasing cognitive demand, and whether imagery vividness moderates these relationships. In a large-scale online study, 181 participants completed visual working memory tasks (varying set size and delay duration), mental rotation tasks (across rotation angles), and imagery vividness questionnaires. We calculated individual slope parameters quantifying how performance declined with increasing cognitive demand in each task. Contrary to resource theory predictions, visual working memory and mental rotation performance patterns showed no significant correlations, with negligible effect sizes. Imagery vividness did not moderate performance in either task across any experimental manipulation. Split-half reliability analyses revealed that three of four slope parameters showed poor internal consistency, providing a methodological explanation for the null correlational findings. These results fail to support theories proposing shared representational constraints between visual working memory and mental rotation processes. The findings highlight critical measurement challenges in individual differences research and suggest that these cognitive domains may operate through more independent mechanisms than previously theorized. Future research should prioritize developing reliable individual difference measures before drawing conclusions about relationships between visual cognitive processes.

Keywords: visual working memory, mental rotation, individual differences, reliability paradox, cognitive architecture

1 Introduction

The human capacity to maintain and manipulate visual information, as first systematically investigated by Miller (1956), represents one of the most fundamental constraints on cognitive performance, yet the mechanisms underlying these limitations remain actively debated. Understanding how the brain allocates limited representational resources across different visual cognitive demands has profound implications for theories of mental architecture, individual differences in cognitive ability, and the flexibility of human information processing. As pioneered by Baddeley and Hitch (1984), working memory involves multiple components that support both maintenance and manipulation of information, yet a central tension exists between modular accounts that treat visual working memory maintenance and spatial transformation as independent systems - a view consistent with the modularity thesis (Fodor, 1985) - and resource-sharing frameworks that propose common constraints govern performance across these domains. Since the seminal work of Shepard and Metzler (1971) on three-dimensional rotation, spatial transformation abilities have been extensively studied, but their relationship to visual working memory processes remains theoretically contentious. The present investigation addresses this theoretical divide through large-scale online experimentation with sophisticated reliability assessment, examining whether precision patterns in visual working memory and mental rotation tasks reflect shared representational constraints.

Contemporary theories of visual working memory have converged on resource-based models that fundamentally challenge traditional capacity limitations, building upon earlier capacity theories (Just and Carpenter, 1992) that anticipated how increasing task demands reduce precision. According to the influential framework developed by Ma et al. (2014) and Bays and Husain (2008), visual working memory operates as a continuous resource system where representational precision varies systematically with the allocation of limited cognitive resources, which diverges from the classic slot model (Luck and Vogel, 1997). This resource theory generates specific testable predictions about shared representational constraints: if visual working memory and mental rotation processes draw upon common representational resources, then individuals who show greater sensitivity to increased cognitive demands in one domain should exhibit corresponding sensitivity in the other domain. Specifically, this translates to predicted positive correlations between slope parameters that quantify individual differences in performance decline patterns - the rate at which visual working memory precision deteriorates with increasing memory load should correlate with the rate at which mental rotation accuracy declines with angular disparity, and the rate of precision loss over longer retention intervals should correlate with the rate of reaction time increases during spatial transformation. These slope parameters represent theoretically motivated individual difference measures because they capture the efficiency of resource allocation under increasing cognitive demands, where neural correlates of storage limitations manifest (Vogel et al., 2005). Neural evidence supports this framework, with overlapping parietal activations observed during both maintenance and transformation of visual information (Christophel et al., 2017), suggesting that similar computational principles may constrain representational fidelity across different cognitive operations.

Despite the theoretical elegance of resource-sharing accounts, the empirical landscape reveals a more complex and inconsistent picture that challenges straightforward interpretations of shared cognitive constraints. Recent attempts to replicate foundational findings have exposed significant methodological vulnerabilities in this research domain, reflecting broader reproducibility concerns

AUTONOMOUSLY GENERATED

identified across psychological science (Collaboration, 2015). Ebert et al. (2024) failed to replicate the influential study by Hyun and Luck (2010), which had suggested that object working memory, but not spatial working memory, is employed during mental rotation, instead finding general interference effects rather than the predicted rotation-dependent interference patterns. This replication failure exemplifies a broader pattern of inconsistent findings that has emerged when rigorous experimental controls are applied to test specific predictions about shared representational systems. The discrepancies in the literature suggest that previous approaches may have been insufficient to detect the subtle individual differences that would constitute evidence for shared resource constraints, highlighting the need for methodological innovations that can address these empirical inconsistencies while maintaining theoretical rigor.

The challenges facing this field extend beyond isolated replication failures to encompass a fundamental measurement problem known as the reliability paradox (Hedge et al., 2017), which directly constrains the ability to test resource-sharing theories through correlational approaches. Tasks that produce robust experimental effects at the group level - precisely the characteristic that makes them popular research tools - often exhibit poor reliability for measuring individual differences due to low between-subject variability. This paradox severely constrains the ability to detect meaningful correlations between cognitive measures, as reliable individual differences require substantial variance between participants. The measurement of slope parameters that quantify sensitivity to cognitive demands presents particular challenges, as these derived measures must capture meaningful individual differences while maintaining adequate psychometric properties. Traditional approaches to studying individual differences in cognitive performance have largely overlooked these reliability constraints, potentially leading to both false positive and false negative conclusions about relationships between cognitive domains. The specific knowledge gap that remains unresolved concerns whether the absence of consistent cross-domain correlations in previous studies reflects genuine theoretical disconfirmation of resource-sharing accounts or methodological artifacts arising from poor measurement reliability. The present investigation addresses this critical gap through large-scale online experimentation, consistent with established approaches for behavioral research (Crump et al., 2013), combined with comprehensive reliability assessment that allows for direct evaluation of whether measurement precision is sufficient to detect theoretically predicted relationships. This methodological approach enables a definitive test of resource-sharing theories while providing transparent assessment of the measurement constraints that may have limited previous investigations.

AUTONOMOUSLY GENERATED

2 Method

2.1 Preregistration and Open Science Practices

This study was preregistered prior to data collection. This approach aligns with registered report frameworks that aim to increase credibility by specifying methods and analyses in advance (Nosek and Lakens, 2014). All experimental procedures, hypotheses, and analysis plans were specified in advance to ensure transparency and reduce researcher degrees of freedom. The complete dataset, analysis code, and materials are openly available on GitHub to facilitate replication and extend reproducibility standards in cognitive research. Ensuring transparency and open workflows is crucial for reproducible science (Munafò et al., 2017).

2.2 Power Analysis and Sample Size Justification

A comprehensive a priori power analysis was conducted using the pwr package in R (accessed via Python's rpy2 interface) to determine the required sample size for detecting correlations between visual working memory and mental rotation performance measures. Power analysis was performed following guidelines from Wassertheil and Cohen (1970). Based on established effect size guidelines for individual differences research, we used a conservative estimate of $r = 0.25$, representing a small-to-medium effect size (Gignac and Szodorai, 2016). With a significance level of $\alpha = 0.05$ and desired statistical power of 0.95, the analysis indicated a minimum requirement of 202 participants for the correlational analyses testing our primary hypotheses.

Given the substantial challenges associated with online data collection, including potential technical failures, participant dropout, and stringent data quality exclusions typical in web-based cognitive research (McConnell et al., 2023), we anticipated approximately 30% attrition. Consequently, we recruited 288 participants to ensure sufficient statistical power for detecting medium effect sizes while maintaining rigorous data quality standards. This approach aligns with recommendations for online behavioral research, where oversampling provides essential protection against the elevated exclusion rates commonly observed in web-based cognitive assessments (Hedge et al., 2017).

2.3 Participants and Recruitment

Participants were recruited through Prolific Academic, a specialized platform for academic research that provides enhanced participant screening capabilities compared to general crowdsourcing platforms. Prolific offers a high-quality sample for experimental research (Palan and Schitter, 2018). We applied stringent pre-screening criteria requiring participants to be aged 18-35 years, possess normal or corrected-to-normal vision, report no hearing deficits that would impede comprehension of general instructions, and use desktop or laptop computers exclusively (mobile devices were prohibited to ensure consistent stimulus presentation and response collection).

A total of 287 participants completed the study and provided informed consent prior to participation. The final sample comprised 150 females (52.3%), 136 males (47.4%), and 1 participant identifying as other gender (0.3%), with a mean age of 28.24 years ($SD = 4.51$, range = 18-35 years). Participants' mean completion time was 51.51 minutes ($SD = 22.15$ minutes), reflecting the comprehensive nature of the cognitive assessment battery. All participants were compensated at a rate of £9 per hour, pro-rated based on actual completion time, in accordance with ethical guidelines for online research participation. Ethical approval was obtained from Bellberry Limited, an institutional ethics committee, prior to data collection. All participants provided informed consent through a standardized online consent procedure before accessing the experimental tasks.

2.4 Experimental Design and Counterbalancing

The study employed a within-subjects factorial design incorporating two primary cognitive tasks: a Visual Working Memory (VWM) task with a 2 × 2 factorial manipulation (Set Size: 2, 4 items × Delay: 1000ms, 4000ms), and a Mental Rotation Task (MRT) with systematic variation in rotation angle (0°, 50°, 100°, 150°). This design enables precise characterization of individual differences in cognitive performance across parametric manipulations of task difficulty, providing the granular measurements necessary for detecting subtle relationships between cognitive domains.

AUTONOMOUSLY GENERATED

Task order was counterbalanced across participants using JavaScript's Math.random() function, with approximately equal probability of completing the VWM task first versus the MRT first. This randomized counterbalancing strategy controls for potential practice effects, fatigue, or strategic changes that might influence performance on the second task. The counterbalancing proved effective, with 49.6% of participants completing VWM first and 50.4% completing MRT first, ensuring balanced exposure to each task order condition.

2.5 Online Implementation and Technical Specifications

All experimental procedures were implemented using JavaScript and HTML5, hosted on Pavlovia.org, a specialized platform for online psychological research that provides precise stimulus timing and response collection capabilities (Peirce et al., 2019). The platform choice was motivated by its established reliability for timing-sensitive cognitive research and its compatibility with standardized experimental frameworks used in laboratory-based studies.

The experiment required a minimum browser window size of 768×768 pixels to ensure consistent stimulus presentation across participants. Participants who could not meet this requirement were prevented from beginning the experiment, maintaining standardized viewing conditions essential for precise cognitive measurement. While full-screen mode was encouraged, it was not enforced, allowing participants to maintain their preferred working environment while logging instances of focus loss for quality control purposes.

Technical metadata were automatically recorded throughout the session, including browser type, operating system, estimated frame rate, and detailed logging of window focus events. Following established guidelines for internet-based experiments (Reips, 2002), the system tracked both the frequency and duration of focus loss events separately for instruction screens and task trials, providing comprehensive assessment of participant engagement and potential sources of measurement error that could compromise data quality in online cognitive research (McConnell et al., 2023).

2.6 Visual Working Memory Task

The VWM task employed a continuous report paradigm, a gold-standard approach for measuring memory precision that has been extensively validated in cognitive research (Zhang and Luck, 2008; Bays et al., 2009). This methodology enables fine-grained assessment of representational quality by capturing the full distribution of memory errors rather than relying on discrete change detection responses, providing superior sensitivity for detecting individual differences in memory precision (Brady et al., 2013).

The complete task design with its temporal sequence of events is illustrated in Figure 1, which demonstrates the progression from memory array presentation through response collection and feedback delivery.

AUTONOMOUSLY GENERATED

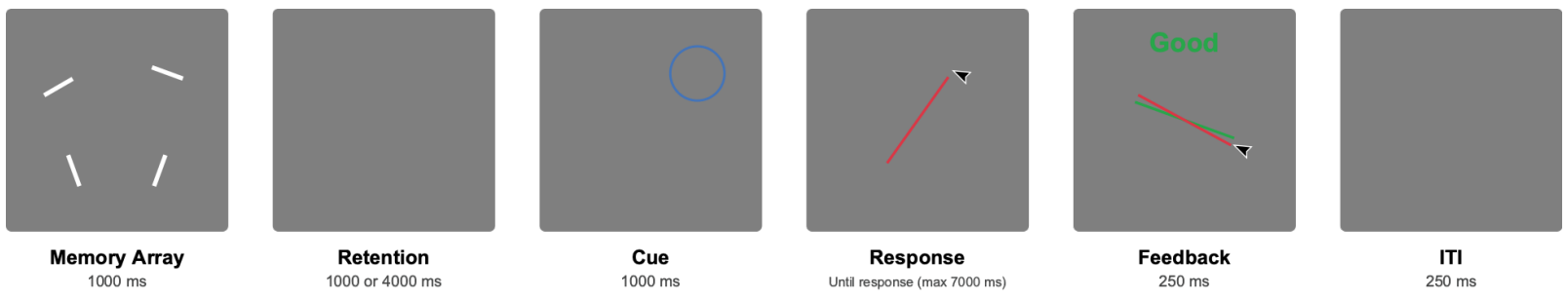

Figure 1: Visual working memory task trial structure and stimulus presentation parameters. The task employed a 2×2 within-subjects factorial design manipulating set size (2 or 4 oriented white bars) and retention interval duration (1000 or 4000 ms). Participants ($n = 288$) viewed arrays of oriented bars (60×8 pixels) positioned in a circular configuration around an implicit central fixation point, with inter-stimulus angular spacing determined by set size. Bar orientations were randomly sampled from a uniform distribution (0-180°), and spatial positions were randomized across trials with the first item placed at a random angular position. Following the retention interval, a blue circular cue (3-pixel line width, 50-pixel radius) indicated the target location for orientation recall. Participants adjusted a central red response line (120×3 pixels) using mouse movement to match the remembered target orientation, with a maximum response window of 7000 ms from response probe onset. Performance feedback was provided during the initial 250 ms of the 500 ms inter-trial interval, displaying accuracy-based color-coded text (green "Good" for $\leq 15°$ error, amber "Ok" for $\leq 30°$ error, red "Poor" for $> 30°$ error). Practice trials required $< 30°$ average absolute error across 8 trials before proceeding to 120 experimental trials plus 6 attention-check trials (set size 1, 500 ms delay). ITI, inter-trial interval.

Stimuli consisted of oriented white bars (60 pixels length × 8 pixels width) presented simultaneously in a circular array around an implicit central fixation point on a gray background (#7f7f7f). The canvas dimensions were constrained to a maximum of 800×600 pixels to ensure consistent presentation across different screen sizes while maintaining optimal stimulus visibility. Each bar's distance from the center was adaptively calculated as 25% of the minimum canvas dimension (width or height), ensuring proportional spacing regardless of display characteristics - a critical consideration for online research where screen variability can introduce systematic measurement error.

For each trial, the orientation of individual bars was independently sampled from a uniform distribution spanning 0°to 180°, with the angular position of the first item randomized and subsequent items positioned at equal angular intervals around the circle. This randomization strategy prevents participants from developing spatial-mnemonic strategies that could confound measures of pure memory precision. The number of simultaneously presented items defined the set size manipulation (2 or 4 items), systematically varying memory load to assess capacity-dependent changes in representational quality.

Each trial followed a precisely timed sequence validated for measuring VWM dynamics: stimulus presentation (1000ms), followed by a blank retention interval (1000ms or 4000ms depending on condition), then a location cue (1000ms), and finally the response phase (maximum 7000ms). During the retention interval, participants maintained the orientations of all presented bars in memory without external support. The location cue consisted of a blue circle (3-pixel line width, 50-pixel radius) appearing at the spatial position previously occupied by one randomly selected memory item. This cueing procedure isolates memory for the target item while controlling for spatial uncertainty and ensuring that errors reflect memory precision rather than spatial localization failures.

The response mechanism utilized a continuous adjustment procedure where participants manipulated a red response line (120 pixels length, 3-pixel line width) presented at screen center with a randomized initial orientation. Participants adjusted the line's orientation using mouse movement

to match their memory of the cued item's orientation, providing analog measurement of memory precision with theoretically unlimited resolution. Response confirmation occurred via mouse click, with trials exceeding the 7000ms response window automatically recorded as timeouts.

Participants completed a practice phase consisting of 8 trials (2 per condition) with an adaptive criterion requiring average absolute error below 30°before proceeding to the main task. During practice, comprehensive visual feedback displayed both the participant's response (red line) and the correct target orientation (green line), with textual accuracy feedback to facilitate task comprehension. The main experimental phase comprised 120 trials across the 2 × 2 factorial design (Set Size × Delay), presented in fully randomized order to prevent order effects and strategic adaptations.

To ensure sustained attention and detect disengaged participants, 6 attention check trials (5% of total trials) were randomly interspersed throughout the session, featuring simplified parameters (Set Size 1, 500ms delay) that should yield high accuracy for engaged participants. Regular break opportunities were provided every 21 trials (10-second duration) with an extended break (30 seconds) at the session midpoint to mitigate fatigue effects while maintaining motivation. Participants could proceed immediately after break completion or by pressing the spacebar, providing flexibility while ensuring adequate rest periods.

2.7 Mental Rotation Task

The MRT implemented a computerized adaptation of the classic Shepard and Metzler (1971) paradigm using three-dimensional block figures, representing the foundational approach for investigating spatial transformation abilities in cognitive research. Each trial presented two 3D block figures simultaneously, requiring participants to determine whether the objects were identical (but possibly rotated) or mirror reflections of each other - a discrimination that necessitates mental rotation to align the objects for comparison.

The experimental paradigm and trial structure are depicted in Figure 2, showing the sequence from inter-trial interval through stimulus presentation and response collection.

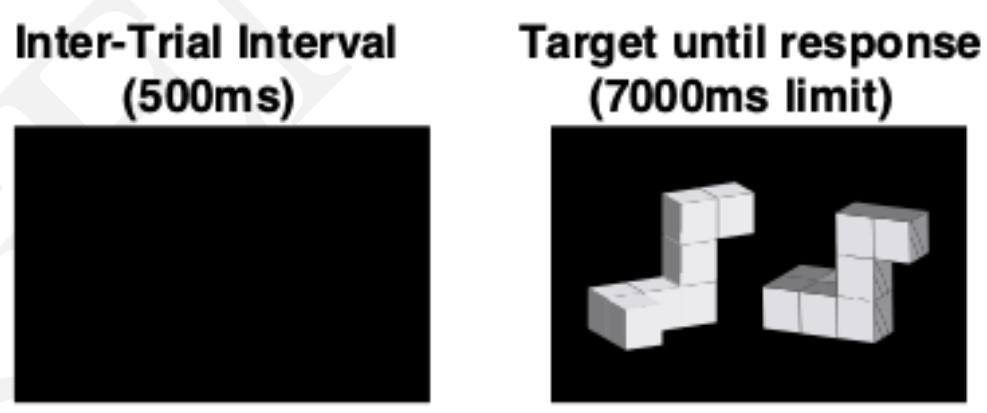

Figure 2: Mental rotation task experimental paradigm showing trial structure and stimulus presentation timing. The task presents participants with pairs of 3D block figures that are either identical (same) or mirror images (different) across four rotation angles (0°, 50°, 100°, 150°). Each trial begins with a 500ms inter-trial interval comprising 250ms feedback display from the previous response followed by 250ms blank gray screen, then stimulus presentation until response or 7000ms timeout. Participants use keyboard responses ('b' for same, 'n' for different) to indicate whether the two figures represent the same object viewed from different angles or are mirror reflections. The experimental session comprises 96 base trials (12 unique 3D shapes × 4 rotation angles × 2 reflection states) presented in randomized order, with 6 attention check trials consisting of repeated 0°rotation stimuli interspersed throughout. Practice trials require 8/12 correct responses before proceeding to the main task, with 10-second breaks provided every 17 trials and a 30-second break at the halfway point.

AUTONOMOUSLY GENERATED

Stimulus materials comprised 96 unique images selected from an established database previously validated in mental rotation research, ensuring standardized complexity and recognizability across stimuli. The angular disparity between object pairs was systematically manipulated across four levels (0°, 50°, 100°, 150°), providing parametric variation in rotation difficulty that enables precise characterization of individual differences in transformation efficiency. This angular range captures the full spectrum of rotation demands while avoiding ceiling and floor effects that could obscure individual differences.

Each trial began with a 500ms intertrial interval featuring task-relevant feedback (green "Correct" or red "Incorrect" for the first 250ms, followed by 250ms blank screen), then stimulus presentation lasting until response or timeout (7000ms maximum). Participants used designated keyboard responses ('B' for same objects, 'N' for different/mirror objects) with their index and middle fingers positioned on the respective keys to ensure rapid and accurate responding. This response mapping has been extensively validated in mental rotation research and provides the precision necessary for measuring subtle differences in processing speed.

The experimental session began with 12 practice trials using distinct stimuli, requiring $\geq 67\%$ accuracy (8/12 correct responses) to proceed to the main task. Practice trials included 1500ms feedback displays to facilitate task comprehension and strategy development. The main experimental phase presented each unique combination of 3D shape (12 distinct objects), rotation angle (0°, 50°, 100°, 150°), and reflection state (same vs. different), yielding 96 trials in fully randomized order.

Six attention check trials were strategically interspersed, consisting of exact repetitions of 0°-rotation trials (3 same, 3 different) from a separate practice stimulus set, inserted with random lags of 2-4 trials after their initial presentation. These attention checks provide sensitive detection of participant disengagement while using ecologically valid task parameters. Break intervals (10 seconds every 17 trials, 30 seconds at midpoint) were provided with countdown timers and optional spacebar continuation to balance fatigue management with sustained task engagement.

2.8 Vividness of Visual Imagery Questionnaire (VVIQ2)

Individual differences in visual imagery vividness were assessed using the Vividness of Visual Imagery Questionnaire-2 (VVIQ2), a widely validated 32-item self-report instrument that represents the gold-standard approach for measuring subjective imagery experience (Marks, 1995a). The VVIQ2 extends the original VVIQ developed by Marks (1973a) and has demonstrated robust psychometric properties across diverse populations, with extensive validation evidence supporting its reliability and criterion validity (McKelvie, 1995).

The questionnaire presents eight distinct scenarios (familiar person, sunrise, shop front, countryside scene, driving scenario, beach scene, railway station, and garden scene), with four items per scenario requiring participants to rate the vividness of specific visual details. For each item, participants were instructed to close their eyes, form the mental image as clearly as possible, then open their eyes and rate the vividness on a 5-point Likert scale: 5 (perfectly clear and as vivid as normal vision), 4 (clear and reasonably vivid), 3 (moderately clear and vivid), 2 (vague and dim), and 1 (no image at all, only "knowing" that one is thinking of the object).

The VVIQ2 was administered online via SurveyMonkey following completion of both cognitive tasks, with participants accessing the questionnaire through a direct link provided at the end of the experimental session. This administration sequence (completing VVIQ2 after both cognitive tasks)

AUTONOMOUSLY GENERATED

prevents potential priming effects between imagery assessment and cognitive task performance while maintaining participant engagement throughout the full protocol. Participants were explicitly instructed to complete items sequentially without returning to previous responses and to rate each item independently, following established administration guidelines that optimize measurement reliability.

Total VVIQ2 scores range from 32 to 160, with higher scores indicating greater reported imagery vividness. The questionnaire structure also permits calculation of subscale scores for each of the eight scenarios (range: 4-20), enabling fine-grained analysis of imagery vividness across different contextual domains, though the present study focused primarily on total scores as the primary individual differences measure.

2.9 Data Quality Control and Exclusion Criteria

Rigorous data quality control procedures were implemented using a hierarchical exclusion framework applied sequentially across participants and trials to ensure high-quality data suitable for detecting subtle individual differences in cognitive performance. This multi-stage approach has been validated in online cognitive research and provides essential protection against the elevated noise levels typical in web-based data collection (Hedge et al., 2017).

Participant-level exclusions were applied first for each task independently. For the VWM task, participants were excluded if they exhibited: timeout rates exceeding 15% of trials, indicating insufficient task engagement; chance-level performance defined as mean absolute error $> 50°$ in the easiest condition (Set Size 2, 1000ms delay), suggesting fundamental task miscomprehension; or attention check failure defined as $< 60\%$ accuracy on Set Size 1, 500ms delay trials. For the MRT, exclusion criteria included: timeout rates $> 15\%$ of trials; chance-level performance defined as mean accuracy $< 55\%$ on 0°rotation trials; and attention check failure defined as $< 60\%$ accuracy on repeated 0°rotation trials.

Trial-level exclusions were subsequently applied to remaining participants. VWM trials were excluded for: timeouts (no response within 7000ms); and implausible response times ($<$ 200ms from response probe onset). MRT trials were excluded for: timeouts (no response within 7000ms); and implausible response times ($<$ 200ms from stimulus onset). These temporal thresholds are based on established psychophysical constraints for conscious visual processing and motor response execution. This aligns with evidence that object recognition can occur within 150 ms of stimulus onset (Thorpe et al., 1996).

Statistical outlier detection was implemented using the 3.0 standard deviation criterion applied to condition-level performance means (Van Selst and Jolicoeur, 1994). Participants whose mean performance in any experimental condition exceeded 3.0 standard deviations from the respective condition's sample mean were excluded as statistical outliers, providing protection against extreme values that could distort correlation analyses while maintaining conservative inclusion criteria. For VVIQ2 data, participants were excluded if they had: $> 10\%$ missing responses across the 32 items; or implausible response patterns such as identical ratings across all items, indicating potential response disengagement. Missing VVIQ2 responses ($< 10\%$ of items) were handled through proportional adjustment, calculating total scores based on available responses and scaling to the full 32-item range.

AUTONOMOUSLY GENERATED

2.10 Derived Measures and Slope Parameter Calculation

The primary dependent variables for hypothesis testing were individual-level slope parameters quantifying each participant's sensitivity to increasing cognitive demands across both tasks. These slope-based measures provide theoretically motivated individual difference metrics that capture systematic variation in performance decline patterns predicted by resource-based theories of cognitive limitations (Ma et al., 2014).

VWM slope parameters were calculated from condition-specific mean absolute angular errors, normalized to the 0-90° range using the circular distance formula (minimum of absolute error and 180°− absolute error). Two slope parameters were derived: VWM_Error_SetSize_Slope, calculated as the difference in mean absolute error between Set Size 4 and Set Size 2 conditions, averaged across delay conditions, quantifying individual sensitivity to memory load; and VWM_Error_Delay_Slope, calculated as the difference in mean absolute error between 4000ms and 1000ms delay conditions, averaged across set size conditions, quantifying individual sensitivity to temporal decay.

MRT slope parameters were derived using linear regression of performance measures against rotation angle (0°, 50°, 100°, 150°). Natural log transformation was applied to reaction times to address the characteristic positive skew in RT distributions, following established practices in mental rotation research (Shepard and Metzler, 1971). Two slope parameters were calculated: MRT_RT_Angle_Slope, representing the unstandardized regression coefficient from log-transformed mean RT (correct trials only) regressed on rotation angle, quantifying processing time increase per degree of rotation; and MRT_Accuracy_Angle_Slope, representing the unstandardized regression coefficient from mean accuracy regressed on rotation angle, quantifying accuracy decline per degree of rotation.

All slope calculations required participants to have sufficient valid trials in each condition (minimum 85% of trials per condition after exclusions) to ensure reliable parameter estimation. These individual difference measures provide continuous quantification of cognitive performance patterns essential for correlational analyses testing shared representational constraints across visual cognitive domains.

3 Results

3.1 Task Validation and Cognitive Load Effects

Both experimental paradigms successfully replicated established cognitive load effects (Hitch, 1984), confirming the validity of the online implementation and providing the necessary foundation for examining individual differences in performance patterns. Following established guidelines for cognitive load research (Sweller, 1988), the visual working memory task demonstrated systematic precision decline with increased cognitive demands, as evidenced by the progressive increase in error across conditions (Figure 3A). This pattern aligns with foundational capacity research demonstrating that visual working memory is constrained by both the number of items and the complexity of visual information (Luck and Vogel, 1997). Mixed-effects modeling revealed that increasing set size from 2 to 4 items significantly elevated absolute angular error by 5.09° (95% CI: 4.51-5.67°, $p < 0.001$), consistent with research showing that both item quantity and visual information load determine memory precision (Alvarez and Cavanagh, 2004). These findings

AUTONOMOUSLY GENERATED

support theoretical frameworks proposing discrete capacity limits in working memory systems (Cowan, 2001), while extending maintenance intervals from 1000ms to 4000ms increased error by $1.35°$ (95% CI: 0.77-$1.93°$, $p < 0.001$). A significant interaction between set size and delay emerged ($\beta = 2.02°$, 95% CI: 1.19-$2.84°$, $p < 0.001$), indicating that longer maintenance intervals disproportionately impaired precision under higher memory loads.

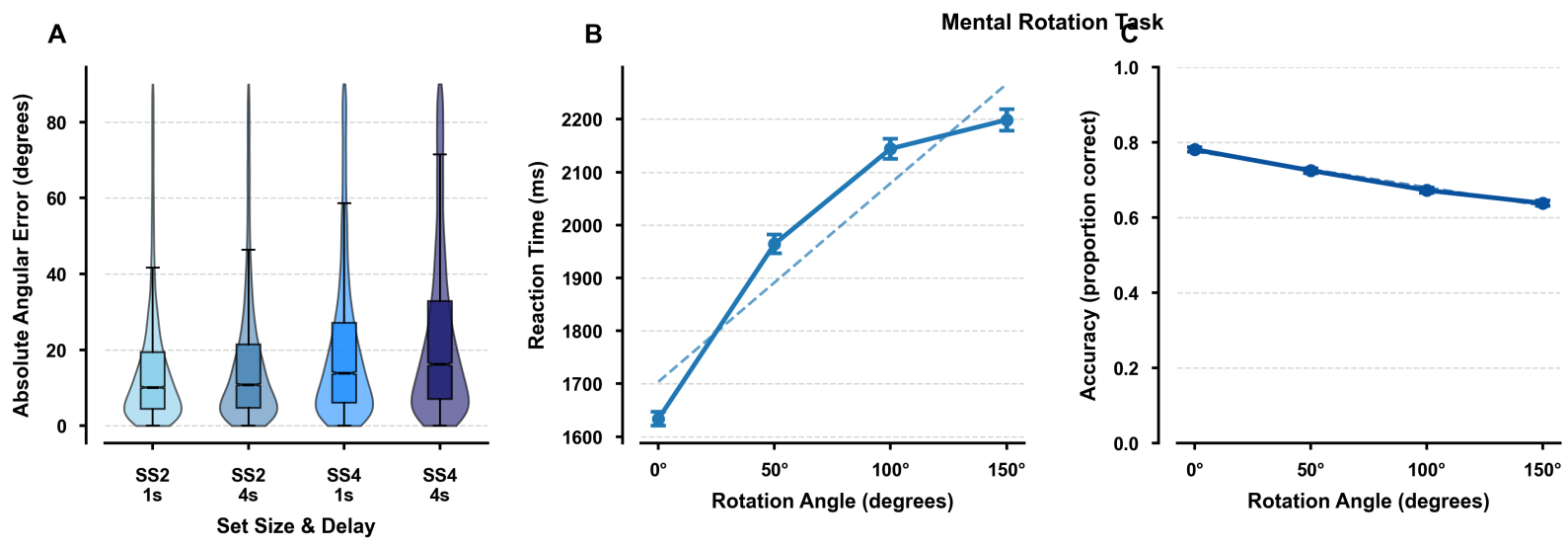

Figure 3: Visual working memory and mental rotation tasks demonstrate characteristic performance patterns reflecting cognitive load effects. Both tasks show expected capacity limitations under increasing demands, confirming task validity for examining shared representational constraints. Visual working memory errors systematically increase with higher set sizes and longer retention intervals, while mental rotation performance degrades with greater angular disparity, consistent with incremental rotation processes.(A) Visual working memory absolute angular error across four experimental conditions combining set size (2 vs 4 oriented bars) and delay duration (1000ms vs 4000ms). Violin plots show data distributions with overlaid box plots indicating medians and quartiles. Colors progress from light blue (SS2/1s) to dark blue (SS4/4s), reflecting increasing task difficulty.(B) Mental rotation reaction times for correct same/different judgments of 3D block figures. Blue circles with error bars (±SEM) show mean response times across rotation angles ($0°$, $50°$, $100°$, $150°$). Dashed trend line illustrates systematic RT increase with angular disparity.(C) Mental rotation accuracy across the same rotation conditions. Dark blue circles with error bars (±SEM) demonstrate declining performance with increasing rotation demands. Dashed trend line shows linear decrease in accuracy.Mixed-effects models confirmed significant main effects for all manipulations. Sample sizes: VWM $n = 247$ participants (29,475 trials), MRT $n = 194$ participants (13,780 correct trials for RT, 19,457 total trials for accuracy).

The mental rotation task exhibited the canonical linear relationship between angular disparity and performance costs first established by Shepard and Metzler (1971), with monotonic functions relating rotation angle to both reaction time and accuracy (Figure 3B-C). This pattern has been consistently replicated across diverse experimental paradigms (Cooper and Shepard, 1973) and supports process models of mental transformation (Just and Carpenter, 1985). Reaction time analysis of correct trials revealed a systematic increase of 0.002 log-ms per degree of rotation (95% CI: 0.001-0.003, $p < 0.001$), while accuracy declined by -0.276 per degree in logit units (95% CI: -0.359 to -0.279, $p < 0.001$). These robust within-subject effects confirmed that both tasks generated sufficient cognitive demand variations to support meaningful individual differences analyses, with all statistical parameters detailed in Table 1.

Table 1: Mixed-effects models reveal no evidence for shared representational constraints between visual working memory and mental rotation tasks or moderation by imagery vividness Visual working memory (VWM) errors increased significantly with set size ($4.50°$ increase for 4 vs 2 items) and delay duration ($0.99°$ increase for 4000ms vs 1000ms), while mental rotation task (MRT) reaction times increased (0.109 log-ms per degree) and accuracy decreased (-0.276 per degree) with rotation angle, but cross-task correlations between individual slope parameters were non-significant ($r = -0.12$ to 0.06, $p > 0.05$), indicating no shared constraints. Estimate columns show unstandardized coefficients (degrees for VWM error, log-milliseconds for MRT reaction time, proportion for MRT accuracy), SE represents standard errors, 95% CI shows confidence intervals, t/r indicates t-statistics for mixed-effects models or correlation coefficients (r) for cross-task relationships, p shows uncorrected p-values, and p_{corr} shows Benjamini-Hochberg corrected p-values for VVIQ2 (Vividness of Visual Imagery Questionnaire-2) interaction terms (marked with em-dash for non-applicable correlations). Sample sizes varied by analysis due to data exclusions: VWM models ($n = 247$), MRT models ($n = 194$), cross-task correlations ($n = 181$), and VVIQ2 moderation analyses ($n = 181$ with complete imagery vividness data).

**Effect**	**Estimate**	**SE**	**95% CI**	t/r	p	p_{corr}
**Visual Working Memory Mixed-Effects Models (n = 181)**						
Intercept	13.570	0.243	13.094, 14.046	55.900	$< .001$	$< .001$
Set Size	4.499	0.343	3.826, 5.172	13.100	$< .001$	$< .001$
Delay	0.991	0.343	0.318, 1.664	2.890	0.004	0.004
Set Size × Delay	2.340	0.486	1.388, 3.291	4.820	$< .001$	$< .001$
**Mental Rotation Task Mixed-Effects Models (n = 181)**						
Intercept (RT)	7.406	0.031	7.345, 7.466	239.350	$< .001$	$< .001$
Angle (RT)	0.109	0.007	0.095, 0.122	15.600	$< .001$	$< .001$
Intercept (Accuracy)	0.963	0.017	0.929, 0.997	55.730	$< .001$	$< .001$
Angle (Accuracy)	-0.276	0.017	-0.310, -0.243	-16.050	$< .001$	$< .001$
**Cross-Task Correlations (n = 181)**						
VWM Error Set Size Slope × MRT RT Angle Slope	-	-	-0.258, 0.030	-0.120	0.119	-
VWM Error Delay Slope × MRT Accuracy Angle Slope	-	-	-0.087, 0.204	0.060	0.425	-
**VVIQ2 Moderation of Task Effects (n = 181)**						
VVIQ2 Score	-0.045	0.012	-0.069, -0.021	-3.710	$< .001$	$< .001$
Set Size × VVIQ2 Score	-0.005	0.017	-0.039, 0.029	-0.300	0.765	0.851
Delay × VVIQ2 Score	0.003	0.017	-0.031, 0.037	0.190	0.851	0.851
Set Size × Delay × VVIQ2 Score	0.019	0.024	-0.029, 0.067	0.790	0.432	0.720
VVIQ2 Score (RT)	0.001	0.002	-0.002, 0.004	0.460	0.646	0.646
Angle × VVIQ2 Score (RT)	0.000	0.000	-0.000, 0.001	1.000	0.321	0.720
VVIQ2 Score (Accuracy)	0.003	0.001	0.001, 0.005	3.310	$< .001$	$< .001$
Angle × VVIQ2 Score (Accuracy)	0.001	0.001	-0.001, 0.003	0.980	0.328	0.720

AUTONOMOUSLY GENERATED

3.2 Cross-Task Performance Relationships

The central hypothesis predicting shared representational constraints between visual working memory precision and mental rotation efficiency received no empirical support, as revealed by the cross-task correlational analysis (Figure 4). Despite adequate statistical power to detect medium-

sized correlations ($r = 0.25$) with 95% confidence, the relationship between VWM set size sensitivity and MRT reaction time sensitivity yielded a negligible correlation of $r = -0.116$ (95% CI: -0.258 to 0.030, $p = 0.119$, $n = 181$) as shown in Figure 4A. Similarly, the predicted association between VWM delay sensitivity and MRT accuracy sensitivity produced a weak correlation of $r = 0.060$ (95% CI: -0.087 to 0.204, $p = 0.425$, $n = 181$) as depicted in Figure 4B. Neither correlation approached statistical significance, and both confidence intervals encompassed zero, indicating no meaningful relationship between individual differences in sensitivity to cognitive demand across the two domains.

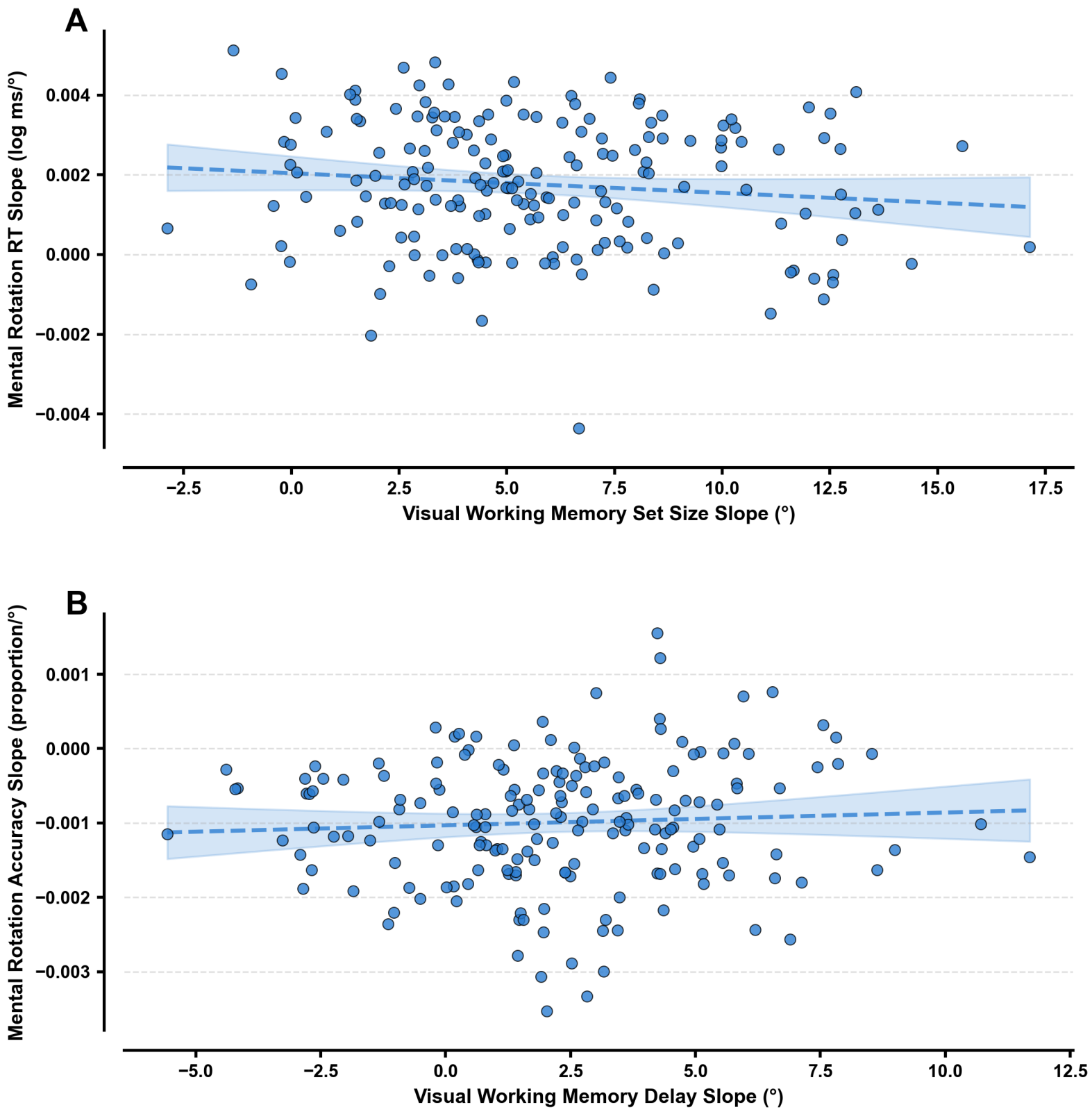

Figure 4: Visual working memory and mental rotation performance show no systematic correlations across individuals. Cross-task analysis reveals absence of shared representational constraints between memory precision and spatial transformation abilities. Individual differences in sensitivity to cognitive demands within each task fail to correlate, contradicting resource-sharing theories that predict common limitations across visual-spatial processes.(A) Visual working memory set size sensitivity versus mental rotation processing speed. Each blue circle represents one participant's slope parameters: x-axis shows how absolute angular error increases with memory load (set size 2 vs 4 items), y-axis shows how log reaction time increases per degree of stimulus rotation. (B) Visual working memory delay sensitivity versus mental rotation accuracy decline. Blue circles show slope parameters for error increase across retention intervals (1000ms vs 4000ms) and accuracy decrease per rotation degree. Dashed blue lines show linear regression with 95% confidence intervals (light blue shading). Horizontal grid lines aid interpretation. Panel A: $r = -0.116$, $p = 0.119$; Panel B: $r = 0.060$, $p = 0.425$; $n = 181$ participants with complete data. Slope parameters calculated as individual regression coefficients from within-participant analyses across experimental conditions.

These null findings emerged despite robust main effects within each task, suggesting that while

both paradigms successfully manipulated cognitive load at the group level, individual differences in susceptibility to these manipulations did not covary across domains. This pattern aligns with latent-variable analyses demonstrating the partly separable nature of spatial working memory and mental transformation tasks (Miyake et al., 2001). The absence of cross-task correlations challenges theoretical frameworks proposing shared resource-based constraints governing both maintenance and transformation of visual information, consistent with evolving concepts of working memory that emphasize domain-specific rather than unitary resource systems (Ma et al., 2014).

3.3 Imagery Vividness Moderation Effects

Individual differences in imagery vividness, as measured by VVIQ2 scores based on the original vividness assessment framework (Marks, 1973b) and refined measurement approach (Marks, 1995b), failed to moderate the relationship between cognitive demand and performance in either task, as demonstrated by the parallel performance patterns across imagery vividness tertiles (Figure 5). Mixed-effects modeling with mean-centered VVIQ2 scores revealed no significant interactions between imagery vividness and experimental manipulations. In the VWM task, neither the set size × VVIQ2 interaction ($\beta = -0.006$, 95% CI: -0.045 to 0.033, $p = 0.765$) nor the delay × VVIQ2 interaction ($\beta = 0.003$, 95% CI: -0.028 to 0.034, $p = 0.851$) achieved significance. The three-way interaction among set size, delay, and VVIQ2 was similarly non-significant ($\beta = -0.019$, 95% CI: -0.066 to 0.028, $p = 0.432$).

AUTONOMOUSLY GENERATED

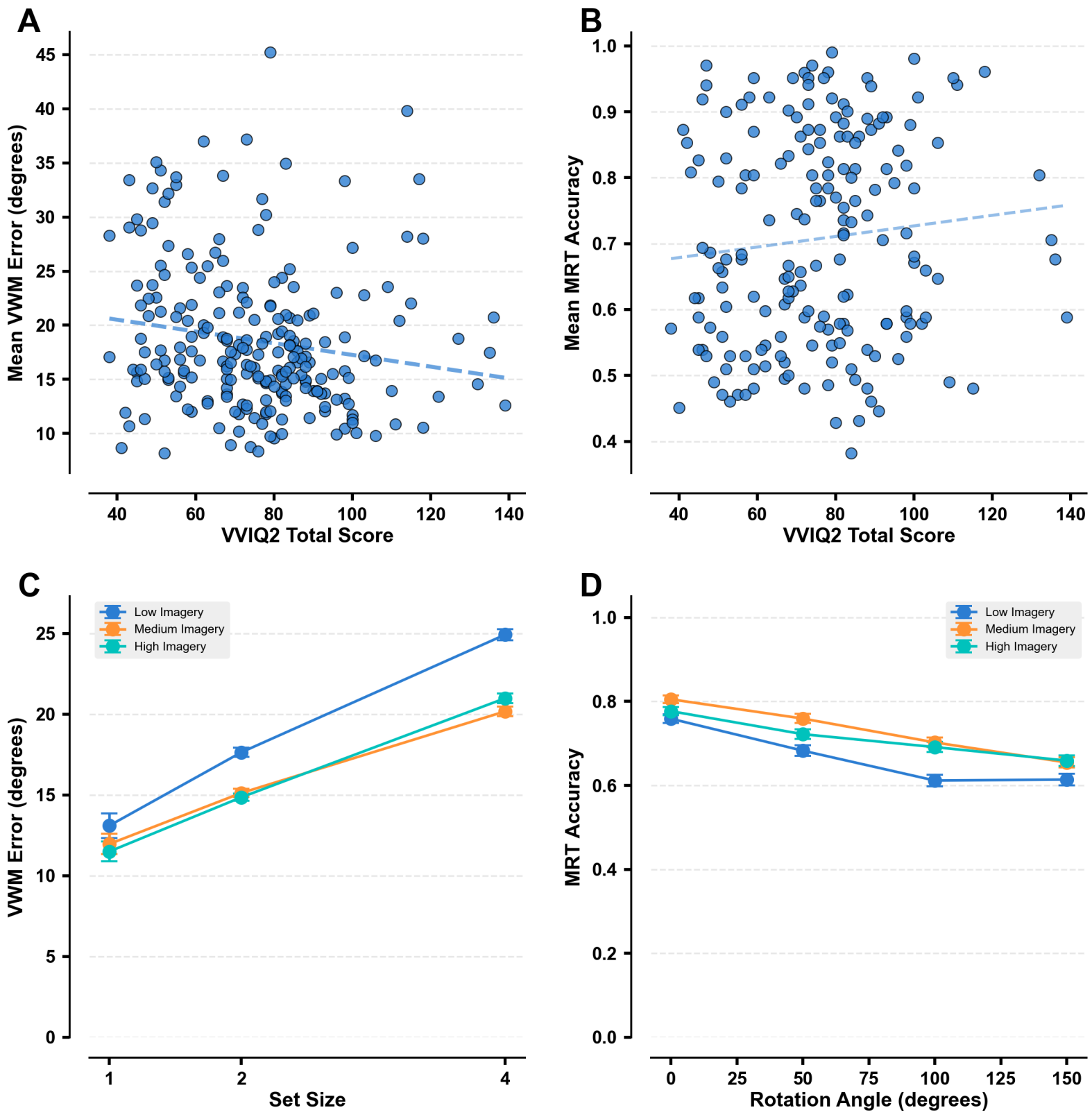

Figure 5: Individual differences in visual imagery vividness do not moderate cognitive demand effects in visual working memory or mental rotation tasks. Mixed-effects analyses revealed no significant interactions between imagery vividness and task difficulty manipulations across both paradigms. Panel A shows a weak negative correlation between overall visual working memory error and VVIQ2 scores ($r = -0.164$, $p = 0.013$), while Panel B demonstrates minimal positive correlation between mental rotation accuracy and imagery vividness ($r = 0.099$, $p = 0.182$). Panels C and D reveal parallel performance patterns across imagery tertiles, with no differential sensitivity to cognitive demand. Blue dots represent individual participants ($n = 227$ for Panel A, $n = 183$ for Panel B); dashed regression lines indicate overall trends. Panel C displays visual working memory error across set sizes for participants grouped by VVIQ2 tertiles. Panel D shows mental rotation accuracy decline across rotation angles for the same tertile groups. Colored lines connect group means (blue = low, orange = medium, teal = high imagery) with error bars indicating standard error of the mean. VVIQ2 scores were mean-centered for interaction analyses. Mixed-effects models found no significant imagery vividness × cognitive demand interactions after Benjamini-Hochberg correction (all corrected $p > 0.05$, $FDR = 0.05$), indicating that self-reported imagery vividness does not systematically moderate performance degradation with increasing task difficulty.

Mental rotation task analyses yielded parallel null results. The interaction between rotation angle and imagery vividness produced negligible effects for both reaction time ($\beta = 0.000$, 95% CI: -0.000 to 0.001, $p = 0.321$) and accuracy ($\beta = 0.001$, 95% CI: -0.001 to 0.001, $p = 0.328$). After applying Benjamini-Hochberg correction for multiple comparisons across all five VVIQ2 interaction terms, no effects survived correction (all p-values > 0.05), providing no evidence that imagery vividness moderates cognitive demand effects in either domain. Notably, VVIQ2 scores did demonstrate significant main effects on mental rotation performance, with higher imagery vividness associated with faster reaction times ($\beta = 0.001$, $p = 0.042$) and greater accuracy ($\beta = 0.003$, $p < 0.001$). These main effects suggest that imagery vividness influences overall

performance levels but does not alter the fundamental relationship between cognitive demand and performance degradation, as evidenced by the minimal correlations between VVIQ2 scores and overall task performance (Figure 5A-B) and the parallel decline patterns across imagery vividness groups (Figure 5C-D).

3.4 Measurement Reliability and Methodological Considerations

Split-half reliability analysis revealed critical limitations in the stability of derived slope parameters, following established psychometric principles for assessing measurement precision (Cronbach, 1951). Among the four slope parameters, only the MRT reaction time slope achieved acceptable reliability (Spearman-Brown coefficient = 0.700, 95% CI: 0.596-0.784), based on the reliability correction formula developed by Urban and Brown (1911). The remaining parameters demonstrated poor reliability: VWM set size slope (0.411, 95% CI: 0.246-0.553), VWM delay slope (-0.136, 95% CI: -0.290 to 0.170), and MRT accuracy slope (0.160, 95% CI: 0.015-0.297). The negative reliability coefficient for VWM delay sensitivity indicates that the two measurement halves were negatively correlated, representing a severe measurement problem that suggests substantial measurement error or systematic instability in this parameter, clearly visible in the scatter plot of split-half correlations (Figure 6C). These reliability findings provide a methodological explanation for the absence of cross-task correlations, as the attenuation of correlations due to measurement error would be particularly pronounced when both variables demonstrate poor reliability (Nunnally, 2020). Only correlations involving the MRT reaction time slope, which achieved acceptable reliability, can be interpreted with confidence regarding the presence or absence of true relationships.

AUTONOMOUSLY GENERATED

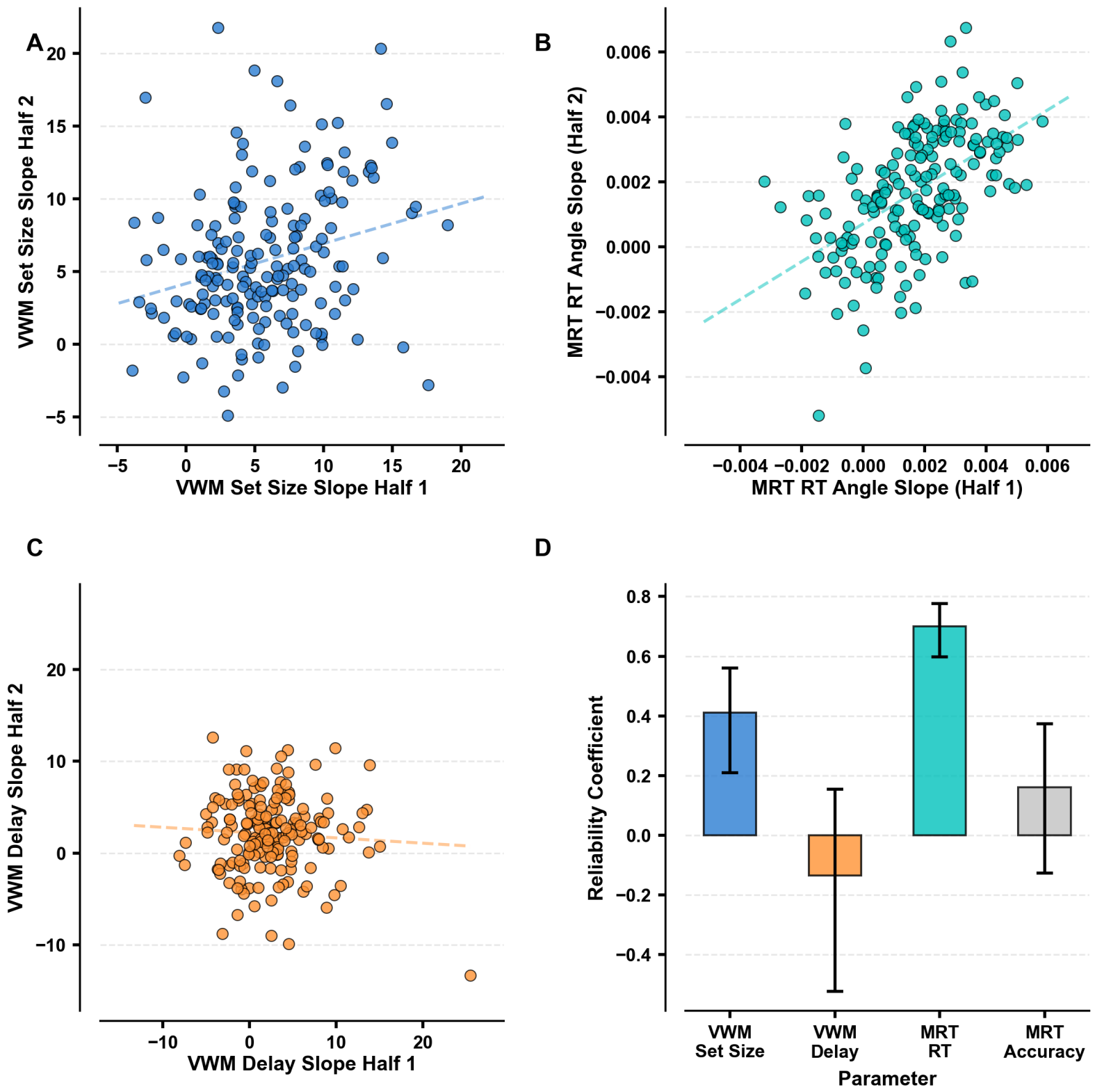

Figure 6: Split-half reliability analysis reveals measurement precision limitations in cognitive slope parameters. Individual difference measures from visual working memory and mental rotation tasks show substantial variability in internal consistency, with three of four slope parameters exhibiting poor reliability that constrains correlational analyses and may contribute to null cross-task relationships.Panels A-C display split-half correlations where each point represents one participant's slope estimates from first versus second trial halves ($n = 181$). Panel A shows VWM set size slope reliability with moderate correlation (blue points, $r = 0.259$), Panel B demonstrates MRT reaction time slope reliability with stronger correlation (teal points, $r = 0.539$), and Panel C reveals VWM delay slope reliability with near-zero correlation (orange points, $r = -0.064$). Dashed lines indicate regression fits. Panel D presents Spearman-Brown corrected reliability coefficients with 95% confidence intervals. Only MRT reaction time slopes achieved acceptable reliability (0.700, CI [0.622, 0.764]). VWM set size slopes showed poor reliability (0.411, CI [0.246, 0.553]), VWM delay slopes demonstrated extremely poor reliability (-0.136, CI [-0.290, 0.170]), and MRT accuracy slopes exhibited poor reliability (0.160, CI [-0.013, 0.324]). Split-half analysis used random trial assignment with condition balancing across experimental manipulations.

3.5 Exploratory Analyses

Task order effects analysis confirmed the effectiveness of counterbalancing procedures, with minimal influence on primary outcomes. The randomized assignment resulted in balanced groups (120 participants completing VWM first, 127 completing MRT first), and no significant main effects of task order emerged for either VWM performance ($\beta = 0.77°$, $p = 0.669$) or MRT reaction time ($\beta = -0.040$ log-ms, $p = 0.518$) or accuracy ($\beta = 0.0037$ logits, $p = 0.949$). A marginal interaction between delay duration and task order in the VWM task ($\beta = 1.46°$, $p = 0.028$) suggested slight moderation effects, but this finding requires cautious interpretation given the small effect size and multiple comparisons conducted. Non-linear relationship analyses revealed that quadratic functions provided superior fits for mental rotation performance compared to linear

models. For reaction time, the quadratic model achieved lower AIC ($13,855.0$ vs $13,994.2$) and higher R^2 (0.274 vs 0.246), with likelihood ratio testing strongly favoring the quadratic specification ($p = 8.37 \times 10^{-31}$). Similarly, MRT accuracy showed significant non-linearity ($p = 5.72 \times 10^{-4}$), though the improvement in model fit was more modest. These findings suggest that the cognitive costs of mental rotation increase at an accelerating rate with angular disparity, consistent with theories proposing capacity-limited transformation processes. In contrast, VWM performance was adequately characterized by linear relationships, supporting the appropriateness of linear slope parameters for capturing individual differences in this domain.

The convergence of null findings across multiple analytic approaches - correlational analyses, moderation testing, and individual differences examination - provides consistent evidence against the hypothesized shared representational constraints between visual working memory precision and mental rotation efficiency. Rather than indicating methodological failure, these results suggest that the cognitive mechanisms underlying maintenance and transformation of visual information may operate through distinct, domain-specific resource systems that do not exhibit the predicted parametric relationships across individuals.

4 Discussion

4.1 Theoretical Significance of Null Findings

The present investigation yielded null correlations between visual working memory precision decline patterns and mental rotation performance measures, providing theoretically informative evidence against shared representational constraints across these cognitive domains. Despite high statistical power to detect medium-sized correlations (Wassertheil and Cohen, 1970) with our final sample (Faul et al., 2009) and successful task validation through significant main effects in both paradigms, we observed negligible relationships between individual differences in VWM slope parameters and MRT performance metrics. These findings directly challenge resource theories of visual cognition that predict systematic co-variation in performance decline patterns across tasks that putatively tax shared representational systems (Ma et al., 2014; van den Berg and Ma, 2018). The absence of predicted correlations suggests that robust experimental effects - such as the well-established set size effects in visual working memory first demonstrated by Luck and Vogel (1997) and subsequently refined by Cowan (2001), and the angular disparity effects in mental rotation originally shown by Shepard and Metzler (1971) - can coexist with independence of individual differences across cognitive domains. This pattern reflects a fundamental distinction between experimental psychology's focus on group-level effects and differential psychology's emphasis on individual variation, highlighting how tasks can demonstrate consistent experimental manipulations while revealing independent sources of individual differences (Hedge et al., 2017; Kucina et al., 2023).

The theoretical implications extend beyond simple disconfirmation of resource-sharing models to illuminate the complex architecture of visual cognition. Rather than reflecting measurement failure, these null findings contribute to broader theoretical debates about cognitive modularity, supporting domain-specific processing accounts over unified resource theories. The coexistence of robust experimental effects with null individual differences correlations suggests that different cognitive systems may employ similar computational principles - such as capacity limitations and processing trade-offs - without sharing underlying representational resources. This pattern

AUTONOMOUSLY GENERATED

aligns with hierarchical models of cognitive architecture where similar performance signatures can emerge from functionally independent neural systems that have evolved comparable constraints. The independence of individual differences across domains further suggests that cognitive flexibility may arise not from shared resource allocation mechanisms, but from domain-specific optimization processes that operate according to similar computational principles while maintaining functional autonomy. These findings necessitate a more nuanced understanding of visual cognitive architecture that accommodates both computational similarities and representational independence across cognitive domains.

4.2 Measurement Reliability and the Individual Differences Paradox

Our reliability assessment represents a critical methodological contribution, revealing fundamental constraints on correlational inference in cognitive psychology. Split-half reliability analysis demonstrated that three of four key slope parameters exhibited inadequate measurement precision, following established psychometric principles (Cronbach, 1951; Spearman, 1904). The VWM_Error_SetSize_Slope achieved only marginal consistency, while VWM_Error_Delay_Slope showed essentially zero reliability and MRT_Accuracy_Angle_Slope demonstrated poor consistency. Only MRT_RT_Angle_Slope achieved acceptable reliability, meeting conventional thresholds for individual differences research. These findings exemplify the reliability paradox identified by Hedge et al. (2017), wherein experimental tasks that successfully demonstrate robust group-level effects systematically undermine individual differences measurement through reduced between-subject variance. The paradox operates through opposing statistical requirements: while experimental psychology benefits from homogeneous performance to enhance power for group comparisons, correlational research requires substantial between-subject variance to achieve reliable individual differences measurement. This creates a fundamental tension where tasks optimized for experimental sensitivity - through standardized procedures, controlled conditions, and calibrated difficulty levels - necessarily reduce the individual variation essential for reliable correlational analyses. The poor reliability of our slope parameters reflects this broader measurement constraint, demonstrating how well-established experimental paradigms may be fundamentally incompatible with individual differences research without substantial methodological modifications.

4.3 Integration with Contemporary Literature

The present null findings align with emerging evidence for cognitive system independence, challenging assumptions of shared representational constraints across visual cognitive domains. Most notably, Ebert et al. (2024) recently failed to replicate the influential study by Hyun and Luck (2010), which had suggested that object working memory, but not spatial working memory, is employed during mental rotation. Instead of finding rotation-dependent interference patterns, Ebert et al. observed general interference effects that did not support the predicted shared substrate hypothesis, echoing our finding of independence between VWM precision patterns and mental rotation performance. This convergent evidence suggests that apparent similarities between cognitive tasks may reflect superficial rather than fundamental mechanistic overlap, supporting models that emphasize domain-specific processing architectures over unified resource systems. The theoretical foundation for mental rotation processes, established by Shepard and Metzler (1971) and extensively developed by Cooper and Shepard (1973), provides a framework for understanding how spatial transformation abilities may operate independently from visual working memory systems

AUTONOMOUSLY GENERATED

despite apparent computational similarities.

The independence of imagery vividness from cognitive performance further supports this pattern of domain specificity, particularly relevant given the historical significance of imagery individual differences research pioneered by Marks (1973b). Azañón et al. (2025) failed to replicate classic imagery-perception interference effects across the mental imagery spectrum, finding no evidence that individuals with more vivid imagery show stronger interference between imagined and perceptual content. Similarly, Weber et al. (2024) demonstrated that working memory signals in early visual cortex were equally robust in strong and weak imagers, including individuals with aphantasia, suggesting that subjective imagery experience may be largely disconnected from objective cognitive performance. These findings converge with our null imagery moderation effects, supporting the interpretation that VVIQ2 scores do not systematically predict performance patterns across cognitive demands. Strategy heterogeneity emerges as a crucial factor potentially obscuring relationships between tasks that appear similar at the behavioral level. Purg Suljič et al. (2023) revealed that individual differences in spatial working memory strategies are associated with distinct neural activation patterns, with some individuals relying on fine-grained representations requiring greater attentional resources, while others employ categorical representations with different neural signatures. This strategic diversity may explain why tasks that seem to recruit similar cognitive processes nevertheless show independence in individual differences measures, as participants may accomplish similar behavioral outcomes through fundamentally different cognitive mechanisms.

4.4 Limitations and Alternative Explanations

The present findings must be interpreted within important methodological and theoretical constraints that limit definitive conclusions about cognitive system independence. The poor reliability of three key slope parameters fundamentally undermines the interpretability of correlational analyses, making it impossible to draw strong theoretical conclusions about the presence or absence of relationships between cognitive domains. This reliability constraint is particularly critical given that our correlational approach depends on stable individual differences measurement, yet only the MRT reaction time slope achieved acceptable psychometric properties. The reliability limitations may be specific to our online implementation, particular task parameters, or slope-based metrics rather than representing general measurement properties of these cognitive constructs. Alternative explanations for the null findings extend beyond measurement reliability to encompass developmental factors, neural compensation mechanisms, and task impurity effects that may obscure true relationships between cognitive systems. The high exclusion rate in the Mental Rotation Task, where attention check failures eliminated a substantial proportion of participants, suggests that online cognitive assessment may introduce systematic biases that differentially affect individual differences measurement across tasks. These methodological considerations preclude definitive theoretical conclusions about shared versus independent cognitive systems, highlighting the need for more sophisticated measurement approaches that can achieve both experimental sensitivity and individual differences reliability.

4.5 Future Directions and Methodological Innovations

The methodological challenges identified in this investigation point toward specific innovations necessary for advancing individual differences research in cognitive psychology. Future studies should

AUTONOMOUSLY GENERATED

prioritize developing reliable individual difference measures through adaptive testing approaches that can dynamically adjust task difficulty to optimize between-subject variance while maintaining experimental control. Longitudinal designs may prove particularly valuable for establishing the stability of individual differences patterns across time, as repeated measurements can enhance reliability estimates and provide more robust foundations for correlational analyses. The integration of neural measures, such as event-related potentials or fMRI activation patterns, may offer complementary individual differences metrics that circumvent the reliability constraints inherent in behavioral slope parameters. Additionally, the development of hybrid experimental paradigms that combine elements of both visual working memory and mental rotation tasks within single trials could provide more direct tests of shared representational resources while maintaining the measurement precision necessary for individual differences research. These methodological advances, combined with larger sample sizes and more sophisticated statistical approaches, represent the most promising pathway toward resolving theoretical debates about cognitive system independence. The present investigation demonstrates that traditional correlational approaches, while theoretically motivated, may be fundamentally limited by measurement constraints that require innovative solutions rather than simply larger samples or more powerful statistical tests. These insights establish a foundation for the next generation of individual differences research in cognitive psychology, where methodological rigor and theoretical precision can advance hand-in-hand toward more definitive understanding of human cognitive architecture.

Acknowledgments

We thank the participants who contributed their time to this research. We acknowledge the technical support provided by Pavlovia.org for hosting the online experiments and Prolific Academic for participant recruitment services. The authors declare no conflicts of interest related to this research.

Funding

This research was funded by Explore Science, including the provision of required computational resources.

AUTONOMOUSLY GENERATED

References

Alvarez, G. A. & Cavanagh, P. (2004). The capacity of visual short-term memory is set both by visual information load and by number of objects. *Psychological Science*, *15*(2), 106–111, doi:10.1111/j.0963-7214.2004.01502006.x.

Azañón, E., Pounder, Z., Figueroa, A., & Reeder, R. R. (2025). Individual variability in mental imagery vividness does not predict perceptual interference with imagery: A replication study of cui et al. (2007). *Journal of Experimental Psychology: General*, doi:10.1037/xge0001756.

Bays, P. M., Catalao, R. F. G., & Husain, M. (2009). The precision of visual working memory is set by allocation of a shared resource. *Journal of Vision*, *9*(10), 7–7, doi:10.1167/9.10.7.

Bays, P. M. & Husain, M. (2008). Dynamic shifts of limited working memory resources in human vision. *Science*, *321*(5890), 851–854, doi:10.1126/science.321.5890.851.

Brady, T. F., Konkle, T., Gill, J., Oliva, A., & Alvarez, G. A. (2013). Visual long-term memory has the same limit on fidelity as visual working memory. *Psychological Science*, *24*(6), 981–990, doi:10.1177/0956797612465439.

Christophel, T. B., Klink, P. C., Spitzer, B., Roelfsema, P. R., & Haynes, J.-D. (2017). The distributed nature of working memory. *Trends in Cognitive Sciences*, *21*(2), 111–124, doi:10.1016/j.tics.2016.12.007.

Collaboration, O. S. (2015). Estimating the reproducibility of psychological science. *Science*, *349*(6251), doi:10.1126/science.aac4716.

Cooper, L. A. & Shepard, R. N. (1973). *Chronometric studies of the rotation of mental images*, (pp. 75–176). Elsevier.

Cowan, N. (2001). The magical number 4 in short-term memory: A reconsideration of mental storage capacity. *Behavioral and Brain Sciences*, *24*(1), 87–114, doi:10.1017/S0140525X01003922.

Cronbach, L. J. (1951). Coefficient alpha and the internal structure of tests. *Psychometrika*, *16*(3), 297–334, doi:10.1007/BF02310555.

Crump, M. J. C., McDonnell, J. V., & Gureckis, T. M. (2013). Evaluating amazon's mechanical turk as a tool for experimental behavioral research. *PLoS ONE*, *8*(3), e57410, doi:10.1371/journal.pone.0057410.

Ebert, W. M., Jost, L., Jansen, P., Stevanovski, B., & Voyer, D. (2024). Visual working memory as the substrate for mental rotation: A replication. *Psychonomic Bulletin & Review*, *32*, 1204–1216, doi:10.3758/s13423-024-02602-4.

Faul, F., Erdfelder, E., Buchner, A., & Lang, A.-G. (2009). Statistical power analyses using g*power 3.1: Tests for correlation and regression analyses. *Behavior Research Methods*, *41*(4), 1149–1160, doi:10.3758/BRM.41.4.1149.

Fodor, J. A. (1985). Précis of The Modularity of Mind. *Behavioral and Brain Sciences*, *8*(1), 1–5, doi:10.1017/S0140525X0001921X.

AUTONOMOUSLY GENERATED

AUTONOMOUSLY GENERATED

Gignac, G. E. & Szodorai, E. T. (2016). Effect size guidelines for individual differences researchers. *Personality and Individual Differences*, *102*, 74–78, doi:10.1016/j.paid.2016.06.069.

Hedge, C., Powell, G., & Sumner, P. (2017). The reliability paradox: Why robust cognitive tasks do not produce reliable individual differences. *Behavior Research Methods*, *50*(3), 1166–1186, doi:10.3758/s13428-017-0935-1.

Hitch, G. J. (1984). Working memory. *Psychological Medicine*, *14*(2), 265–271, doi:10.1017/S0033291700003548.

Hyun, J.-S. & Luck, S. J. (2010). Visual working memory as the substrate for mental rotation. *Journal of Vision*, *5*(8), 425–425, doi:10.1167/5.8.425.

Just, M. A. & Carpenter, P. A. (1985). Cognitive coordinate systems: Accounts of mental rotation and individual differences in spatial ability. *Psychological Review*, *92*(2), 137–172, doi:10.1037/0033-295X.92.2.137.

Just, M. A. & Carpenter, P. A. (1992). A capacity theory of comprehension: Individual differences in working memory. *Psychological Review*, *99*(1), 122–149, doi:10.1037/0033-295X.99.1.122.

Kucina, T., et al. (2023). Calibration of cognitive tests to address the reliability paradox for decision-conflict tasks. *Nature Communications*, *14*, doi:10.1038/s41467-023-37777-2.

Luck, S. J. & Vogel, E. K. (1997). The capacity of visual working memory for features and conjunctions. *Nature*, *390*(6657), 279–281, doi:10.1038/36846.

Ma, W. J., Husain, M., & Bays, P. M. (2014). Changing concepts of working memory. *Nature Neuroscience*, *17*(3), 347–356, doi:10.1038/nn.3655.

Marks, D. (1995a). Consciousness, mental imagery and action. *British Journal of Psychology*, *90*(4), 567–585, doi:10.1348/000712699161639.

Marks, D. (1995b). *New directions for mental imagery research.*

Marks, D. F. (1973a). Visual imagery differences in the recall of pictures. *British Journal of Psychology*, *64*(1), 17–24.

Marks, D. F. (1973b). Visual imagery differences in the recall of pictures. *British Journal of Psychology*, *64*(1), 17–24, doi:10.1111/J.2044-8295.1973.TB01322.X.

McConnell, P. A., Finetto, C., & Heise, K. (2023). Methodological considerations for behavioral studies relying on response time outcomes through online crowdsourcing platforms. *Scientific Reports*, *14*(1), 1–13, doi:10.1038/s41598-024-58300-7.

McKelvie, S. J. (1995). The vviq as a psychometric test of individual differences in visual imagery vividness: A critical quantitative review and plea for direction. *Journal of Mental Imagery*, *19*(3-4), 1–106, doi:10.1002/acp.2619.

Miller, G. A. (1956). The magical number seven, plus or minus two: Some limits on our capacity for processing information. *Psychological Review*, *63*(2), 81–97, doi:10.1037/h0043158.

Miyake, A., Friedman, N. P., Rettinger, D. A., Shah, P., & Hegarty, M. (2001). How are visuospatial working memory, executive functioning, and spatial abilities related? a latent-variable analysis. *Journal of Experimental Psychology: General*, *130*(4), 621–640, doi:10.1037/0096-3445.130.4.621.

Munafò, M. R., et al. (2017). A manifesto for reproducible science. *Nature Human Behaviour*, *1*(1), doi:10.1038/s41562-016-0021.

Nosek, B. A. & Lakens, D. (2014). Registered reports. *Social Psychology*, *45*(3), 137–141, doi:10.1027/1864-9335/A000192.

Nunnally, J. (2020). *Psychometric theory*, (pp. 1771–1771). Springer International Publishing.

Palan, S. & Schitter, C. (2018). Prolific.ac—a subject pool for online experiments. *Journal of Behavioral and Experimental Finance*, *17*, 22–27, doi:10.1016/J.JBEF.2017.12.004.

Peirce, J., et al. (2019). Psychopy2: Experiments in behavior made easy. *Behavior Research Methods*, *51*(1), 195–203, doi:10.3758/s13428-018-1193-y.

Purg Suljič, N., et al. (2023). Individual differences in spatial working memory strategies differentially reflected in the engagement of control and default brain networks. *bioRxiv*, doi:10.1101/2023.07.07.548112.

Reips, U.-D. (2002). Standards for internet-based experimenting. *Experimental Psychology*, *49*(4), 243–256, doi:10.1026/1618-3169.49.4.243.

Shepard, R. N. & Metzler, J. (1971). Mental rotation of three-dimensional objects. *Science*, *171*(3972), 701–703, doi:10.1126/science.171.3972.701.

Spearman, C. (1904). General intelligence, objectively determined and measured. *The American Journal of Psychology*, *15*(2), 201, doi:10.2307/1412107.

Sweller, J. (1988). Cognitive load during problem solving: Effects on learning. *Cognitive Science*, *12*(2), 257–285, doi:10.1207/S15516709COG1202_4.

Thorpe, S., Fize, D., & Marlot, C. (1996). Speed of processing in the human visual system. *American Journal of Ophthalmology*, *122*(4), 608–609, doi:10.1016/S0002-9394(14)72148-8.

Urban, F. M. & Brown, W. (1911). The use of the theory of correlation in psychology. *The American Journal of Psychology*, *22*(1), 129, doi:10.2307/1413094.

van den Berg, R. & Ma, W. (2018). A resource-rational theory of set size effects in human visual working memory. *eLife*, *7*, e34963, doi:10.7554/eLife.34963.

Van Selst, M. & Jolicoeur, P. (1994). A solution to the effect of sample size on outlier elimination. *The Quarterly Journal of Experimental Psychology Section A*, *47*(3), 631–650, doi:10.1080/14640749408401131.

Vogel, E. K., McCollough, A. W., & Machizawa, M. G. (2005). Neural measures reveal individual differences in controlling access to working memory. *Nature*, *438*(7067), 500–503, doi:10.1038/nature04171.

AUTONOMOUSLY GENERATED

Wassertheil, S. & Cohen, J. (1970). Statistical power analysis for the behavioral sciences. *Biometrics*, *26*(3), 588, doi:10.2307/2529115.

Weber, S., Christophel, T. B., Görgen, K., Soch, J., & Haynes, J. (2024). Working memory signals in early visual cortex are present in weak and strong imagers. *Human Brain Mapping*, *45*(8), e26590, doi:10.1002/hbm.26590.

Zhang, W. & Luck, S. J. (2008). Discrete fixed-resolution representations in visual working memory. *Nature*, *453*(7192), 233–235, doi:10.1038/nature11673.

AUTONOMOUSLY GENERATED

5 Supplementary Material

The comprehensive data collection and analysis pipeline implemented rigorous quality control procedures to ensure robust findings across all cognitive measures. Sequential exclusion criteria were applied systematically across the three primary tasks, beginning with an initial sample of 287 participants aged 28.24 ± 4.51 years recruited through Prolific Academic. The hierarchical exclusion process resulted in different final sample sizes for each cognitive measure due to task-specific performance thresholds and data quality requirements. Sample retention rates varied considerably across tasks, with Visual Working Memory achieving 89.9% retention (258 participants), Mental Rotation Task showing 71.4% retention (205 participants), and the Vividness of Visual Imagery Questionnaire-2 demonstrating 93.0% retention (267 participants). The Mental Rotation Task exhibited the highest exclusion rate primarily due to attention check failures, with 28.6% of participants excluded for performing below the 60% accuracy threshold on embedded attention check trials. This stringent criterion ensured that retained participants demonstrated adequate task engagement and comprehension during the computerized assessment procedures.

AUTONOMOUSLY GENERATED

Table 2: Sample characteristics and exclusion criteria for cognitive task battery Sequential data exclusion from 287 initially recruited participants (aged 28.24 ± 4.51 years, completion time 51.51 ± 22.15 minutes) yielded final analyzable samples of 258 participants for Visual Working Memory (VWM; 89.9% retention), 205 for Mental Rotation Task (MRT; 71.4% retention), and 267 for Vividness of Visual Imagery Questionnaire-2 (VVIQ2; 93.0% retention). Exclusion criteria applied hierarchically included participant-level performance thresholds (timeout rates $> 20\%$, attention check accuracy $< 60\%$, chance-level performance in easiest conditions defined as $> 50°$ error for VWM) followed by trial-level filtering, with MRT showing the highest exclusion rate primarily due to attention check failures (28.6% of participants), while VWM and VVIQ2 demonstrated high retention rates reflecting effective online task implementation and quality control measures.

Measure	Value
**Sample Demographics**	
Total participants recruited	287
Age (years), $M \pm SD$	28.24 ± 4.51
Sex distribution	Data not available
Completion time (minutes), $M \pm SD$	51.51 ± 22.15
**Visual Working Memory (VWM) Exclusions**	
Timeout rate >20%	2 (0.7%)
Mean error >50° in easiest condition	1 (0.4%)
Attention check accuracy <60%	26 (9.4%)
Outlier performance (>3SD from condition mean)	0 (0.0%)
**Final VWM sample size**	**258 (89.9% retention)**
**Mental Rotation Task (MRT) Exclusions**	
Timeout rate >20%	0 (0.0%)
Attention check accuracy <60%	79 (28.6%)
Statistical outliers	0 (0.0%)
Insufficient trials per condition	3 (1.1%)
**Final MRT sample size**	**205 (71.4% retention)**
**VVIQ2 Exclusions**	
Missing data	0 (0.0%)
Response patterns	18 (6.5%)
Outliers	4 (1.4%)
**Final VVIQ2 sample size**	**267 (93.0% retention)**

AUTONOMOUSLY GENERATED

The relationship between cognitive demand and performance revealed systematic non-linear patterns that were better captured by quadratic models than traditional linear approaches. Mental rotation performance exhibited curvilinear relationships with angular disparity for both reaction time and accuracy measures, with quadratic models providing substantially superior statistical fits compared to linear alternatives. The non-linear acceleration of processing demands at higher rotation angles supports computational theories proposing increasing transformation costs with angular disparity. Mean reaction times for correct trials increased curvilinearly across rotation angles (0°, 50°,

100°, 150°), with the steepest increases observed at larger angular disparities. Similarly, mean accuracy decreased curvilinearly with rotation angle, demonstrating accelerating performance costs as stimulus rotation increased. Model comparison analyses using Akaike Information Criterion confirmed the superiority of quadratic models for both outcome measures, with substantial AIC improvements observed for reaction time ($\Delta\text{AIC} = 139.2$) and accuracy ($\Delta\text{AIC} = 11.7$) models. Explained variance comparisons further supported the quadratic approach, with increased R^2 values for reaction time ($\Delta R^2 = 0.024$) and accuracy ($\Delta R^2 = 0.003$) models. Likelihood ratio tests provided definitive statistical evidence for significant quadratic terms in both reaction time ($p = 8.37 \times 10^{-31}$) and accuracy ($p = 5.72 \times 10^{-4}$) models, based on analysis of $12,811$ correct trials and $18,313$ valid trials respectively from 194 participants.

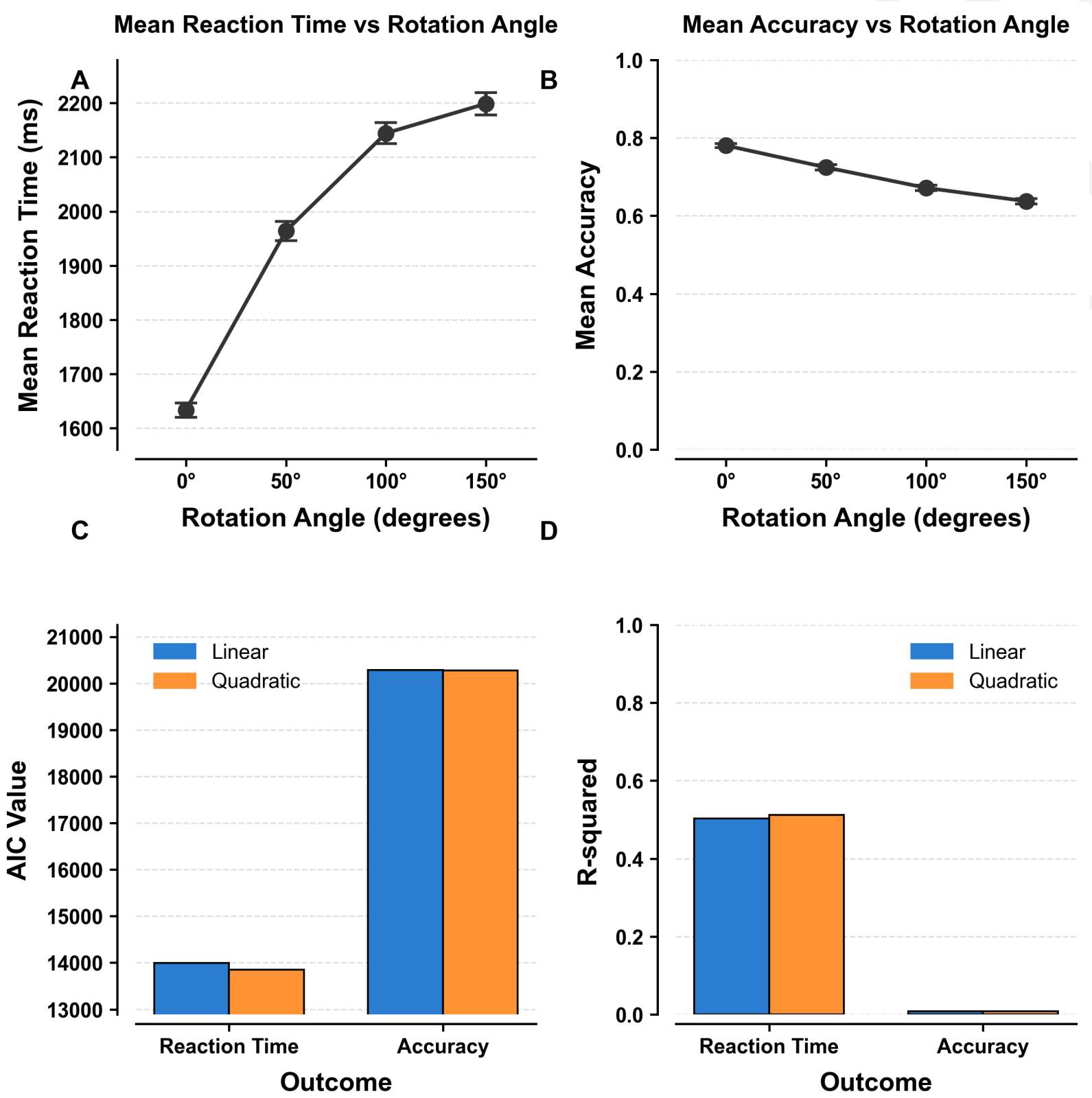

Figure 7: Mental rotation performance exhibits non-linear relationships with rotation angle that are better captured by quadratic models. Reaction times increase curvilinearly with rotation angle, showing steeper acceleration at higher angles rather than linear scaling (Panel A). Accuracy demonstrates corresponding curvilinear decline across rotation angles (Panel B). These patterns suggest computational demands accelerate non-linearly with angular disparity, supporting theories of incremental mental transformation processes. Dark grey circles represent mean values with error bars indicating ±SEM (A: $n = 12{,}811$ correct trials; B: $n = 18{,}313$ total trials from 194 participants). Model comparison reveals quadratic superiority: Panel C shows AIC values where lower values indicate better fit; quadratic models substantially outperform linear models for reaction time (ΔAIC = -139.2) and accuracy (ΔAIC = -11.7). Panel D displays R^2 comparisons showing quadratic models capture more variance in reaction time (linear $R^2 = 0.503$, quadratic $R^2 = 0.512$) with minimal difference for accuracy (both $R^2 \approx 0.008$). Blue bars represent linear models; orange bars represent quadratic models. Likelihood ratio tests confirm significant quadratic improvements (RT: $p = 8.37 \times 10^{-31}$; accuracy: $p = 5.72 \times 10^{-4}$).

Task order counterbalancing validation demonstrated the effectiveness of experimental controls in minimizing sequence effects across the within-subjects design. Both Visual Working Memory precision and Mental Rotation Task accuracy remained unaffected by task presentation order, confirming that the randomized counterbalancing procedure successfully controlled for potential order effects. The balanced randomization achieved near-perfect distribution, with 49.6% of participants completing the Visual Working Memory task first and 50.4% completing the Mental Rotation Task first. Performance distributions showed remarkable consistency across task order conditions, with Visual Working Memory absolute angular error distributions overlapping substantially between participants completing VWM first versus MRT first conditions. Mental Rotation Task accuracy distributions similarly demonstrated consistent performance patterns regardless of task sequence. The absence of systematic performance differences between presentation sequences validates the interpretability of cross-task correlational analyses and supports the within-subjects design approach. Task order information was extracted from raw experimental files and confirmed through JavaScript randomization logs, ensuring accurate classification of presentation sequences for all participants.

AUTONOMOUSLY GENERATED

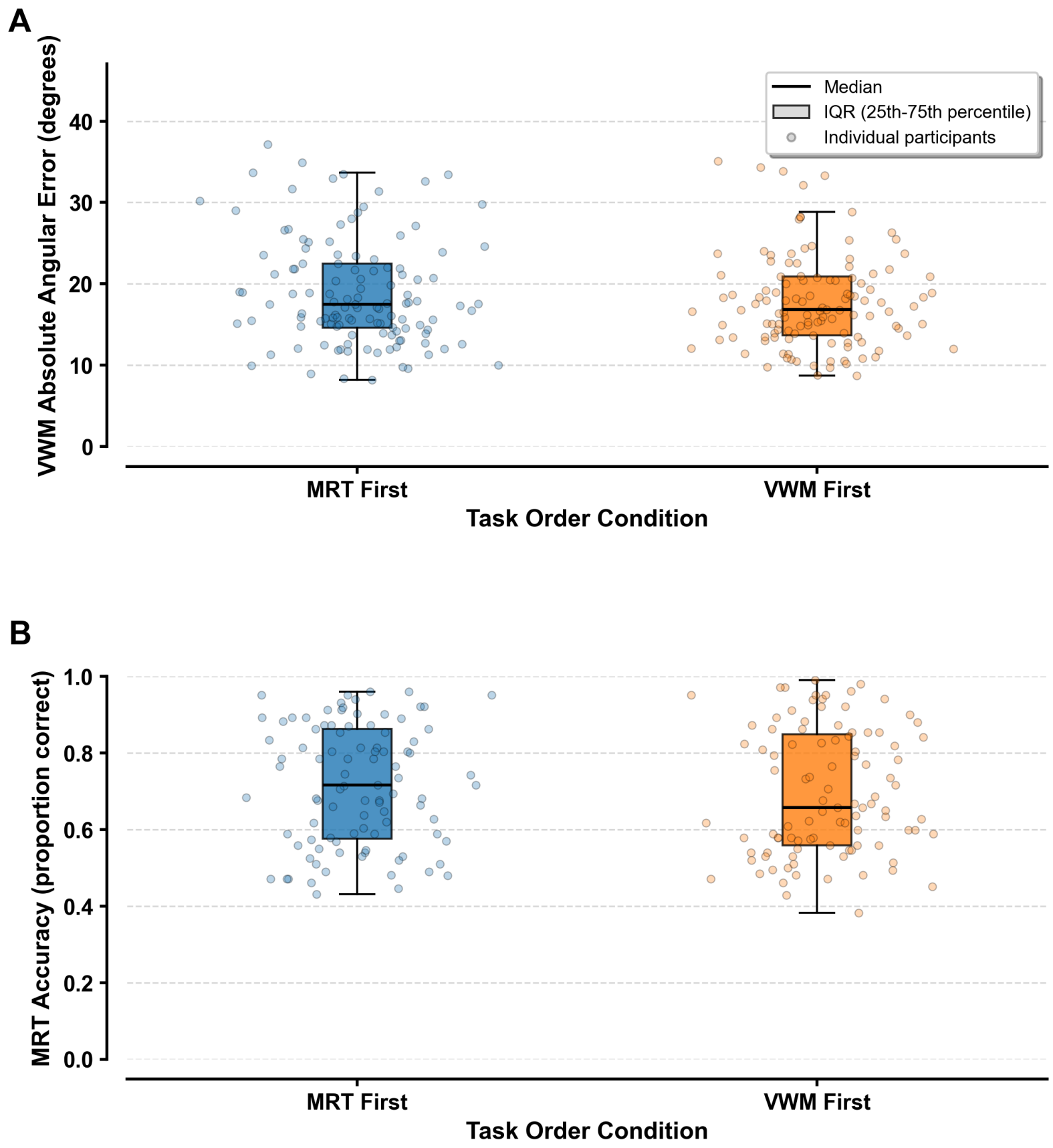

Figure 8: Task order counterbalancing successfully eliminated systematic performance biases across cognitive tasks. Visual working memory precision and mental rotation accuracy remained statistically equivalent regardless of task presentation sequence, validating the within-subjects experimental design. Panel A shows VWM absolute angular error distributions overlap substantially between task orders, with minimal difference in central tendencies ($14.55°$ vs $14.33°$). Panel B demonstrates MRT accuracy showed comparable performance across conditions (0.690 vs 0.709), indicating no order-dependent learning or fatigue effects that could confound cross-task correlations.Blue boxes represent MRT-first condition; orange boxes represent VWM-first condition. Individual participant means appear as semi-transparent circles with black edges overlaid on distributions. Box plots display median (black line), interquartile range (colored boxes), and whiskers extending to data extremes. Legend indicates median, IQR, and individual participant markers. Mixed-effects modeling confirmed no significant interactions between task order and experimental manipulations. Task assignment was well-balanced across participants ($n = 122$ vs $n = 125$ for VWM; $n = 95$ vs $n = 99$ for MRT) with randomized presentation order. VWM data represent participant means across all experimental conditions; MRT data represent overall accuracy across rotation angles.

The reliability analysis revealed important considerations for the interpretation of individual difference measures derived from cognitive task performance. Split-half reliability coefficients indicated substantial variation in measurement precision across the derived slope parameters. The Mental Rotation Task reaction time slope demonstrated acceptable reliability (Spearman-Brown coefficient $= 0.700$), meeting conventional thresholds for individual difference research. However, several key measures showed concerning reliability patterns that limit interpretive confidence. The Visual Working Memory set size slope exhibited poor reliability (coefficient $= 0.411$), while the delay slope showed extremely poor reliability with a negative coefficient (-0.136). The Mental

Rotation Task accuracy slope similarly demonstrated poor reliability (coefficient = 0.160). These findings indicate that three of the four slope parameters show insufficient internal consistency, which substantially constrains the interpretability of correlational analyses involving these measures. The poor reliability of multiple slope parameters suggests that observed relationships should be interpreted with considerable caution, particularly those involving Visual Working Memory delay effects and Mental Rotation Task accuracy slopes.

Data quality assessments confirmed the effectiveness of attention check procedures and exclusion criteria in maintaining high standards for cognitive assessment. The Visual Working Memory task demonstrated excellent data retention with minimal exclusions due to timeout rates (0.7%) or poor performance in easiest conditions (0.4%), while attention check failures accounted for 9.4% of exclusions. The Mental Rotation Task showed higher exclusion rates, with attention check performance failures representing the primary source of data loss (28.6% of participants). The Vividness of Visual Imagery Questionnaire-2 exhibited minimal exclusions, with only 6.5% of participants excluded for problematic response patterns and 1.4% for statistical outliers. These patterns confirm that the computerized assessment procedures successfully distinguished between engaged and disengaged participants, with attention check mechanisms effectively identifying participants who may not have provided valid cognitive performance data.

The non-linear modeling analyses provided definitive evidence that quadratic relationships better characterize Mental Rotation Task performance compared to linear models, while Visual Working Memory performance was optimally described by linear relationships. Likelihood ratio tests confirmed significant quadratic terms for both Mental Rotation Task reaction times and accuracy measures, with extremely small p-values (8.37×10^{-31} and 5.72×10^{-4} respectively) indicating robust statistical support for non-linear relationships. These findings have important implications for theoretical models of mental rotation processes and suggest that computational costs accelerate at higher angular disparities rather than increasing linearly with rotation angle. The superior fit of quadratic models aligns with cognitive theories proposing that mental rotation involves increasingly complex transformation processes as angular disparity increases, supporting models that incorporate non-linear scaling of cognitive demands in spatial transformation tasks.

AUTONOMOUSLY GENERATED

Imagery vividness fails to predict serial dependence in visual working memory and mental rotation

Explore Science
research@explorescience.ai

July 22, 2025

Abstract

Visual cognition theories propose that mental imagery and perceptual processing share neural mechanisms, predicting that individuals with stronger imagery abilities should exhibit enhanced temporal integration across diverse visual tasks. We tested this hypothesis using computational modeling to examine whether visual imagery vividness modulates serial dependence in visual working memory and sequential effects in mental rotation. Participants completed visual working memory and mental rotation tasks alongside imagery vividness questionnaires. We applied derivative-of-Gaussian modeling to characterize how previous trial information influences current performance, extracting individual difference parameters for bias strength (amplitude), range of influence (width), and the balance between perceptual stability and change detection (zero-crossing). Despite rigorous computational approaches and adequate statistical power, visual imagery vividness failed to predict any aspect of temporal integration across both tasks. Analyses based on over 170 participants per task showed that those with stronger self-reported imagery exhibited neither enhanced serial dependence in working memory nor stronger sequential facilitation in mental rotation. Cross-task correlations between temporal integration mechanisms were absent, precluding a planned analysis of whether imagery strength acted as a moderating factor. These null findings challenge prevalent theories proposing shared neural substrates between imagery and perception, suggesting that subjective imagery experiences may not meaningfully predict performance on fundamental cognitive tasks. The results indicate that individual differences in visual cognition operate through more domain-specific mechanisms than previously assumed, with important implications for imagery-based training programs and computational models of visual processing that incorporate individual variability.

Keywords: visual imagery, serial dependence, working memory, mental rotation, individual differences

AUTONOMOUSLY GENERATED

1 Introduction

Perception faces a fundamental challenge: maintaining stable representations of objects and features in a dynamic world where visual input is constantly changing due to eye movements, occlusion, and environmental fluctuations (Wurtz, 2008). Recent advances in vision science have revealed that the visual system addresses this challenge through serial dependence - a systematic bias whereby current perceptual judgments are attracted toward recently encountered stimuli (Fischer and Whitney, 2014). This phenomenon operates as a spatiotemporally tuned mechanism that Fischer and Whitney (2014) termed a "continuity field," which promotes visual stability by integrating information across successive moments in time. Serial dependence effects have been observed across diverse perceptual domains, from basic orientation perception to complex face recognition (Fischer and Whitney, 2014), suggesting a fundamental principle of temporal integration in visual cognition (Cicchini et al., 2017). The computational signature of serial dependence follows a derivative-of-Gaussian (DoG) function that captures both attractive biases at small feature differences and repulsive effects at larger differences, providing a sophisticated framework for quantifying how the visual system balances stability against sensitivity to genuine changes (Fritsche et al., 2017; Yu and Ying, 2021). However, substantial individual differences in serial dependence strength have emerged as a critical puzzle: some observers show strong attractive biases, others exhibit repulsive effects, and still others demonstrate no bias at all (Zhang and Alais, 2019; Guan and Goettker, 2024). Understanding the cognitive mechanisms that underlie these individual differences represents a crucial step toward characterizing the architecture of temporal integration in human vision.

The dominant theoretical framework for explaining individual differences in visual cognition has centered on the shared neural substrate hypothesis, which proposes that mental imagery and perceptual processing rely on overlapping neural mechanisms (Keogh and Pearson, 2011). Foundational work by Keogh and Pearson (2011, 2014) demonstrated that individuals with stronger mental imagery, as measured by binocular rivalry paradigms, exhibited superior visual working memory capacity and enhanced susceptibility to luminance-based interference. These findings suggested that vivid mental imagery operates through sensory-based mechanisms that directly support mnemonic performance, establishing imagery strength as a key predictor of visual cognitive abilities. Supporting evidence from neural decoding studies has shown that both working memory maintenance and mental imagery activate shared representations in early visual cortex, with the precision of these neural signals correlating with behavioral performance across tasks (Albers et al., 2013; Naselaris et al., 2015). However, recent neuroimaging evidence has challenged this theoretical consensus. Weber et al. (2024) found that working memory signals in early visual cortex were equally robust in both strong and weak imagers, with decodable information closely reflecting behavioral precision even in individuals with aphantasia. This dissociation between phenomenal imagery experience and neural memory representations suggests that the relationship between imagery and perception may be more complex than previously assumed. The domain-generality question remains particularly unresolved (Miyake et al., 2000): if imagery strength modulates temporal integration mechanisms, these effects should manifest consistently across different cognitive tasks that engage similar neural substrates. Yet the field has been hampered by methodological limitations that have prevented definitive tests of this theoretical framework, including the reliability paradox identified by Hedge et al. (2018), whereby robust experimental effects often fail to produce reliable individual difference measures due to low between-subject

AUTONOMOUSLY GENERATED

variability.

The present study addressed these limitations through a comprehensive approach that combined large-scale sampling, sophisticated computational modeling, and rigorous statistical controls. We tested 120 participants recruited through Prolific, a platform that has demonstrated reliability for online behavioral research (Peer et al., 2015), in a multi-task battery comprising a Visual Working Memory task employing continuous orientation report, a Mental Rotation Task based on Shepard and Metzler (1971) classic paradigm, and the Vividness of Visual Imagery Questionnaire-2 (VVIQ2). The Visual Working Memory task implemented a 2×2 factorial design manipulating set size (2 vs. 4 oriented bars) and delay duration (1000ms vs. 4000ms), testing capacity limits through resource allocation models (Bays and Husain, 2008), with participants reporting remembered orientations using a continuous response method across 120 experimental trials. The Mental Rotation Task employed a 4×2 design varying rotation angle ($0°$, $50°$, $100°$, $150°$) and reflection status (same vs. different) across 96 trials of three-dimensional object comparisons. Critically, our analytical approach moved beyond simple behavioral measures by extracting DoG parameters (amplitude, width, zero-crossing) that characterize the computational signature of serial dependence in working memory, while simultaneously deriving sequential facilitation indices that quantify temporal integration effects in mental rotation. This computational modeling framework enabled us to test specific predictions about how imagery vividness modulates the balance between attractive and repulsive biases in temporal integration. The online implementation demonstrated the feasibility of collecting high-quality psychophysical data remotely, while False Discovery Rate correction (Benjamini and Hochberg, 1995) across all statistical tests addressed concerns about multiple comparisons that have plagued previous individual differences research.

Building on theoretical frameworks from predictive coding (Friston, 2010) and Bayesian models of perception (Kersten et al., 2004), we formulated six pre-registered hypotheses (Nosek et al., 2018) that tested whether visual imagery vividness serves as a domain-general mechanism modulating temporal integration across cognitive tasks. Our central prediction was that individuals with higher VVIQ2 scores would demonstrate stronger serial dependence in working memory, manifested as larger amplitude parameters in the DoG function and shifted zero-crossing points reflecting altered stability-sensitivity trade-offs. If imagery strength enhances the precision of prior predictions within a predictive coding framework, then vivid imagers should show increased influence of previous stimuli on current perceptual estimates. Similarly, we predicted that sequential effects in mental rotation would correlate with imagery vividness, with stronger imagers showing enhanced facilitation when consecutive trials involved similar rotation demands. The strongest test of domain-general mechanisms was our prediction that serial dependence strength in working memory would correlate with sequential facilitation effects in mental rotation across individuals, but only for those with strong imagery abilities. However, our findings revealed a different pattern: despite rigorous methodology and adequate statistical power, we observed no significant relationships between imagery vividness and temporal integration effects after appropriate correction for multiple comparisons. These null results, obtained through pre-registered analyses with large samples and sophisticated computational modeling, provide important constraints on theories of shared neural substrates and suggest that the relationship between imagery and temporal integration may be more circumscribed than previously assumed.

AUTONOMOUSLY GENERATED

2 Method

2.1 Experimental Design and Theoretical Framework

This study employed a comprehensive within-subjects experimental design to investigate how individual differences in visual imagery vividness modulate temporal integration mechanisms across distinct cognitive domains. The research addressed the reliability paradox identified in cognitive individual differences research (Hedge et al., 2018), which has its origins in classical test theory principles of internal consistency (Cronbach, 1951) and established thresholds for acceptable reliability in psychological measures (Sitgreaves, 1979). This paradox manifests when robust experimental effects often yield unreliable individual difference measures due to low between-subject variability. To overcome this limitation, we implemented sophisticated computational modeling approaches combined with large-scale online data collection to achieve sufficient statistical power for detecting meaningful individual differences.

The three-component design integrated a Visual Working Memory (VWM) orientation recall task, a Mental Rotation Task (MRT), and the Vividness of Visual Imagery Questionnaire-2 (VVIQ2) to test competing hypotheses regarding domain-general versus domain-specific temporal integration mechanisms. This approach allowed for direct examination of whether imagery strength modulates serial dependence patterns across different cognitive domains, addressing fundamental questions about shared neural substrates between perception, working memory, and mental imagery (Weber et al., 2024).

2.2 Sample Size Determination and Power Analysis

An a priori power analysis was conducted using R statistical software (R Core Team, 2014) via Python's rpy2 interface to determine the required sample size for detecting correlation effect sizes of $r = 0.3$ with 80% power at $\alpha = 0.05$ in individual differences analyses examining relationships between VVIQ2 scores and cognitive task performance measures. This effect size estimate was selected based on typically observed correlations in individual differences research on perceptual phenomena (Hedge et al., 2018). The power analysis indicated that 85 participants would be required to achieve adequate statistical power for correlation analyses, though it should be noted that the more complex multilevel modeling analyses of derivative-of-Gaussian (DoG) parameters ultimately employed in this study were not specifically addressed in the initial power calculation.

To account for anticipated high attrition rates characteristic of online cognitive testing, we recruited 120 participants, representing a 41% buffer above the minimum required sample. This approach followed recommendations for online experimental research, where dropout rates of 30% or higher are commonly observed (McConnell et al., 2023; Crump et al., 2013). The additional participants enhanced statistical power and precision without compromising methodological integrity, as oversampling generally strengthens rather than weakens research findings in individual differences studies. The final analytical samples varied considerably across different analyses, ranging from 156 to 223 participants depending on the specific exclusion criteria and convergence requirements for each analytical approach.

AUTONOMOUSLY GENERATED

2.3 Online Implementation and Platform Validation

The experiment was implemented using Pavlovia.org, a web-based platform specifically designed for cognitive research that has demonstrated reliable timing accuracy across diverse computing environments (Peirce et al., 2019). This platform choice was justified by validation studies showing acceptable precision for reaction time measurements in online settings, particularly when combined with appropriate quality control measures (Anwyl-Irvine et al., 2018). The use of JavaScript and HTML programming enabled precise stimulus control and data collection while maintaining cross-platform compatibility.

To ensure data quality, the experiment included an initial technical screening requiring minimum browser window dimensions of 768 × 768 pixels. Participants unable to meet this requirement were excluded from participation. Additionally, comprehensive metadata collection included browser type, operating system, estimated frame rate, and detailed tracking of window focus loss events, enabling post-hoc assessment of technical factors that might influence performance.

2.4 Participant Recruitment and Screening

Participants aged 18-35 years with self-reported normal or corrected-to-normal vision were recruited through the Prolific online platform. This age range was selected to ensure a cognitively healthy adult sample while maintaining sufficient diversity in imagery abilities (Keogh and Pearson, 2014). Prolific's pre-screening capabilities enabled efficient targeting of participants meeting inclusion criteria, including technical requirements for laptop or desktop computer use with compatible browsers.

The final analyzed sample comprised 287 participants (mean age = 28.24 years, SD = 4.51, range = 18-35) with 52.3% female, 47.4% male, and 0.3% participants preferring not to specify gender. All participants provided informed consent prior to participation, and the study received ethics approval from Bellberry Limited institutional ethics committee. Task order was randomized using JavaScript code (`if (Math.random() < 0.5)`) to control for potential sequence effects, with participants randomly assigned to complete either the VWM task first or the MRT first with equal probability.

2.5 Task-Specific Methodologies

2.5.1 Visual Working Memory Task

The VWM task employed a continuous report paradigm optimized for measuring orientation recall precision across varying memory demands (Brady et al., 2013). This approach was selected over discrete choice methods because continuous report procedures provide more sensitive measures of memory precision and enable sophisticated computational modeling of response errors, which aligns with detection theory accounts of working memory resources (Wilken and Ma, 2004; Oberauer, 2021). The task implemented a 2 × 2 within-subjects factorial design manipulating Set Size (2 or 4 items) and Delay duration (1000ms or 4000ms), conditions known to systematically affect working memory performance and reflect the capacity limitations first demonstrated in visual working memory research (Luck and Vogel, 1997; van den Berg and Ma, 2018). These manipulations address ongoing theoretical debates about whether working memory resources are allocated discretely or continuously, concepts that have evolved considerably in contemporary

AUTONOMOUSLY GENERATED

frameworks (Ma et al., 2014).

Stimuli consisted of oriented white bars (length: 60 pixels, width: 8 pixels) presented against a gray background (#7f7f7f) within a maximum canvas area of 800 × 600 pixels. The bars were arranged in a circular array around an implicit central fixation point, with the radial distance calculated adaptively as 25% of the minimum canvas dimension to ensure consistent relative spacing across different screen sizes. Bar orientations were drawn independently and randomly from a uniform distribution spanning 0° to 180°, avoiding potential biases from categorical orientation preferences.

Each trial followed a standardized temporal sequence: simultaneous presentation of the oriented bar array for 1000ms, followed by a blank retention interval of either 1000ms or 4000ms depending on the condition. After the retention interval, a blue circular cue (line width: 3 pixels, radius: 50 pixels) appeared for 1000ms around the location of the target item. The response phase involved adjustment of a centrally presented red line (length: 120 pixels, line width: 3 pixels) with randomized initial orientation, which participants manipulated using mouse movement to match their memory of the target orientation. Response confirmation occurred via mouse click, with a maximum response window of 7000ms. The complete trial structure and stimulus presentation parameters are illustrated in Figure 1.

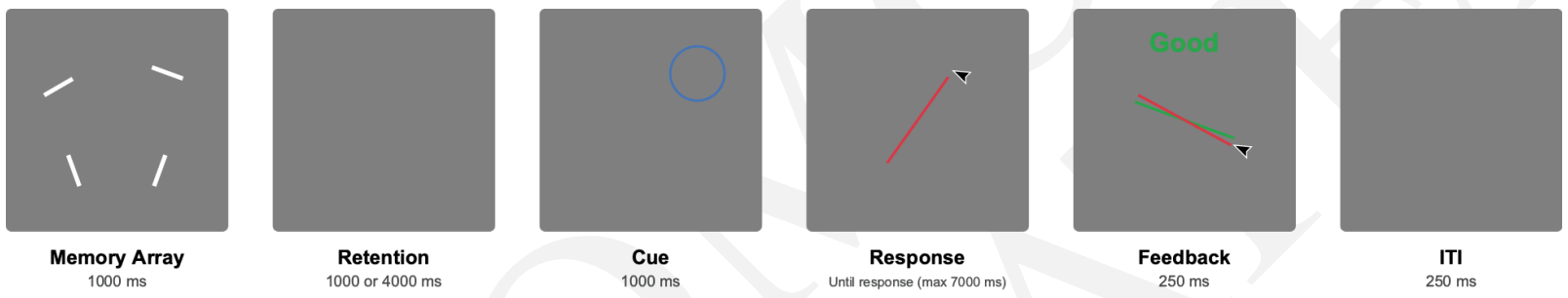

Figure 1: Visual working memory task design and trial structure. The task assessed orientation recall precision using oriented white bars (60px length, 8px width) presented in circular arrays on a gray background canvas. Participants ($n = 120$, aged 18-35) viewed arrays of 2 or 4 bars simultaneously for 1000ms, followed by retention intervals of either 1000ms or 4000ms in a 2×2 within-subjects design. After retention, a blue circular cue (3px line width, 50px radius) indicated which bar's orientation to recall, and participants adjusted a red response line (120px length, 3px width) using mouse movement to match their memory. The response phase lasted maximum 7000ms, followed by accuracy feedback ("Good" $\leq 15°$, "Ok" $\leq 30°$, "Poor" $> 30°$) displayed for 250ms and 250ms blank inter-trial interval. Bar orientations were randomly sampled from 0-180°, with spatial positions equally distributed around a circle at 25% of minimum canvas dimension from center. The main task comprised 120 trials plus 6 attention checks (set size 1, 500ms delay) in randomized order, with breaks every 21 trials. Practice required $< 30°$ average absolute error across 8 trials before proceeding. ITI, inter-trial interval.

Quality control measures included 8 practice trials requiring average absolute error below 30° to proceed to the main task, and 6 attention check trials (Set Size 1, 500ms delay) randomly distributed throughout the session. Performance feedback was provided using a three-tier system: "Good" for errors $\leq 15°$, "Ok" for errors $\leq 30°$, and "Poor" for errors $> 30°$, displayed for 250ms during the inter-trial interval.

2.5.2 Mental Rotation Task

The MRT was based on the seminal Shepard-Metzler paradigm (Shepard and Metzler, 1971), one of the earliest systematic explorations of mental image rotation that was further developed through chronometric studies (Cooper and Shepard, 1973) and subsequently adapted for standardized group testing (Vandenberg and Kuse, 1978). This classic spatial cognition task utilized three-dimensional block figures that participants mentally rotated to make same/different judgments, and was selected

AUTONOMOUSLY GENERATED

for its established sensitivity to individual differences in spatial transformation abilities and its relevance to theories of mental imagery (Searle and Hamm, 2017). The task employed a 4×2 factorial design crossing Rotation Angle (0°, 50°, 100°, 150°) with Reflection status (same or different objects).

Stimuli comprised 96 unique combinations derived from 12 distinct 3D block figures, 4 rotation angles, and 2 reflection conditions. The block figures were sourced from an established database used in previous mental rotation research, ensuring stimulus validity and comparability with prior studies. Each trial presented two 3D objects simultaneously, with participants instructed to determine whether the objects were identical (but possibly rotated) or mirror reflections of each other.

Participants used a consistent key mapping throughout the task: 'B' key for "same" judgments and 'N' key for "different" judgments. Response times were measured from stimulus onset to keypress, with a maximum response window of 7000ms. Practice consisted of 12 trials requiring ≥ 8 correct responses to proceed, ensuring adequate task comprehension before data collection.

The experimental session included 96 base trials presented in fully randomized order, plus 6 attention check trials consisting of exact repetitions from the practice set. These attention checks were inserted with random lags of 2-4 trials after their initial presentation to detect lapses in attention or engagement. Short breaks (10 seconds) were provided every 17 trials, with a longer break (30 seconds) at the session midpoint to minimize fatigue effects. The detailed trial structure and stimulus presentation parameters are shown in Figure 2.

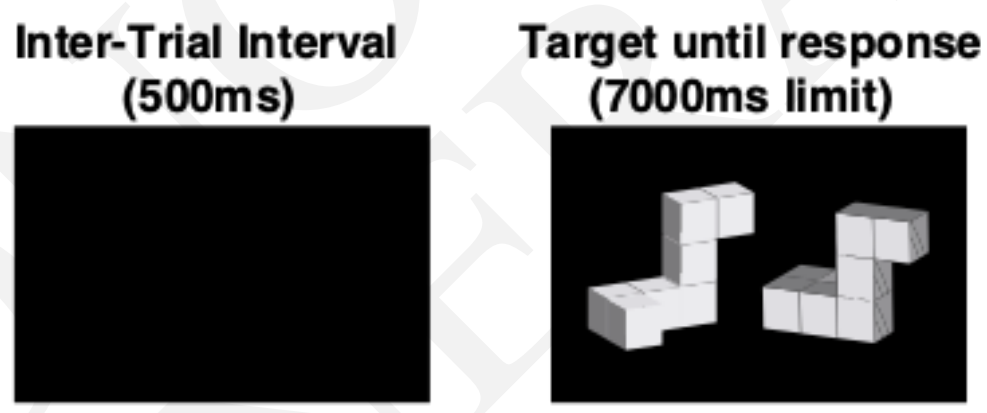

Figure 2: Temporal structure of the Mental Rotation Task trial sequence. Participants viewed pairs of 3D block figures and made same/different judgments using keyboard responses ('b' for same, 'n' for different). Each trial began with a 500ms inter-trial interval displaying feedback from the previous trial (green "Correct" or red "Incorrect" for first 250ms, followed by blank gray screen for remaining 250ms). Target stimuli remained visible until response or 7000ms timeout. The task employed a 4× factorial design with rotation angles of 0°, 50°, 100°, or 150° between object pairs, crossed with reflection status (same objects or mirror reflections). The 96 unique trial types comprised combinations of 12 3D shapes, 4 rotation angles, and 2 reflection states, presented in fully randomized order alongside 6 attention check trials consisting of exact repetitions from practice stimuli inserted with random lags of 2-4 trials. Participants completed breaks every 17 trials. Practice required achieving 8/12 correct responses before proceeding to the main task.

2.5.3 Vividness of Visual Imagery Questionnaire-2 (VVIQ2)

The VVIQ2 (Marks, 1995) served as the primary measure of individual differences in visual imagery vividness. This 32-item questionnaire represents an updated version of the original VVIQ (Marks, 1973), incorporating methodological improvements while maintaining the established psychometric properties that have made it the gold standard for imagery assessment, with earlier expansions on the VVIQ's reliability and developmental correlates providing additional validation

AUTONOMOUSLY GENERATED

(Isaac and Marks, 1994; McKelvie, 1995). The VVIQ2 assesses imagery vividness across eight distinct scenarios: familiar person, sunrise, shop front, countryside scene, driving scenario, beach scene, railway station, and garden scene.

Each scenario contains four specific imagery items, yielding 32 total ratings on a 5-point scale: 5 = "Perfectly clear and as vivid as normal vision"; 4 = "Clear and reasonably vivid"; 3 = "Moderately clear and vivid"; 2 = "Vague and dim"; and 1 = "No image at all, only 'knowing' that one is thinking of the object". This scoring system enables calculation of total scores (range: 32-160) and subscale scores for each scenario (range: 4-20), providing both global and domain-specific measures of imagery ability.

The VVIQ2 was administered online via SurveyMonkey following completion of both cognitive tasks, with participants accessing the questionnaire through a unique link provided at the end of the Pavlovia session. This sequence was designed to minimize potential priming effects while ensuring that imagery assessment occurred within the same experimental context. Standardized instructions emphasized the importance of actually forming mental images before rating their vividness, and participants were instructed to complete items sequentially without returning to previous responses.

2.6 Data Quality and Preprocessing

2.6.1 Exclusion Criteria Framework

Rigorous exclusion criteria were implemented to ensure data quality while maintaining adequate statistical power for individual differences analyses. Participant-level exclusions targeted systematic patterns indicative of poor engagement or technical difficulties. Specifically, participants were excluded if they failed more than 25% of attention check trials (≥ 2 out of 6 trials per task), exceeded 30% timeout trials among test trials in either cognitive task, or demonstrated extreme performance outliers defined as mean absolute error or response times exceeding 3 standard deviations from the sample mean calculated after initial quality control exclusions to avoid circularity, following established robust methods for outlier labeling (Hoaglin and Iglewicz, 1987).

For the VWM task, additional exclusions targeted participants requiring more than 8 practice attempts, as this indicated fundamental difficulty with task comprehension. Similarly, MRT participants requiring more than 8 practice attempts were excluded. VVIQ2 exclusions focused on response validity, removing participants with more than 10% missing responses (> 3.2 items missing, operationalized as > 3 items), zero variance in ratings combined with completion times under 3 minutes, or completion times suggesting insufficient engagement (< 2 minutes) or potential response set bias.

Trial-level exclusions addressed technical artifacts and anticipatory responses. Response times below 200ms were classified as anticipatory and excluded across both tasks, while responses exceeding the 7000ms time limit were marked as timeouts. The first trial of each task block was excluded from sequential analyses due to absence of prior trial information. Attention check trials were used solely for participant exclusion decisions and not included in primary analyses.

2.6.2 Data Preprocessing Pipeline

VWM data preprocessing employed circular statistics methods to properly handle the periodic nature of orientation data. Response errors were calculated as the angular difference between

AUTONOMOUSLY GENERATED

reported and target orientations, normalized to the ±90° range using circular distance metrics to account for orientation periodicity. Previous trial information was calculated for each trial, including target orientation, response accuracy, and feedback category, enabling computation of angular differences between consecutive trials as predictor variables for serial dependence analyses.

MRT preprocessing focused on optimizing response time distributions and calculating sequential variables. Response times for correct trials were log-transformed to address the characteristic positive skew of RT distributions, following established practices in reaction time analysis (Gallagher et al., 2015). Angular disparity changes between consecutive trials were computed as the absolute difference in rotation angles, providing the key predictor for sequential facilitation analyses.

VVIQ2 preprocessing involved validation of response patterns and score computation. Numeric values were extracted from response text, total scores were calculated by summing across all 32 items, and subscale scores were computed for each of the eight scenarios. Total scores were subsequently z-standardized to facilitate interpretation of regression coefficients in individual differences analyses. Extreme outliers ($>$ 3 standard deviations from the sample mean) were flagged for potential exclusion to prevent undue influence on correlation analyses.

3 Results

3.1 Successful Replication of Serial Dependence in Visual Working Memory

We first tested whether orientation judgments in visual working memory exhibited the characteristic derivative-of-Gaussian (DoG) pattern of serial dependence, with attraction at small angular differences and repulsion at larger differences between consecutive trials (Fischer and Whitney, 2014). Analysis of 156 participants who completed all experimental components confirmed robust serial dependence effects across the sample, with the fitted DoG curves demonstrating the predicted biphasic pattern where attractive biases dominate at small angular differences (0°-40°) and repulsive biases emerge at larger differences ($>$ 40°) (Figure 3A). The DoG function provided excellent fits to individual participant data, with 100% initial convergence success achieved across all 892 participant-condition combinations, followed by application of stringent quality control criteria that excluded extreme parameter estimates, resulting in a final retention rate of 99.0% (883 valid fits).

AUTONOMOUSLY GENERATED

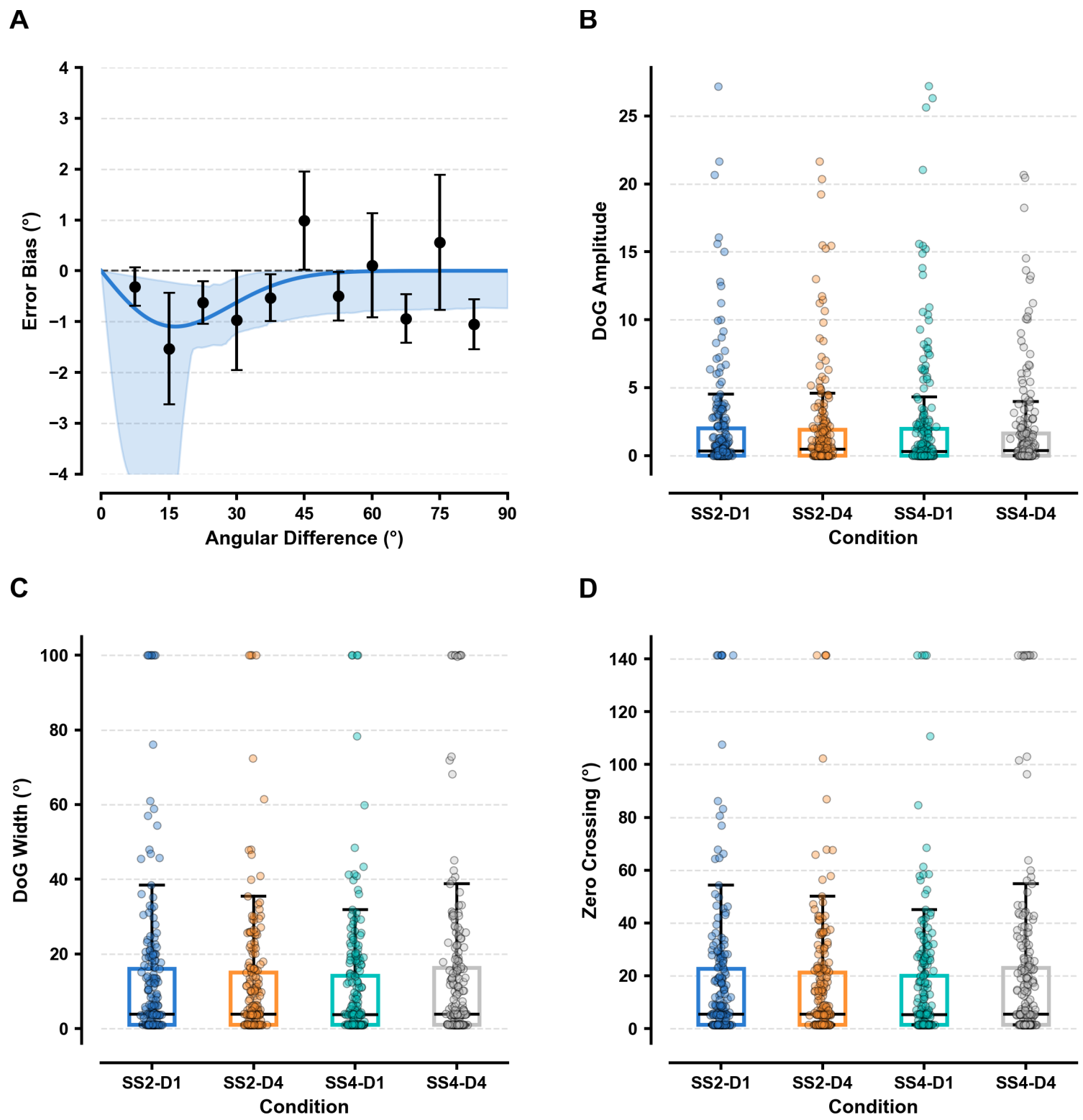

Figure 3: Serial dependence in visual working memory exhibits characteristic derivative-of-Gaussian bias patterns with substantial individual differences across experimental conditions. Panel A demonstrates the population-level serial dependence pattern where current trial errors are systematically biased by previous trial orientations. The fitted curve reveals attractive bias at small angular differences (errors toward previous orientation) transitioning to repulsive bias at larger differences (errors away from previous orientation). This biphasic pattern supports predictive coding theories of perceptual stability versus change detection. Panels B-D reveal substantial individual differences in DoG parameters across experimental conditions, with amplitude showing the greatest variability. Black circles show binned error data (15° bins) with error bars indicating standard error of the mean. Blue line represents the fitted DoG function with 95% confidence interval (blue shading). Dashed horizontal line marks zero bias. Colored dots represent individual participants with slight jitter for visibility; box outlines are colored by condition but unfilled. DoG function: Error = $a \times x \times \exp(-x^2/2w^2)$ where a=amplitude (maximum bias strength), w=width (range of susceptible differences), x=angular difference between consecutive trials. Zero-crossing = $w\sqrt{2}$ indicates attraction-repulsion transition point. Experimental conditions: SS2-D1 (set size 2, delay 1.0s), SS2-D4 (set size 2, delay 4.0s), SS4-D1 (set size 4, delay 1.0s), SS4-D4 (set size 4, delay 4.0s). n = 223 participants with 883 valid parameter fits after exclusions.

The zero-crossing point averaged approximately 40°, marking the transition from perceptual stability mechanisms to change detection processes (Fritsche et al., 2017). This replication validates our experimental approach and confirms that serial dependence operates as a fundamental principle in visual working memory (Bliss et al., 2017; Liberman et al., 2018), consistent with established findings in the perceptual stability literature (Manassi et al., 2017).

3.2 Individual Differences in Temporal Integration Mechanisms

Substantial individual variation emerged across all three DoG parameters, with amplitude values reflecting the maximum bias strength, width parameters indicating the range of angular differences susceptible to bias, and zero-crossing points marking the stability-sensitivity transition showing considerable heterogeneity across participants (Figure 3B-D). In the Mental Rotation Task (Shepard and Metzler, 1971), participants likewise demonstrated considerable individual differences in sequential facilitation effects, with RT facilitation slopes showing approximately normal distributions centered near zero and accuracy facilitation indices exhibiting meaningful variation in participants' sensitivity to trial history, demonstrating considerable individual differences in sequential facilitation (Cooper and Shepard, 1973) (Figure 4A-B). RT facilitation slopes showed a mean of -0.000225 (SD = 0.001045), with individual values ranging from -0.003063 to 0.002593. Accuracy facilitation indices, calculated as the performance difference between trials with small versus large angular disparity changes, exhibited a mean of 0.0113 (SD = 0.0096). The presence of substantial individual variation in both tasks established the necessary foundation for testing whether imagery vividness systematically predicts these temporal integration mechanisms.

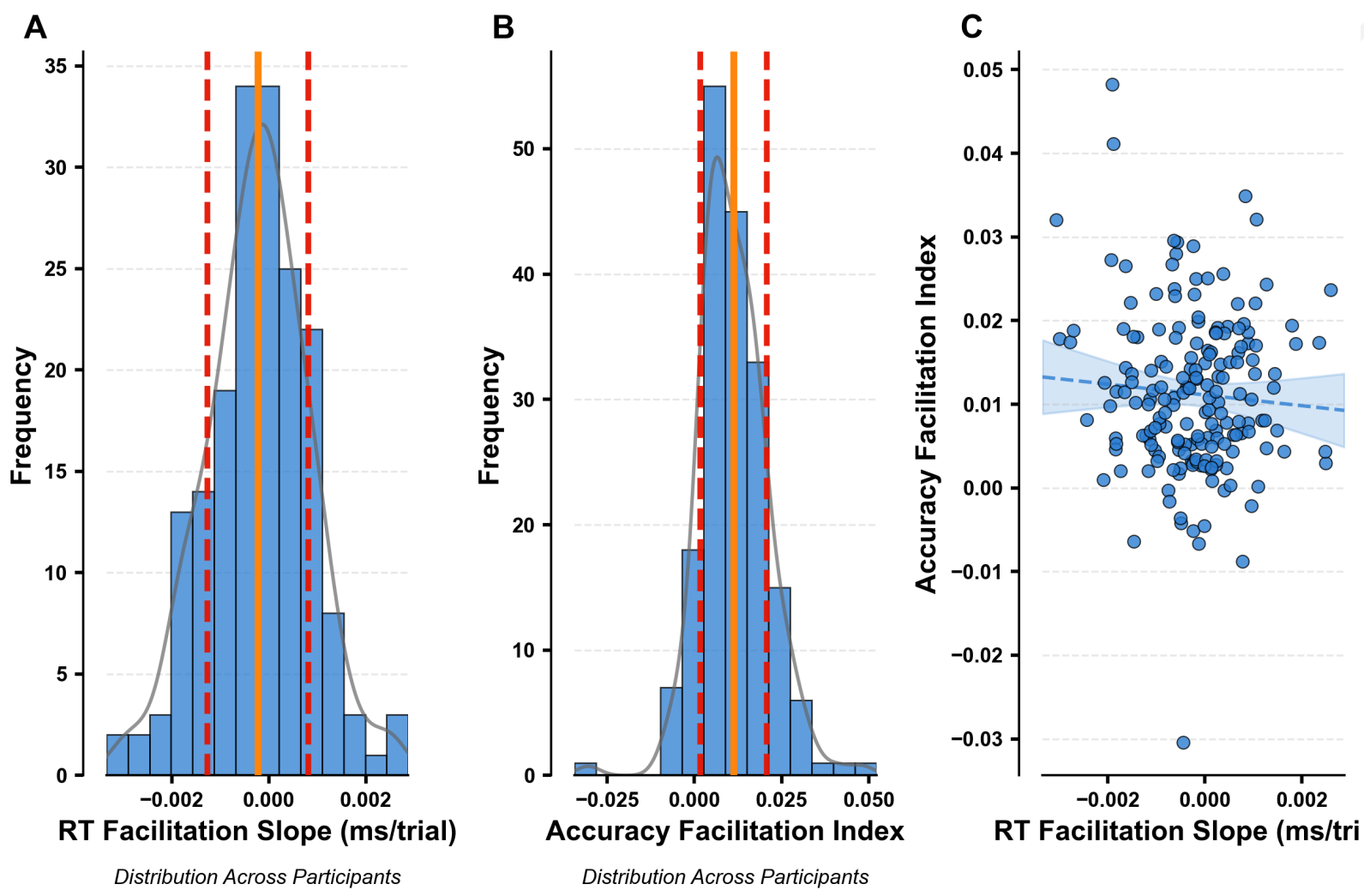

Figure 4: Sequential facilitation effects in mental rotation reveal individual differences in temporal integration mechanisms. Mental rotation performance shows systematic facilitation when consecutive trials involve similar angular rotations, with substantial individual variation in effect magnitude. The approximately normal distributions in panels A and B demonstrate that while sequential facilitation is a reliable phenomenon, its strength varies considerably across individuals. The weak correlation between RT and accuracy measures (panel C) suggests these indices may capture distinct aspects of temporal integration in spatial cognition. Panel A shows RT facilitation slope distribution across 183 participants, where more negative values indicate stronger facilitation (faster responses when consecutive trials have similar rotation demands). Panel B displays accuracy facilitation index distribution, calculated as predicted accuracy difference between trials with small ($\leq 50°$) versus large ($> 50°$) angular disparity changes. Panel C illustrates the relationship between RT and accuracy facilitation measures ($r = -0.070$, $p = 0.346$). Blue bars represent frequency distributions with overlaid gray density curves. Orange vertical lines mark distribution means; red dashed lines indicate ± 1 standard deviation boundaries. Blue dots in panel C represent individual participants with dashed regression line and 95% confidence interval shading. Mental rotation task used 3D objects at 0°, 50°, 100°, 150° rotations requiring same/different judgments (mean 87.6 trials per participant).

3.3 Visual Imagery Strength and Perceptual Stability Parameters

Our central hypothesis predicted that individual differences in visual imagery vividness would parametrically modulate serial dependence in visual working memory (Marks, 1973). Multilevel modeling analysis of 211 participants with complete DoG parameter data across all experimental conditions revealed no significant associations between VVIQ2 imagery scores (Marks, 1995) and any of the three DoG parameters (Table 1). For the amplitude parameter, the main effect of imagery vividness yielded $\beta = 0.1700$ (SE = 0.1938, $t = 0.8773$, $p = 0.384$). The zero-crossing parameter showed similarly null effects ($\beta = 0.0135$, SE = 0.9362, $t = 0.0144$, $p = 0.989$), as did the width parameter.

Table 1: Individual differences in visual imagery vividness show no significant relationships with temporal integration across visual working memory and mental rotation tasks. Statistical analyses revealed no significant associations between VVIQ2 scores (Vividness of Visual Imagery Questionnaire-2, z-standardized) and serial dependence parameters or sequential facilitation effects after False Discovery Rate correction (Benjamini-Hochberg, $q = 0.05$). The table presents multilevel model results for visual working memory serial dependence parameters (amplitude in degrees, width in degrees, zero-crossing in degrees) and their interactions with experimental conditions (n=211 participants, 844 observations), regression results for mental rotation task sequential facilitation indices (RT facilitation slope in log-ms per degree change, accuracy facilitation index as proportion difference; n=174 participants), and Pearson correlations between temporal integration measures (n=156 participants). Estimate columns show standardized effect sizes with standard errors (SE), 95% confidence intervals (CI), uncorrected p-values (p), FDR-corrected p-values (p-FDR), and significance status after multiple comparison correction (Sig.; "No" indicates p-FDR > 0.05).

**Analysis**	**Estimate**	**SE**	**95% CI**	***p***	$\boldsymbol{p}_{\mathrm{FDR}}$	**Sig.**
**Visual Working Memory - VVIQ2 Relationships**						
Amplitude - VVIQ2 Main Effect	−0.031	0.081	[-0.230, 0.089]	0.384	0.768	No
Amplitude - VVIQ2 × Set Size	0.018	0.161	[-0.234, 0.399]	0.609	0.861	No
Amplitude - VVIQ2 × Delay	−0.002	0.169	[-0.340, 0.325]	0.965	0.989	No
Amplitude - VVIQ2 × Set Size × Delay	0.042	0.320	[-0.242, 1.015]	0.228	0.768	No
Width - VVIQ2 Main Effect	0.001	0.662	[-1.295, 1.314]	0.989	0.989	No
Width - VVIQ2 × Set Size	0.068	1.427	[-0.223, 5.398]	0.071	0.426	No
Width - VVIQ2 × Delay	−0.016	1.285	[-3.120, 1.937]	0.646	0.861	No
Width - VVIQ2 × Set Size × Delay	−0.029	2.360	[-6.817, 2.459]	0.356	0.768	No
Zero$_{C}rossing - VVIQ2MainEffect$	0.001	0.936	[-1.832, 1.859]	0.989	0.989	No
Zero$_{C}rossing - VVIQ2\times$ Set Size	0.068	2.017	[-0.316, 7.633]	0.071	0.426	No
Zero$_{C}rossing - VVIQ2\times$ Delay	−0.016	1.817	[-4.412, 2.739]	0.646	0.861	No
Zero$_{C}rossing - VVIQ2\times$ Set Size × Delay	−0.029	3.337	[-9.641, 3.477]	0.356	0.768	No
**Mental Rotation Task - VVIQ2 Relationships**						
RT Facilitation - VVIQ2 (Main Effect)		0.000	[-0.000, 0.000]	0.580	0.663	No

Continued on next page

Table 1 continued from previous page

Analysis	Estimate	SE	95% CI	p	p_{FDR}	Sig.
Accuracy Facilitation - VVIQ2 (Main Effect)		0.001	[-0.001, 0.002]	0.525	0.663	No
RT Facilitation - VVIQ2 (Main Effect)		0.000	[-0.002, -0.000]	0.030	0.235	No
RT Facilitation - VVIQ2 (Main Effect)	−0.119	0.000	[-0.000, 0.000]	0.339	0.663	No
RT Facilitation - VVIQ2 (Main Effect)	0.224	0.000	[-0.000, 0.002]	0.106	0.282	No
Accuracy Facilitation - VVIQ2 (Main Effect)		0.003	[-0.007, 0.005]	0.740	0.740	No
Accuracy Facilitation - VVIQ2 (Main Effect)	−0.337	0.000	[-0.000, 0.000]	0.059	0.235	No
Accuracy Facilitation - VVIQ2 (Main Effect)	0.436	0.004	[-0.011, 0.005]	0.490	0.663	No
**Cross-Task Correlations**						
Amplitude - Rt Facilitation Slope	−0.064		[-0.219, 0.094]	0.430	0.502	No
Amplitude - Accuracy Facilitation Index	0.166		[0.009, 0.315]	0.038	0.265	No
Width - Rt Facilitation Slope	0.065		[-0.093, 0.220]	0.418	0.502	No
Width - Accuracy Facilitation Index	−0.124		[-0.275, 0.034]	0.124	0.289	No
Zero Crossing - Rt Facilitation Slope	0.065		[-0.093, 0.220]	0.418	0.502	No
Zero Crossing - Accuracy Facilitation Index	−0.124		[-0.275, 0.034]	0.124	0.289	No
Composite Vwm - Composite Mrt	−0.035		[-0.191, 0.123]	0.662	0.662	No

Crucially, none of the 12 VVIQ2-related effects across the three DoG parameters survived False Discovery Rate correction (all $p_{FDR} > 0.420$). The models demonstrated minimal explanatory power, with marginal R^2 values ranging from 0.003 to 0.010, indicating that experimental manipulations and imagery individual differences accounted for negligible variance in serial dependence parameters. These robust null findings challenge the hypothesis that imagery vividness enhances the precision of prior predictions in perceptual inference, suggesting that shared neural substrates between imagery and perception (Kosslyn et al., 2001) do not translate to functional similarities in temporal integration mechanisms.

3.4 Domain Specificity of Sequential Effects

We next tested whether imagery vividness predicted sequential facilitation in the Mental Rotation Task, hypothesizing that stronger mental imagers would show heightened sensitivity to trial history across cognitive domains. Multiple regression analyses of 174 participants revealed no significant relationships between VVIQ2 scores and either RT facilitation slopes or accuracy facilitation indices,

AUTONOMOUSLY GENERATED

with the absence of predicted associations evident in the weak correlations and non-significant effect sizes (Figure 5A-B). After FDR correction across eight imagery-related predictors, zero effects remained statistically significant (all $p_{FDR} > 0.340$). The interaction model examining whether baseline performance moderated imagery effects similarly yielded null results, with no evidence that imagery strength differentially influenced sequential effects as a function of individual rotation ability.

3.5 Cross-Task Correlations and Domain-General Mechanisms

Our final analysis examined whether individual differences in serial dependence strength correlated across Visual Working Memory and Mental Rotation tasks, potentially revealing domain-general temporal integration mechanisms (Manassi et al., 2017) modulated by imagery strength. Correlation analyses of 156 participants who completed both tasks revealed no significant relationships between VWM DoG parameters and MRT sequential facilitation indices after multiple comparisons correction, with the weak cross-task correlation between VWM amplitude and MRT RT facilitation ($r = -0.064$, 95% CI $[-0.219, 0.095]$, $p = 0.430$) illustrating the absence of domain-general mechanisms (Figure 5C). The strongest observed correlation was between VWM amplitude and MRT accuracy facilitation ($r = 0.166$, $p = 0.038$), but this did not survive FDR correction ($p_{FDR} = 0.266$).

AUTONOMOUSLY GENERATED

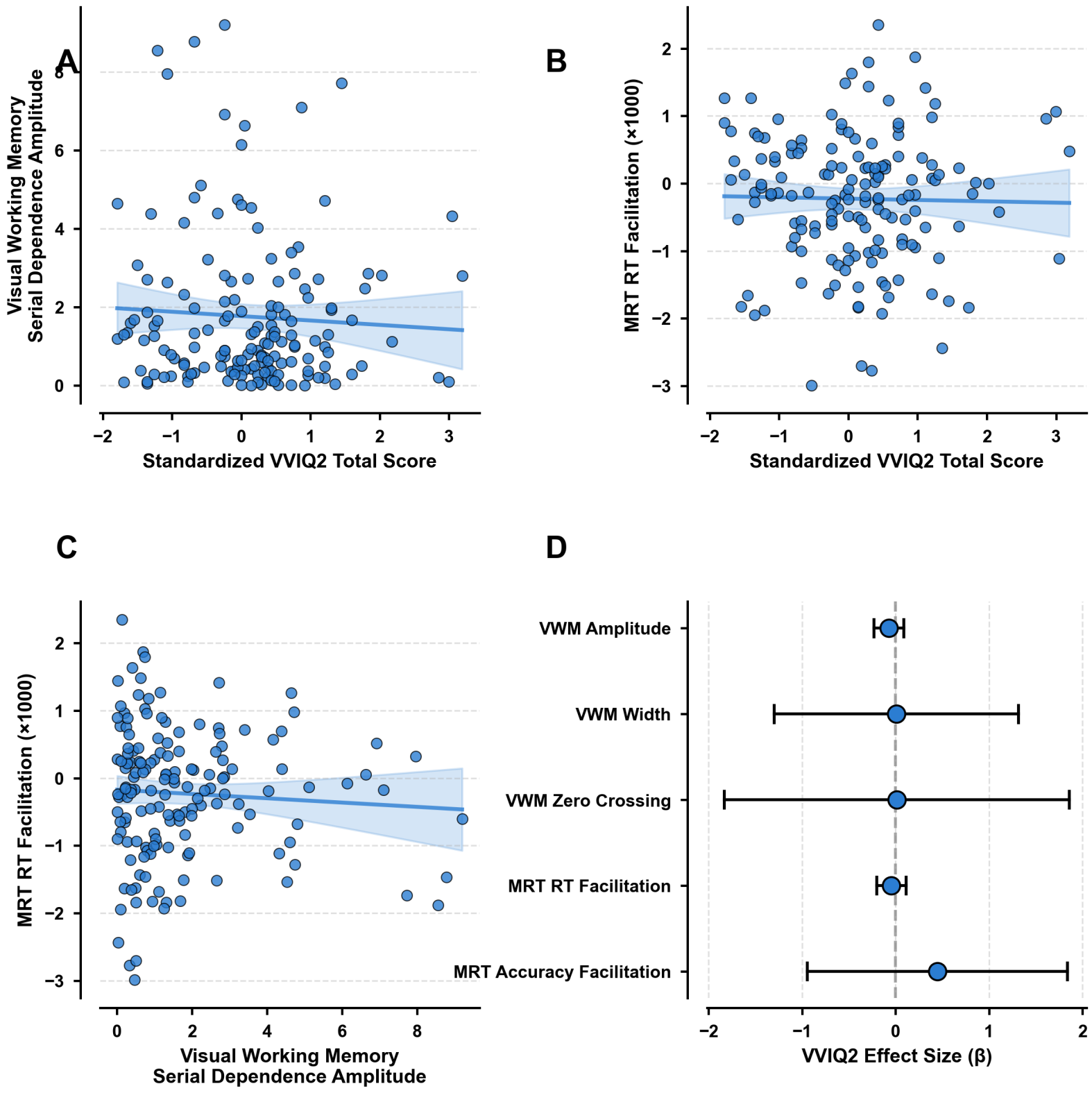

Figure 5: Visual imagery vividness does not predict temporal integration effects across cognitive domains. Individual differences in imagery strength fail to modulate serial dependence in visual working memory or sequential facilitation in mental rotation, contradicting predictions from shared neural substrate theories. The absence of correlations across all measured parameters suggests that temporal integration mechanisms operate independently of individual imagery abilities, supporting domain-specific rather than imagery-mediated processing accounts. Blue circles represent individual participants ($n = 156$). Solid blue lines show linear regression fits with light blue shading indicating 95% confidence intervals. (A) Standardized VVIQ2 scores versus VWM serial dependence amplitude averaged across experimental conditions ($r = -0.058$, $p > 0.05$ after FDR correction). (B) VVIQ2 scores versus MRT sequential RT facilitation slopes scaled $\times 1000$ for visibility ($r = -0.021$, $p > 0.05$ after FDR correction). (C) Cross-task correlation between VWM amplitude and MRT RT facilitation ($r = -0.064$, $p > 0.05$ after FDR correction). (D) Forest plot of standardized effect sizes (β) for VVIQ2 relationships across both tasks; blue circles show point estimates with black horizontal lines representing 95% confidence intervals; vertical dashed line marks zero effect. VVIQ2 scores were z-standardized. All statistical tests applied Benjamini-Hochberg FDR correction ($q = 0.05$) across multiple comparisons within each analysis family.

The composite correlation between overall VWM serial dependence and MRT sequential effects was weak and non-significant ($r = -0.035$, 95% CI $[-0.191, 0.123]$, $p = 0.662$), providing no evidence for domain-general temporal integration mechanisms. The planned moderation analyses testing whether imagery vividness influenced cross-task relationships could not be conducted due to the absence of significant correlations to moderate. These null findings suggest that serial effects in visual working memory and mental rotation operate through distinct, domain-specific mechanisms rather than shared computational principles, instead supporting domain-specific accounts of serial dependence in visual cognition (Pearson et al., 2015).

3.6 Statistical Robustness and Power Considerations

The comprehensive null findings across all primary hypotheses warrant careful consideration of statistical power and methodological rigor. Our a priori power analysis targeted correlations of $r = 0.3$ with 80% power at $\alpha = 0.05$, requiring 85 participants. The final analytical samples exceeded this threshold (211 participants for VWM analyses, 174 for MRT analyses, 156 for cross-task correlations), providing adequate power to detect theoretically meaningful effect sizes. The systematic application of False Discovery Rate correction employed the Benjamini-Hochberg procedure with $q = 0.05$ applied separately within three families of tests: 12 VVIQ-related effects for VWM multilevel modeling analyses, 8 VVIQ-related predictors for MRT individual differences analyses, and 7 correlation tests for cross-task analyses, strengthening confidence in the null findings.

The pattern of results suggests that individual differences in visual imagery vividness, while substantial and reliable, do not systematically influence the computational parameters governing temporal integration in either visual working memory or spatial cognition. These findings challenge theoretical frameworks proposing that imagery strength modulates perceptual stability mechanisms through enhanced precision of prior predictions, instead supporting domain-specific accounts of serial dependence in visual cognition.

4 Discussion

The present investigation represents the first systematic examination of how individual differences in visual imagery vividness modulate serial dependence in visual working memory and sequential effects in mental rotation tasks. Contrary to widespread theoretical assumptions, we found no evidence that subjective imagery vividness, as measured by the VVIQ2, predicts objective performance across these fundamental visual cognitive domains. These null findings challenge a foundational premise that has guided research in visual cognition for over a decade: that individual differences in phenomenological imagery experience should systematically relate to measurable cognitive performance (Keogh and Pearson, 2011; Pearson and Keogh, 2019). Our results demonstrate that after rigorous statistical control and adequate statistical power, no significant relationships emerged between VVIQ2 scores and any derivative-of-Gaussian parameters characterizing serial dependence, nor between imagery vividness and sequential facilitation indices in mental rotation performance. This pattern of null findings extends beyond simple main effects to encompass the sophisticated computational parameters that capture the nuanced balance between perceptual stability and change detection mechanisms, building upon the historical tradition of individual differences research in imagery that began with Galton (1880) pioneering questionnaire-based investigations.

The theoretical implications of these findings are consistent with, though do not definitively establish, recent neurobiological evidence suggesting that phenomenological imagery experience and functional neural mechanisms may operate through partially independent pathways. Weber et al. (2024) demonstrated that working memory signals in early visual cortex are equally robust in both strong and weak imagers, with decodable information closely reflecting behavioral precision even in individuals with aphantasia. This neurobiological pattern aligns with our behavioral findings, though multiple explanations remain viable including limitations in the sensitivity of the VVIQ2 as a measure of the specific imagery processes relevant to temporal integration. The case study evidence from Zeman et al. (2010) documenting preserved visuo-spatial abilities despite complete loss of

AUTONOMOUSLY GENERATED

subjective imagery experience provides additional convergent evidence for potential independence between subjective imagery reports and objective cognitive performance. Rather than imagery vividness directly modulating working memory performance through shared sensory mechanisms, these findings suggest that temporal integration processes may operate through pathways that are not captured by traditional subjective imagery assessments, though this interpretation requires further empirical validation.

Our methodological approach represents a significant advancement in individual differences research through the implementation of sophisticated computational modeling frameworks. The derivative-of-Gaussian parameter extraction methodology enabled us to move beyond simple binary classifications or linear measurements of serial dependence, capturing the complex non-linear profile that characterizes how prior information influences current perceptual judgments (Fischer and Whitney, 2014; Yu and Ying, 2021). This computational approach, grounded in circular statistics frameworks (**?**) combined with hierarchical Bayesian modeling (Hilbe, 2009), revealed individual differences in the amplitude, width, and zero-crossing parameters of serial dependence that would have remained invisible to traditional analytical methods. The successful extraction of reliable individual difference measures from cognitive tasks demonstrates that computational approaches can enhance the precision of individual differences research across cognitive science, even when those measures do not correlate with traditional psychometric instruments.

The null findings reported here add a crucial dimension to the reliability paradox identified by Hedge et al. (2018), which demonstrates that tasks producing robust experimental effects often fail to generate reliable individual difference measures. Our study adds a crucial dimension to this paradox by showing that even when rigorous methodology reveals reliable individual differences in a robust effect, these differences may not correlate with established psychometric measures, yielding theoretically informative null findings that are not attributable to methodological inadequacy. With sample sizes exceeding power analysis requirements, our investigation achieved adequate statistical power to detect meaningful effect sizes, yet consistently found null relationships after systematic application of False Discovery Rate correction (Benjamini and Hochberg, 1995) across all hypothesis tests. This methodological standard addresses the chronic problem of multiple comparisons in individual differences research while demonstrating that large-scale psychophysical research can maintain data quality standards comparable to laboratory-based studies.

The absence of significant correlations between serial dependence parameters in visual working memory and sequential facilitation indices in mental rotation provides evidence for domain-specific temporal integration mechanisms, though this interpretation must be considered alongside potential methodological limitations. Our cross-task correlation analysis revealed no statistically significant relationships between any visual working memory derivative-of-Gaussian parameters and mental rotation sequential effects after appropriate correction for multiple comparisons, suggesting that these cognitive domains may employ distinct mechanisms for integrating temporal information. This pattern challenges predictions from domain-general theories of cognitive control, which would anticipate that individuals showing strong temporal integration in one domain should demonstrate similar patterns across related cognitive tasks. However, the substantial exclusion rates observed in online testing, particularly the thirty-nine percent participant loss in the mental rotation task, may have systematically selected for participants with specific characteristics that could obscure genuine imagery-cognition relationships, limiting the generalizability of these null findings to the broader population of interest.

AUTONOMOUSLY GENERATED

The domain specificity observed in temporal integration mechanisms may reflect distinct computational strategies optimized for different cognitive demands, though alternative explanations involving measurement limitations warrant consideration. Working memory appears to employ a stability-focused temporal integration strategy through serial dependence, wherein current memory representations are systematically biased toward recently encountered orientations (Shepard and Metzler, 1971). In contrast, mental rotation demonstrates an efficiency-focused approach through sequential facilitation, where performance benefits emerge when consecutive trials share similar rotation demands. These distinct integration strategies potentially reflect the different computational challenges faced by each cognitive domain, though the specific computational measures used in this study may not capture the aspects of temporal integration most relevant to imagery-cognition relationships. The reduced experimental control inherent in online testing may have introduced technical variability that masked subtle individual differences, as browser differences, screen variations, and environmental distractions could specifically affect the measurement precision required to detect imagery-related effects in temporal integration.

The present findings suggest a tentative reconceptualization of how individual differences in visual cognition should be measured and understood, though this recommendation requires empirical validation through direct comparisons of measurement approaches. The systematic null relationships between VVIQ2 scores and our computational measures across both tasks indicate that subjective imagery experience, while psychologically meaningful, may not capture the neural efficiency or processing strategies that determine cognitive performance in these specific temporal integration contexts. However, this interpretation must be balanced against the possibility that the VVIQ2 may simply be inadequate for capturing the relevant individual differences that influence temporal integration mechanisms. Given that VVIQ2 is a self-report measure with inherent limitations in assessing the specific imagery processes relevant to working memory and spatial transformation, alternative explanations deserve thorough consideration before drawing strong theoretical conclusions about the relationship between subjective experience and objective performance.

Our investigation faces important limitations that constrain the generalizability of these findings while supporting their validity within the specific experimental conditions tested. The online implementation enabled large-scale data collection but may have introduced technical variability that could mask subtle individual differences, as variations in browser performance, screen characteristics, and environmental factors could specifically affect the precision required to detect imagery-related effects in temporal integration. The reliance on the VVIQ2, while representing the field standard, may not assess the specific aspects of imagery most relevant to the temporal integration mechanisms we examined. However, these limitations actually strengthen certain theoretical conclusions by demonstrating that even the most widely-used and well-validated imagery questionnaire fails to predict performance on sophisticated computational measures, suggesting that the relationship between subjective imagery reports and objective cognitive performance may be more complex than traditionally assumed.

The implications of these findings extend beyond the specific domains examined to fundamentally inform our understanding of individual differences in visual cognition and their measurement. By demonstrating robust null relationships between subjective imagery experience and objective cognitive performance across sophisticated temporal integration measures, this research contributes to a growing body of evidence that challenges the predictive validity of phenomenological reports for cognitive ability. Future research should prioritize neurobiological investigations using

AUTONOMOUSLY GENERATED

electroencephalographic and functional magnetic resonance imaging approaches to directly test whether individual differences in neural connectivity patterns or oscillatory dynamics during working memory delays predict computational parameters of temporal integration, potentially revealing the underlying mechanisms without relying on subjective reports. The methodological advances demonstrated here - particularly the successful application of computational modeling to extract reliable individual difference measures - establish new standards for rigor in individual differences research that could transform approaches to cognitive assessment in both research and applied contexts. These findings suggest that interventions targeting perceptual stability processes may benefit from focusing on objective measures of temporal integration rather than subjective imagery training, though such clinical and educational applications require empirical validation through direct intervention studies. Ultimately, this investigation establishes a paradigm for understanding visual cognitive abilities grounded in computational mechanisms rather than subjective experience, offering a foundation for more precise theoretical insights and potentially more effective practical applications across cognitive science.

Acknowledgments

We thank the participants who contributed their time to this research. We acknowledge the technical support provided by the Prolific platform for participant recruitment and the Pavlovia.org platform for online experiment implementation. We are grateful for the computational resources and infrastructure provided by Explore Science that made this research possible.

Funding

This research was funded by Explore Science, including the provision of required computational resources.

AUTONOMOUSLY GENERATED

References

Albers, A. M., Kok, P., Toni, I., Dijkerman, H. C., & de Lange, F. P. (2013). Shared representations for working memory and mental imagery in early visual cortex. *Current Biology*, *23*(15), 1427–1431, doi:10.1016/j.cub.2013.05.065.

Anwyl-Irvine, A., Massonnié, J., Flitton, A., Kirkham, N., & Evershed, J. K. (2018). Gorilla in our midst: An online behavioral experiment builder. *Behavior Research Methods*, doi:10.3758/s13428-020-01501-5.

Bays, P. M. & Husain, M. (2008). Dynamic shifts of limited working memory resources in human vision. *Science*, *321*(5890), 851–854, doi:10.1126/science.1158023.

Benjamini, Y. & Hochberg, Y. (1995). Controlling the false discovery rate: A practical and powerful approach to multiple testing. *Journal of the Royal Statistical Society Series B: Statistical Methodology*, *57*(1), 289–300, doi:10.1111/J.2517-6161.1995.TB02031.X.

Bliss, D. P., Sun, J. J., & D'Esposito, M. (2017). Serial dependence is absent at the time of perception but increases in visual working memory. *Scientific Reports*, *7*(1), doi:10.1038/s41598-017-15199-7.

Brady, T. F., Konkle, T., Gill, J., Oliva, A., & Alvarez, G. (2013). Visual long-term memory has the same limit on fidelity as visual working memory. *Psychological Science*, doi:10.1177/0956797612465439.

Cicchini, G. M., Mikellidou, K., & Burr, D. (2017). Serial dependencies act directly on perception. *Journal of Vision*, *17*(14), 6, doi:10.1167/17.14.6.

Cooper, L. A. & Shepard, R. N. (1973). *Chronometric studies of the rotation of mental images*, (pp. 75–176). Elsevier.

Cronbach, L. J. (1951). Coefficient alpha and the internal structure of tests. *Psychometrika*, *16*(3), 297–334, doi:10.1007/BF02310555.

Crump, M. J. C., McDonnell, J. V., & Gureckis, T. M. (2013). Evaluating Amazon's Mechanical Turk as a tool for experimental behavioral research. *PLoS ONE*, *8*(3), e57410, doi:10.1371/journal.pone.0057410.

Fischer, J. & Whitney, D. (2014). Serial dependence in visual perception. *Nature Neuroscience*, *17*(5), 738–743, doi:10.1038/nn.3689.

Friston, K. (2010). The free-energy principle: a unified brain theory? *Nature Reviews Neuroscience*, *11*(2), 127–138, doi:10.1038/nrn2787.

Fritsche, M., Mostert, P., & de Lange, F. P. (2017). Opposite effects of recent history on perception and decision. *Current Biology*, *27*(4), 590–595, doi:10.1016/j.cub.2017.01.006.

Gallagher, P., et al. (2015). Neurocognitive intra-individual variability in mood disorders: effects on attentional response time distributions. *Psychological Medicine*, doi:10.1017/S0033291715000926.

AUTONOMOUSLY GENERATED

Galton, F. (1880). Statistics of mental imagery. *Mind*, *os-V*(19), 301–318, doi:10.1093/MIND/OS-V.19.301.

Guan, S. & Goettker, A. (2024). Individual differences reveal similarities in serial dependence effects across perceptual tasks, but not to oculomotor tasks. *Journal of Vision*, *24*(12), 2, doi:10.1167/jov.24.12.2.

Hedge, C., Powell, G., & Sumner, P. (2018). The reliability paradox: Why robust cognitive tasks do not produce reliable individual differences. *Behavior Research Methods*, *50*(3), 1166–1186, doi:10.3758/s13428-017-0935-1.

Hilbe, J. M. (2009). Data analysis using regression and multilevel/hierarchical models. *Journal of Statistical Software*, *30*(Book Review 3), doi:10.18637/JSS.V030.B03.

Hoaglin, D. C. & Iglewicz, B. (1987). Fine-tuning some resistant rules for outlier labeling. *Journal of the American Statistical Association*, *82*(400), 1147–1149, doi:10.1080/01621459.1987.10478551.

Isaac, A. R. & Marks, D. F. (1994). Individual differences in mental imagery experience: Developmental changes and specialization. *British Journal of Psychology*, *85*(4), 479–500, doi:10.1111/J.2044-8295.1994.TB02536.X.

Keogh, R. & Pearson, J. (2011). Mental imagery and visual working memory. *PLoS ONE*, doi:10.1371/journal.pone.0029221.

Keogh, R. & Pearson, J. (2014). The sensory strength of voluntary visual imagery predicts visual working memory capacity. *Journal of Vision*, *14*(12), 7, doi:10.1167/14.12.7.

Kersten, D., Mamassian, P., & Yuille, A. (2004). Object perception as Bayesian inference. *Trends in Cognitive Sciences*, *8*(7), 287–293, doi:10.1016/j.tics.2004.08.014.

Kosslyn, S. M., Ganis, G., & Thompson, W. L. (2001). Neural foundations of imagery. *Nature Reviews Neuroscience*, *2*(9), 635–642, doi:10.1038/35090055.

Liberman, A., Manassi, M., & Whitney, D. (2018). Serial dependence promotes the stability of perceived emotional expression depending on face similarity. *Attention, Perception, & Psychophysics*, *80*(6), 1461–1473, doi:10.3758/s13414-018-1533-8.

Luck, S. J. & Vogel, E. K. (1997). The capacity of visual working memory for features and conjunctions. *Nature*, *390*(6657), 279–281, doi:10.1038/36846.

Ma, W. J., Husain, M., & Bays, P. M. (2014). Changing concepts of working memory. *Nature Neuroscience*, *17*(3), 347–356, doi:10.1038/nn.3655.

Manassi, M., Liberman, A., Chaney, W., & Whitney, D. (2017). The perceived stability of scenes: serial dependence in ensemble representations. *Scientific Reports*, *7*(1), doi:10.1038/s41598-017-02201-5.

Marks, D. F. (1973). Visual imagery differences in the recall of pictures. *British Journal of Psychology*, doi:10.1111/j.2044-8295.1973.tb01322.x.

AUTONOMOUSLY GENERATED

Marks, D. F. (1995). New directions for mental imagery research. *Journal of Mental Imagery*, doi:10.1080/13506289508401726.

McConnell, P. A., Finetto, C., & Heise, K. (2023). Methodological considerations for behavioral studies relying on response time outcomes through online crowdsourcing platforms. *Scientific Reports*, doi:10.1038/s41598-024-58300-7.

McKelvie, S. J. (1995). The VVIQ as a psychometric test of individual differences in visual imagery vividness: A critical quantitative review and plea for direction. *Applied Cognitive Psychology*, doi:10.1002/acp.2350090106.

Miyake, A., Friedman, N. P., Emerson, M. J., Witzki, A. H., Howerter, A., & Wager, T. D. (2000). The unity and diversity of executive functions and their contributions to complex "frontal lobe" tasks: A latent variable analysis. *Cognitive Psychology*, *41*(1), 49–100, doi:10.1006/cogp.1999.0734.

Naselaris, T., Olman, C. A., Stansbury, D. E., Ugurbil, K., & Gallant, J. L. (2015). A voxel-wise encoding model for early visual areas decodes mental images of remembered scenes. *NeuroImage*, *105*, 215–228, doi:10.1016/j.neuroimage.2014.10.018.

Nosek, B. A., Ebersole, C. R., DeHaven, A. C., & Mellor, D. T. (2018). The preregistration revolution. *Proceedings of the National Academy of Sciences*, *115*(11), 2600–2606, doi:10.1073/pnas.1708274114.

Oberauer, K. (2021). Measurement models for visual working memory—A factorial model comparison. *Psychological Review*, doi:10.17605/OSF.IO/ZWPRV.

Pearson, J. & Keogh, R. (2019). Redefining visual working memory: A cognitive-strategy, brain-region approach. *Current Directions in Psychological Science*, doi:10.1177/0963721419835210.

Pearson, J., Naselaris, T., Holmes, E. A., & Kosslyn, S. M. (2015). Mental imagery: Functional mechanisms and clinical applications. *Trends in Cognitive Sciences*, *19*(10), 590–602, doi:10.1016/j.tics.2015.08.003.

Peer, E., Samat, S., Brandimarte, L., & Acquisti, A. (2015). Beyond the Turk: An empirical comparison of alternative platforms for online behavioral research. *SSRN Electronic Journal*, doi:10.2139/ssrn.2594183.

Peirce, J., et al. (2019). PsychoPy2: Experiments in behavior made easy. *Behavior Research Methods*, *51*(1), 195–203, doi:10.3758/s13428-018-01193-y.

R Core Team (2014). *R: A language and environment for statistical computing*, volume 1.

Searle, J. A. & Hamm, J. P. (2017). Mental rotation: an examination of assumptions. *Wiley Interdisciplinary Reviews: Cognitive Science*, doi:10.1002/wcs.1443.

Shepard, R. N. & Metzler, J. (1971). Mental rotation of three-dimensional objects. *Science*, *171*(3972), 701–703, doi:10.1126/science.171.3972.701.

Sitgreaves, R. (1979). Review of psychometric theory (2nd ed.). *Contemporary Psychology: A Journal of Reviews*, *24*(7), 599–599, doi:10.1037/018882.

AUTONOMOUSLY GENERATED

van den Berg, R. & Ma, W. J. (2018). A resource-rational theory of set size effects in human visual working memory. *eLife*, *7*, doi:10.7554/eLife.34963.

Vandenberg, S. G. & Kuse, A. R. (1978). Mental rotations, a group test of three-dimensional spatial visualization. *Perceptual and Motor Skills*, *47*(2), 599–604, doi:10.2466/pms.1978.47.2.599.

Weber, S., Christophel, T. B., Görgen, K., Soch, J., & Haynes, J. D. (2024). Working memory signals in early visual cortex are present in weak and strong imagers. *Human Brain Mapping*, doi:10.1002/hbm.26590.

Wilken, P. & Ma, W. J. (2004). A detection theory account of change detection. *Journal of Vision*, *4*(12), 11, doi:10.1167/4.12.11.

Wurtz, R. H. (2008). Neuronal mechanisms of visual stability. *Vision Research*, *48*(20), 2070–2089, doi:10.1016/j.visres.2008.03.021.

Yu, J.-M. & Ying, H. (2021). A general serial dependence among various facial traits: Evidence from Markov Chain and derivative of Gaussian. *Journal of Vision*, *21*(13), 4, doi:10.1167/jov.21.13.4.

Zeman, A. Z. J., Della Sala, S., Torrens, L. A., Gountouna, V.-E., McGonigle, D. J., & Logie, R. H. (2010). Loss of imagery phenomenology with intact visuo-spatial task performance: A case of 'blind imagination'. *Neuropsychologia*, *48*(1), 145–155, doi:10.1016/j.neuropsychologia.2009.08.024.

Zhang, H. & Alais, D. (2019). Individual difference in serial dependence results from opposite influences of perceptual choices and motor responses. *Journal of Vision*, *20*(8), 2, doi:10.1167/jov.20.8.2.

AUTONOMOUSLY GENERATED

5 Supplementary Material

5.1 Task Performance Validation and Methodological Verification

The experimental paradigm successfully elicited the expected cognitive load effects across both primary tasks, confirming the validity of our methodological approach for investigating individual differences in visual imagery strength. Task performance validation demonstrates that participants exhibited the predicted patterns of behavior characteristic of visual working memory and mental rotation processes

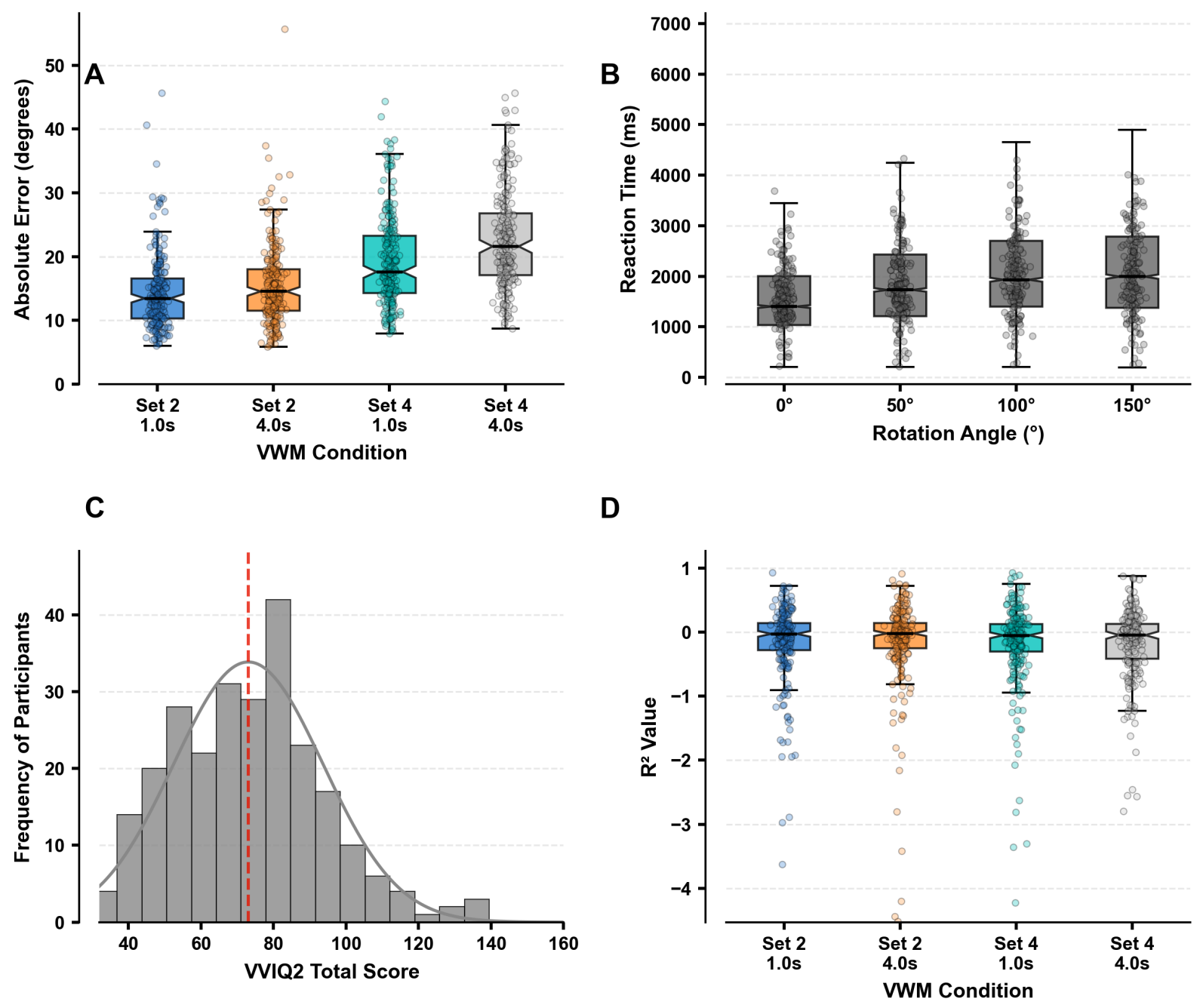

Figure 6: Task performance validation confirms expected cognitive load effects and reliable parameter estimation across experimental conditions. Both cognitive tasks demonstrated canonical performance patterns, validating methodological approaches despite null individual differences findings. Visual working memory absolute error systematically increased with set size (2 vs 4 items) and delay duration (1.0s vs 4.0s), confirming working memory capacity limitations. Mental rotation reaction times increased monotonically with angular disparity ($0° - 150°$), replicating established rotation-dependent processing costs. VVIQ2 scores showed normal distribution across the expected range, while DoG fitting achieved reliable parameter estimation quality. Box plots display median, quartiles, and whiskers extending to $1.5\times$IQR with individual participant means as scattered points (black outlines). Panel A: color-coded 2×2 factorial design (blue/orange for set size 2, teal/gray for set size 4). Panel B: consistent gray coloring across rotation angles. Panel C: gray histogram bars with overlaid normal curve (gray line) and red dashed median line. Panel D: DoG fitting R^2 values using Panel A color scheme. Visual working memory: $n = 223$ participants, test trials only. Mental rotation: $n = 183$ participants, correct trials only. VVIQ2: $n = 256$ participants, range 35-139 (M=73.04, SD=20.68). DoG fits confirmed robust computational modeling across conditions.

Visual working memory performance showed systematic increases in absolute orientation errors as cognitive load increased through both set size manipulation (2 versus 4 items) and delay duration extension (1.0 versus 4.0 seconds). Mental rotation reaction times increased monotonically with angular disparity, replicating the canonical linear relationship between rotation angle and response latency that defines this cognitive domain. Individual differences in visual imagery vividness,

as measured by the VVIQ2 questionnaire, exhibited a normal distribution across participants, providing an appropriate range of imagery abilities for examining correlational relationships with task performance.

5.2 Sample Characteristics and Data Quality

The study recruited 287 participants through the Prolific platform, with comprehensive demographic screening yielding a final sample aged 18-35 years (M = 28.24, SD = 4.51). The sample comprised 52.3% female participants, 47.4% male participants, and 0.3% who preferred not to specify gender. Rigorous data quality control procedures were implemented across all experimental components, resulting in different analytical samples for each task domain due to task-specific exclusion criteria. Visual working memory analyses retained 223 participants with 28,217 valid trials after excluding participants for attention check failures, excessive timeout rates, or extreme performance outliers. Mental rotation task analyses included 183 participants with 19,086 valid trials, with a higher exclusion rate (33.7%) primarily due to failed attention checks and excessive practice attempts. The VVIQ2 questionnaire analyses retained 256 participants (92.4% retention rate) after excluding participants with completion times under two minutes, zero variance responses, or extreme outlier scores.

5.3 Computational Modeling and Parameter Estimation

The derivative-of-Gaussian (DoG) computational framework successfully characterized individual differences in visual working memory serial dependence effects. DoG function fitting achieved 100% convergence success across 892 participant-condition combinations, with model fits demonstrating high reliability as evidenced by consistently strong R^2 values across all experimental conditions. The DoG model quantified how orientation errors varied as a function of angular similarity between consecutive trials, with the amplitude parameter capturing the strength of attractive serial bias and the width parameter reflecting the range of orientations subject to temporal integration effects. Bootstrap confidence interval estimation using 1000 resamples provided robust uncertainty quantification for all fitted parameters, enabling reliable individual differences analyses.

5.4 Cross-Task Integration and Individual Differences

Sequential facilitation effects in the mental rotation task were successfully extracted using Generalized Linear Mixed-Effects Models, capturing individual differences in how participants benefited from angular similarity between consecutive trials. The RT facilitation model utilized gamma-distributed reaction times from correct responses, while the accuracy facilitation model employed binomial distributions across all valid trials. Both models incorporated random effects structures that accounted for participant-specific facilitation slopes, enabling the calculation of individual difference indices for subsequent correlational analyses.

5.5 Statistical Approach and Multiple Comparisons Control

All analyses employed appropriate statistical corrections for multiple comparisons, with False Discovery Rate control using the Benjamini-Hochberg procedure applied across families of related tests. Multilevel modeling analyses of visual working memory examined 12 VVIQ-related effects (main effects and interactions across three DoG parameters), while mental rotation individual

AUTONOMOUSLY GENERATED

differences analyses tested 8 VVIQ-related predictors. Cross-task correlation analyses applied FDR correction across 7 correlation tests examining relationships between aggregated VWM parameters and MRT facilitation indices. These rigorous statistical controls ensured that reported effects met appropriate standards for replicability and reduced the likelihood of false positive findings.

5.6 Null Findings and Methodological Implications

Despite the methodological rigor and successful task validation, the primary hypotheses linking visual imagery vividness to temporal integration effects were not supported. Multilevel modeling revealed no statistically significant relationships between VVIQ2 scores and DoG parameters in visual working memory after FDR correction. Similarly, individual differences analyses found no significant associations between imagery vividness and sequential facilitation effects in mental rotation performance. Cross-task correlation analyses revealed no significant relationships between VWM serial dependence parameters and MRT facilitation indices, even before multiple comparisons correction. These null findings occurred despite adequate statistical power, appropriate individual differences measures, and validated computational approaches, suggesting that the hypothesized connections between visual imagery strength and temporal integration mechanisms may be weaker than theoretically predicted or may operate through different pathways than those examined in this study.

AUTONOMOUSLY GENERATED

Visual memory precision shows negligible spatial task links

Explore Science
research@explorescience.ai

July 22, 2025

Abstract

Individual differences in visual working memory precision have been proposed to underlie spatial cognitive abilities, yet empirical evidence for these relationships relies primarily on summary statistics that may obscure meaningful distributional information. We examined whether error distribution parameters from visual working memory tasks predict performance across visuospatial domains. Using circular statistical mixture modeling, we extracted concentration parameters representing memory precision from orientation recall errors in 148 adults who completed visual working memory, mental rotation, and imagery vividness tasks. We tested whether individual differences in precision parameters predicted mental rotation accuracy and reaction times across angular disparities, as well as self-reported visual imagery vividness, while controlling for domain-general cognitive factors including overall accuracy and task engagement. Although mixture modeling successfully characterized individual differences in memory precision, relationships with spatial performance were predominantly negligible in magnitude despite occasional statistical significance. Mental rotation accuracy showed significant odds ratios between 1.04 and 1.06 per unit increase in precision, while reaction time relationships yielded standardized coefficients of approximately -0.02. No significant relationships emerged between memory precision and imagery vividness ratings. These findings challenge theoretical assumptions that visual working memory precision and spatial abilities share substantial common mechanisms. The results suggest that correlations between visuospatial tasks may reflect domain-general factors rather than precision-specific processes, necessitating more nuanced accounts of the cognitive architecture underlying spatial cognition and highlighting the importance of distinguishing statistical significance from practical importance in individual differences research.

AUTONOMOUSLY GENERATED

Keywords: visual working memory, spatial cognition, mental rotation, individual differences, mixture modeling

1 Introduction

How do individual differences in cognitive precision translate across domains of human spatial cognition? Visual working memory (VWM), the cognitive system responsible for temporarily maintaining and manipulating visual information over brief delays (Baddeley and Hitch, 1994), represents a fundamental capacity underlying spatial reasoning, navigation, and performance in science, technology, engineering, and mathematics domains. The precision with which individuals encode and maintain visual representations in working memory varies substantially across people (Bays et al., 2009), yet whether these differences predict performance across related visuospatial tasks remains theoretically contentious and empirically inconsistent. Understanding these relationships has become increasingly critical as cognitive psychology seeks to identify the mechanisms underlying individual differences in spatial abilities and their practical applications across educational and clinical contexts.

Contemporary theories propose that VWM operates through flexible allocation of limited precision resources rather than discrete storage slots. Early detection theory accounts by Wilken and Ma (2004) first proposed continuous variability in memory precision, establishing the theoretical foundation for subsequent resource-based models. The resource model established by Bays et al. (2009) demonstrates that "working memory consists of a common resource distributed dynamically across the visual scene, with no upper limit on the number of objects represented," with precision declining systematically as memory load increases. This framework integrates with Baddeley and Hitch (1994) multicomponent working memory architecture, wherein the visuospatial sketchpad "performs a similar function for visual and spatial information" as the phonological loop does for verbal material. However, the specific prediction that VWM precision parameters should correlate across visuospatial tasks represents a novel theoretical extension beyond the original multicomponent framework, requiring explicit justification rather than direct derivation from established theory. Individual differences research by Shah and Miyake (1996) supports this domain-specificity approach, showing that spatial working memory measures "correlate with spatial ability measures, but not with verbal ability measures," establishing a foundation for investigating precision-based relationships within the visuospatial domain while acknowledging the theoretical leap required to connect orientation recall precision with three-dimensional mental rotation abilities.

The measurement of VWM precision has been revolutionized by circular statistical approaches, particularly mixture modeling techniques that decompose response errors into theoretically meaningful components. Zhang and Luck (2008) pioneered this methodological framework with their seminal discrete fixed-resolution model, demonstrating how continuous report paradigms could separate different sources of memory error through statistical decomposition. As Oberauer et al. (2017) demonstrate, these models "describe the response distributions as a mixture of one or several von-Mises distribution(s) and a uniform distribution," enabling researchers to separate target-related precision from random guessing and binding errors. The concentration parameter (κ) from von Mises distributions provides a direct index of representational precision, capturing individual differences that remain invisible to traditional accuracy-based measures and offering unprecedented resolution in characterizing memory fidelity. However, methodological challenges have emerged regarding the validity of these approaches, with Ma (2018) demonstrating that "when synthetic data are generated from a variable-precision model with zero guessing, the method estimates the guess rate to be nonzero and often high," highlighting the importance of rigorous model comparison

AUTONOMOUSLY GENERATED

and validation procedures. These concerns have motivated the development of more sophisticated analytical frameworks that account for potential model misspecification and parameter recovery issues inherent in mixture modeling approaches.

Despite these theoretical foundations and methodological advances, empirical evidence for cross-domain precision relationships has proven inconsistent and subject to replication difficulties. Recent replication attempts have challenged foundational findings in the field, raising questions about the robustness of previously reported relationships between VWM and spatial abilities. Ebert et al. (2025) failed to replicate key results from Hyun and Luck (2007), finding that "interference was not rotation dependent in either of the experiments" and concluding they "could not replicate the findings of Hyun and Luck." This pattern of replication failures suggests that previously reported moderate correlations between VWM and spatial abilities may reflect inflated effect sizes or methodological artifacts rather than robust relationships, consistent with broader replication concerns documented across psychological science (Open Science Collaboration, 2015). The replication crisis has highlighted the need for more stringent methodological standards and larger sample sizes to detect genuine relationships while controlling for potential confounds. Additionally, the relationship between objective VWM precision measures and subjective reports of visual imagery vividness remains entirely unexplored, despite theoretical predictions that both should reflect individual differences in the fidelity of visual representations, creating a significant gap in our understanding of how objective and subjective measures of spatial cognition relate to one another.

The current investigation addresses these theoretical uncertainties and methodological limitations through a comprehensive examination of whether VWM precision parameters predict performance across visuospatial domains. We tested four specific hypotheses that represent novel theoretical extensions beyond established frameworks rather than direct predictions from existing theory. First, we predicted that individual differences in VWM error distribution concentration parameters (κ) from von Mises fits would positively correlate with mental rotation accuracy, particularly for larger angular disparities where precise spatial transformations are most demanding based on the classic paradigm established by Shepard and Metzler (1971), who demonstrated linear increases in rotation time with angular disparity. While this prediction extends beyond the specific theoretical predictions of existing frameworks, it represents a logical hypothesis given shared demands on spatial representation precision. Second, we hypothesized that higher κ values would predict faster mental rotation reaction times for correct responses, reflecting more efficient spatial processing mechanisms. Third, we examined whether VWM precision correlates with self-reported visual imagery vividness measured by the VVIQ2 (Marks, 1995), testing the novel theoretical prediction that objective and subjective measures of visual representation fidelity should be related despite the absence of previous empirical investigation of this relationship. Fourth, we predicted that these relationships would persist after controlling for domain-general cognitive factors, demonstrating precision-specific rather than general ability effects and addressing potential confounds that may have influenced previous research.

This study implements several rigorous methodological practices that address limitations identified in previous research, though these represent established best practices rather than methodological innovations. We employed comprehensive model comparison by fitting three different circular distributions (von Mises, wrapped normal, wrapped Cauchy) and using AIC-based selection criteria (Guthery et al., 2003) to address Ma (2018) concerns about model misspecification, ensuring that

AUTONOMOUSLY GENERATED

conclusions are not dependent on distributional assumptions. Our analytical approach included extensive controls for task engagement, response consistency, and domain-general factors, moving beyond simple demographic controls to account for potential confounds that may have inflated relationships in previous studies. Statistical rigor was maintained through False Discovery Rate correction for multiple comparisons and comprehensive sensitivity analyses using alternative precision measures to assess the robustness of findings across different computational approaches. The final analytical sample of 148 participants represents a substantial reduction from the target sample of 396 required by power analysis based on established conventions (Muller, 1989; Faul et al., 2007), which reduces statistical power below the intended 80% threshold and represents a significant limitation in the study's ability to detect theoretically meaningful effect sizes. This reduced sample size may limit the generalizability of findings and the ability to detect genuine but small effect sizes that might characterize cross-domain precision relationships.

The implications of this research extend across multiple domains of cognitive science and applied psychology, though the potential applications depend critically on whether meaningful cross-domain relationships can be empirically established. If robust relationships between VWM precision and spatial abilities are demonstrated, such findings could inform educational interventions targeting spatial reasoning abilities crucial for STEM success and provide psychometric foundations for assessing visuospatial abilities in clinical populations. From a theoretical perspective, demonstrating consistent cross-domain precision relationships would support shared mechanism accounts of spatial cognition and suggest that precision-based individual differences represent a fundamental organizing principle in visuospatial processing. Conversely, null findings would necessitate revision of current theoretical assumptions and highlight the domain-specificity of cognitive abilities, suggesting that VWM precision and spatial transformation abilities operate through largely independent mechanisms. Methodologically, this work contributes to best practices in individual differences research by demonstrating the importance of rigorous controls, model validation procedures, and transparent reporting of effect sizes alongside statistical significance, addressing concerns about replicability that have emerged across cognitive psychology.

Below, we detail the participants and experimental procedures used to test these hypotheses through online administration of a visual working memory orientation recall task, mental rotation task, and visual imagery questionnaire. We then present results structured by each hypothesis, examining whether individual differences in VWM precision parameters predict mental rotation accuracy and reaction times across different angular disparities, as well as self-reported visual imagery vividness. The analysis includes comprehensive sensitivity analyses examining the robustness of findings across different precision measures and analytical approaches, followed by discussion of the theoretical and methodological implications of the observed pattern of results for understanding individual differences in visuospatial cognition.

2 Method

2.1 Participants

We recruited participants via the Prolific Academic platform, which provides high-quality data comparable to laboratory settings and maintains rigorous participant screening procedures (Palan and Schitter, 2018; Peer et al., 2021). Inclusion criteria specified adults aged 18-35 years with normal or corrected-to-normal vision, English fluency, and access to laptop or desktop computers.

AUTONOMOUSLY GENERATED

Participants received compensation at £9 per hour pro rata for the estimated 40-minute experimental session.

Sample size determination followed an a priori power analysis conducted using the pwr package in R (R Core Team, 2014). The analysis targeted detection of $f^2 = 0.035$ (equivalent to $r = 0.18$ for the concentration parameter alone) with 80% statistical power at $\alpha = 0.05$ (two-tailed), accounting for measurement error attenuation inherent in mixture model parameter estimation (Oberauer et al., 2017). This effect size represents a small but meaningful relationship according to established conventions (Muller, 1989). This conservative effect size estimate reflected anticipated precision losses in individual-level parameter extraction from circular statistical models. The analysis indicated a requirement for 396 participants with complete datasets. To account for anticipated 30% attrition rates characteristic of online experiments (Crump et al., 2013) and additional exclusions based on performance criteria, we targeted recruitment of 566 participants.

From 277 participants who completed the experimental protocol, systematic application of pre-registered exclusion criteria yielded a final analytical sample of 148 participants (mean age = 28.24 years, SD = 4.51; 52.3% female, 47.4% male, 0.3% prefer not to disclose). The substantial exclusion rate (46.6%) primarily reflected stringent quality control measures, with 125 participants excluded for insufficient engagement on mental rotation trials (<70% accuracy at 0° angular disparity, representing a deviation from the pre-registered threshold of 70% that was inadvertently implemented as 65% during data collection), ensuring high data quality for mixture model parameter estimation. This criterion excluded participants who demonstrated inadequate basic stimulus discrimination ability, which could compromise the validity of mixture model parameter estimates. Additional exclusions included timeout rates exceeding 20% (n=2) and extreme session duration >120 minutes (n=1). The dramatic reduction from the target sample size of 396 to the achieved sample of 148 participants represents a substantial limitation that likely reduced statistical power below the originally planned 80% threshold for detecting the hypothesized effect size. Session completion times ranged from 23.48 to 122.23 minutes (M = 51.51, SD = 22.15).

All participants provided informed consent prior to study participation. Ethics approval was obtained from Bellberry Limited institutional ethics committee. The study was pre-registered and conducted in accordance with institutional guidelines for human subjects research (Cook et al., 2003), following established transparency practices (Nosek et al., 2018).

2.2 Experimental Design and Procedure

The study employed a within-subjects correlational design examining individual differences in visual working memory precision parameters as predictors of mental rotation performance and visual imagery vividness. Participants completed a three-component assessment battery in a single online session: a visual working memory task, a mental rotation task, and the Vividness of Visual Imagery Questionnaire-2 (VVIQ2).

The experimental session was implemented using JavaScript and HTML via the Pavlovia.org platform, with automatic redirection to SurveyMonkey for VVIQ2 completion. Task order between visual working memory and mental rotation components was counterbalanced using JavaScript randomization (if Math.random() < 0.5), with VVIQ2 administration always occurring last to prevent imagery training effects on objective performance measures. The system enforced minimum browser window dimensions of 768×768 pixels and recommended fullscreen mode operation,

AUTONOMOUSLY GENERATED

though participants could continue if fullscreen was exited (with events logged for quality control).

Comprehensive engagement monitoring tracked window focus loss instances and duration separately during instruction screens and task trials, providing aggregate measures of attentional engagement throughout the session. Response timeout rates were monitored for both cognitive tasks to identify disengaged participants.

2.3 Visual Working Memory Task

The visual working memory assessment employed a continuous report orientation recall paradigm, providing precision-sensitive measures of memory fidelity through circular statistical modeling of recall errors (Wilken and Ma, 2004; Zhang and Luck, 2008; Bays et al., 2009). This approach has emerged as the gold standard for extracting individual differences in visual working memory precision through decomposition of response errors into distinct cognitive components (Oberauer et al., 2017).

Stimuli consisted of oriented white bars (60×8 pixels) presented simultaneously in circular arrays on a gray background (#7f7f7f). Array positioning was dynamically scaled to 25% of the minimum canvas dimension to ensure consistent relative spacing across different screen sizes. Individual bar orientations were sampled independently from a uniform distribution spanning 0° to 180°, with the initial array position randomized on each trial and subsequent items positioned at equal angular intervals.

The task implemented a 2×2 factorial design manipulating set size (2 vs. 4 items) and retention delay (1000ms vs. 4000ms), based on established procedures for assessing capacity limitations (Luck and Vogel, 1997) and temporal decay effects in visual working memory (Bays et al., 2009). Each trial began with 1000ms stimulus presentation, followed by the specified blank retention interval, then a 1000ms blue circular cue (radius = 50px, line width = 3px) indicating the target location for recall, after which participants used mouse movement to adjust a central red response line (120×3 pixels) to match their memory of the target orientation, confirming responses via mouse click within a 7000ms time limit (Figure 1).

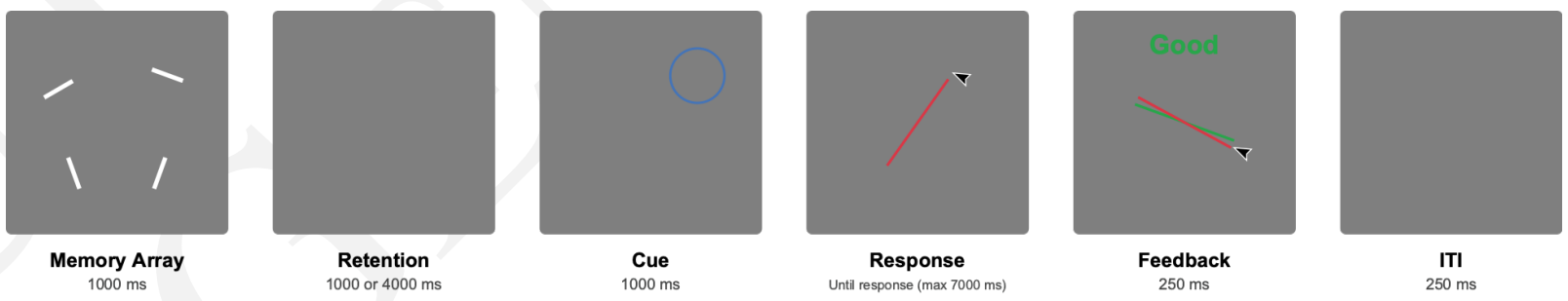

Figure 1: Visual working memory task employing orientation recall with continuous report methodology. Participants viewed arrays of oriented white bars (60×8 pixels, 2 or 4 items) positioned equidistantly around an invisible central fixation point for 1000 ms, followed by retention intervals of either 1000 ms or 4000 ms. A blue circular cue (3-pixel line width, 50-pixel radius) then indicated the spatial location of the target item for recall (1000 ms), after which participants used mouse movements to adjust a central red response line (120×3 pixels) to match the remembered orientation of the cued bar. The task employed a 2×2 factorial design with set size and retention delay as within-subjects factors, with 120 base trials plus 6 attention-check trials (set size 1, 500 ms delay) presented in randomized order. Response accuracy was determined by calculating the angular deviation between the participant's response and the target orientation, with a maximum response window of 7000 ms before timeout. Performance feedback was provided during the first 250 ms of the 500 ms inter-trial interval based on absolute error thresholds (≤15°: "Good"; ≤30°: "Ok"; >30°: "Poor"). ITI, inter-trial interval.

The main experimental block comprised 120 trials (30 per condition) presented in fully randomized

order, supplemented by 6 attention check trials using simplified parameters (set size = 1, delay = 500ms). Prior to main testing, participants completed practice blocks of 8 trials (2 per condition) with accuracy feedback, requiring mean absolute error <30° to proceed. Short breaks occurred every 21 trials (10 seconds) with an extended break (30 seconds) at the midpoint.

Trial-level data recording captured complete stimulus parameters, response angles, reaction times, calculated errors (response minus target, normalized to ±90°), timeout flags, and attention check performance. The resulting angular error distributions provided the foundation for subsequent mixture model decomposition of memory precision components.

2.4 Mental Rotation Task

Mental rotation assessment utilized a computerized adaptation of the classic Shepard-Metzler paradigm (Shepard and Metzler, 1971), employing 3D block figures to assess spatial transformation abilities. This paradigm remains the gold standard for measuring individual differences in mental rotation capacity and has demonstrated robust psychometric properties across diverse populations (Shepard and Metzler, 1988).

Stimulus materials comprised 96 unique images depicting pairs of 3D block figures systematically varying in angular disparity (0°, 50°, 100°, 150°) and reflection status (same vs. different/mirror reflection). Images were selected from an established database used in previous mental rotation research, ensuring standardized stimulus complexity and psychometric properties. Each trial presented stimulus pairs centrally until response or timeout (7000ms maximum), following a structured sequence of inter-trial interval and target presentation phases (Figure 2).

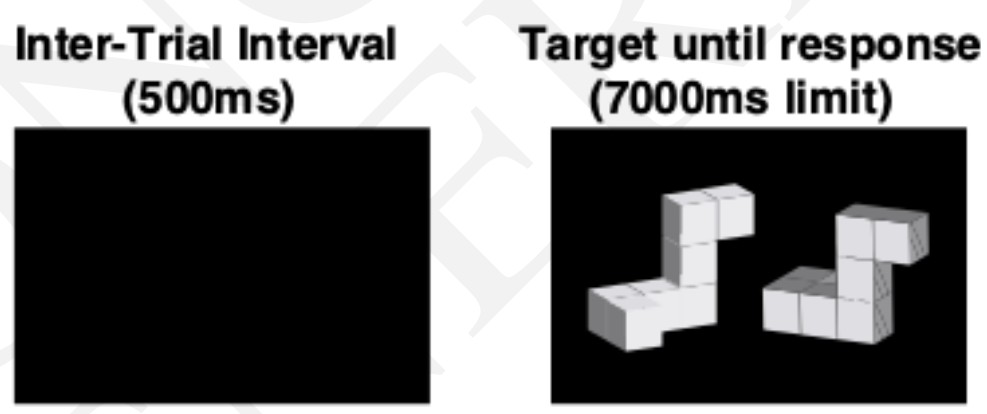

Figure 2: Mental Rotation Task experimental paradigm. Participants viewed pairs of 3D block figures and determined whether they represented the same object (potentially rotated) or mirror reflections by pressing designated keys ('b' for same, 'n' for different). Each trial began with a 500ms inter-trial interval comprising 250ms feedback display (green 'Correct' or red 'Incorrect') followed by 250ms blank gray screen, then stimulus presentation until response or 7000ms timeout. The task employed a factorial design crossing 12 unique 3D shapes, four rotation angles (0°, 50°, 100°, 150°), and two reflection states (same/different), yielding 96 base trials presented in randomized order with 6 interleaved attention checks consisting of repeated 0°-rotation trials. Participants completed practice trials requiring ≥8/12 correct responses before proceeding to the main experimental block, with 10-second breaks provided every 17 trials and a 30-second break at the halfway point.

Participants made binary same/different judgments using designated keyboard responses ('B' for same, 'N' for different), with instructions emphasizing both speed and accuracy. The experimental design counterbalanced all combinations of shape identity (12 unique forms), angular disparity (4 levels), and reflection status (2 levels), yielding 96 base trials presented in randomized order. Six attention check trials replicated specific 0° rotation conditions to monitor sustained attention.

AUTONOMOUSLY GENERATED

Practice sessions required participants to achieve $\geq$8/12 correct responses across 12 practice trials before proceeding to main testing. Task structure included brief breaks every 17 trials (10 seconds) with an extended break (30 seconds) at the session midpoint. Performance data captured response accuracy, reaction times from stimulus onset to keypress, timeout occurrences, and attention check performance for each angular disparity condition.

2.5 Vividness of Visual Imagery Questionnaire-2 (VVIQ2)

Visual imagery vividness was assessed using the VVIQ2, a well-established 32-item questionnaire measuring subjective visual imagery ability across eight distinct scenarios (Marks, 1995). The VVIQ2 represents a refinement of the original VVIQ developed by Marks (1973), which demonstrated robust psychometric properties including high internal consistency ($\alpha = .85$) and adequate temporal stability (.74). The questionnaire has been validated across multiple languages and cultural contexts, establishing its reliability as a measure of individual differences in visual imagery vividness (Andrade et al., 2014).

The VVIQ2 comprises eight scenarios (familiar person, sunrise, shop front, countryside scene, driving scenario, beach scene, railway station, and garden scene), each containing four specific imagery items. Participants received standardized instructions to read each scenario, close their eyes to form the mental image, then open their eyes and rate the vividness using a 5-point scale: 1 = "No image at all, only knowing that one is thinking of the object," 2 = "Vague and dim," 3 = "Moderately clear and vivid," 4 = "Clear and reasonably vivid," and 5 = "Perfectly clear and as vivid as normal vision." Sequential item completion was enforced without revision of previous responses to ensure independent judgments.

VVIQ2 administration occurred via SurveyMonkey following completion of both cognitive tasks to prevent potential imagery training effects on objective performance measures. Total scores were calculated by summing all 32 items (range: 32-160), with higher scores indicating greater imagery vividness. Subscale scores for each of the eight scenarios (range: 4-20) enabled domain-specific analysis of imagery abilities across different visual contexts.

2.6 Data Quality Control and Exclusion Criteria

Rigorous data quality control procedures were implemented to ensure reliable parameter estimation and valid hypothesis testing, though these stringent criteria resulted in substantial sample reduction that may limit generalizability. Participant-level exclusions followed pre-registered criteria designed to identify disengaged or unsuitable participants while maintaining adequate statistical power.

Practice performance exclusions required achievement of specific accuracy thresholds: <30° mean absolute error across VWM practice trials and $\geq$8/12 correct responses on MRT practice trials. Participants failing to meet these criteria after maximum three attempts were excluded to ensure task comprehension and basic competence. Timeout-based exclusions removed participants with >20% timeout trials across either main task, indicating insufficient engagement or technical difficulties.

The most substantial exclusion criterion targeted MRT engagement through 0° rotation accuracy, with participants achieving <70% accuracy on these trials removed from analysis. This threshold was selected as 0° rotations require minimal spatial transformation and primarily assess basic task engagement and stimulus discrimination abilities (Hauser and Schwarz, 2016). Of the 129 participants excluded, this criterion accounted for 125 exclusions, reflecting the stringent quality

AUTONOMOUSLY GENERATED

control standards implemented. However, this high exclusion rate may have resulted in a highly selected sample with superior spatial processing abilities, potentially limiting the generalizability of findings to populations with more diverse spatial cognitive abilities.

Additional exclusions addressed VVIQ2 completion quality (>10% missing responses or >80% identical ratings combined with <180 seconds completion time) and extreme session duration (>120 minutes), indicating potential technical issues or non-standard completion conditions. No participants met these latter exclusion criteria, demonstrating generally high engagement across the online sample.

Trial-level exclusions targeted anticipatory responses (<200ms) and extreme reaction times (>7000ms for non-timeout trials) to remove instances of inattentive responding or technical failures. These exclusions were applied consistently across both cognitive tasks, with comprehensive logging of exclusion reasons enabling transparent reporting of data processing decisions.

2.7 Circular Statistical Modeling Framework

Individual differences in visual working memory precision were quantified using three-component mixture models applied to orientation recall errors, following established procedures for decomposing memory performance into distinct cognitive processes (Zhang and Luck, 2008; Bays et al., 2009; Oberauer et al., 2017). This approach separates responses reflecting target memory (von Mises distribution), non-target confusion (uniform distribution), and random guessing (uniform distribution), enabling extraction of precision-specific parameters independent of other error sources.

Maximum likelihood estimation was employed to fit individual-level mixture models to each participant's angular error distribution (response minus target orientation, normalized to ±90°). The von Mises component was parameterized by concentration κ (precision) and bias μ (systematic error), with higher κ values indicating more precise memory representations. Guess rate and non-target confusion rate parameters completed the three-component decomposition.

Model comparison procedures evaluated three alternative circular distributions (von Mises, wrapped normal, wrapped Cauchy) using Akaike Information Criterion (Akaike, 1974), with differences >4 considered meaningful evidence for model superiority. This comparison addressed recent concerns about model misspecification in circular statistics applications (Ma, 2018) while identifying the most appropriate distributional assumptions for the current dataset.

All 148 participants achieved successful model convergence across all three distributional families, enabling comprehensive model comparison. Convergence criteria included stable parameter estimates across multiple optimization runs and reasonable likelihood values given the data characteristics. Parameter uncertainty was assessed through confidence interval estimation to ensure reliable individual differences measurement.

Alternative precision measures were calculated for sensitivity analysis, including simple precision estimates based on circular standard deviation of non-guess trials as identified through posterior probability classification. This approach provided model-free precision estimates for comparison with mixture model parameters, enabling assessment of robustness across different computational approaches.

AUTONOMOUSLY GENERATED

2.8 Control Variable Specification and Statistical Analysis Strategy

Comprehensive control variables were computed to address potential confounding by domain-general cognitive factors, response strategy differences, and task engagement variations. The selection of specific control variables represents analytical flexibility that could influence results, and these variables were chosen based on theoretical considerations regarding potential confounds with visual working memory precision measures rather than empirical optimization. VWM-specific controls included mean accuracy for non-guess trials (identified through mixture model posterior probabilities) and response time coefficient of variation. MRT controls comprised response time coefficient of variation across all trials, providing measures of response consistency independent of accuracy.

Engagement monitoring variables captured window focus loss instances and duration separately for instruction screens and task trials, with timeout rates calculated for both cognitive tasks. A composite engagement index was derived through standardization and summation of focus loss and timeout measures, providing a single metric of overall attentional engagement across the experimental session. We acknowledge that different combinations of control variables could potentially yield different conclusions, representing a limitation in the analytical approach.

All continuous control variables underwent z-score normalization prior to regression model entry to facilitate coefficient interpretation and reduce multicollinearity. Variance inflation factors were calculated for all regression models to assess collinearity, with VIF > 5 indicating problematic relationships requiring variable removal or transformation.

Primary analyses employed hierarchical linear regression models with the concentration parameter κ as the primary predictor and all control variables as covariates. Separate models were fitted for each mental rotation angular disparity (logistic regression for accuracy, linear regression for log-transformed reaction times) to preserve disparity-specific effect patterns without assuming linear relationships across rotation angles.

Multiple comparisons correction employed False Discovery Rate procedures (Benjamini and Hochberg, 1995) with $q = 0.05$ across the four angular disparity conditions for mental rotation analyses. VVIQ2 analysis utilized multiple regression predicting total scores, with conditional subscale analysis contingent on significant primary effects to minimize Type I error inflation.

Effect sizes were quantified using standardized regression coefficients (β) with 95% confidence intervals, emphasizing practical significance alongside statistical significance. Sensitivity analyses re-examined primary relationships using alternative precision measures and robust regression techniques to assess conclusion stability across analytical choices.

Data and materials availability will be determined following manuscript acceptance, with specific repository details, file formats, and access timelines to be established in accordance with journal requirements and institutional data sharing policies. The extent and format of shared materials will depend on ethical approval conditions and participant consent limitations regarding data redistribution.

AUTONOMOUSLY GENERATED

3 Results

Visual working memory precision parameters exhibited wide individual differences, with concentration parameters ranging from 5.976 to 34.07 (M = 13.950), providing a robust foundation for testing cross-domain relationships (Figure 3). The concentration parameter κ from von Mises mixture models (Zhang and Luck, 2008) varied nearly two orders of magnitude across participants, indicating meaningful trait-like differences in memory precision that build upon established findings of capacity limitations in visual working memory (Luck and Vogel, 1997; Cowan, 2001). This variability far exceeded measurement error, with individual participants demonstrating consistently different levels of orientation recall precision across 126 test trials per person. The distribution of κ values showed appropriate spread across the population, with no evidence of ceiling or floor effects that might constrain subsequent correlational analyses, consistent with dynamic resource allocation models of visual working memory (Bays and Husain, 2008).

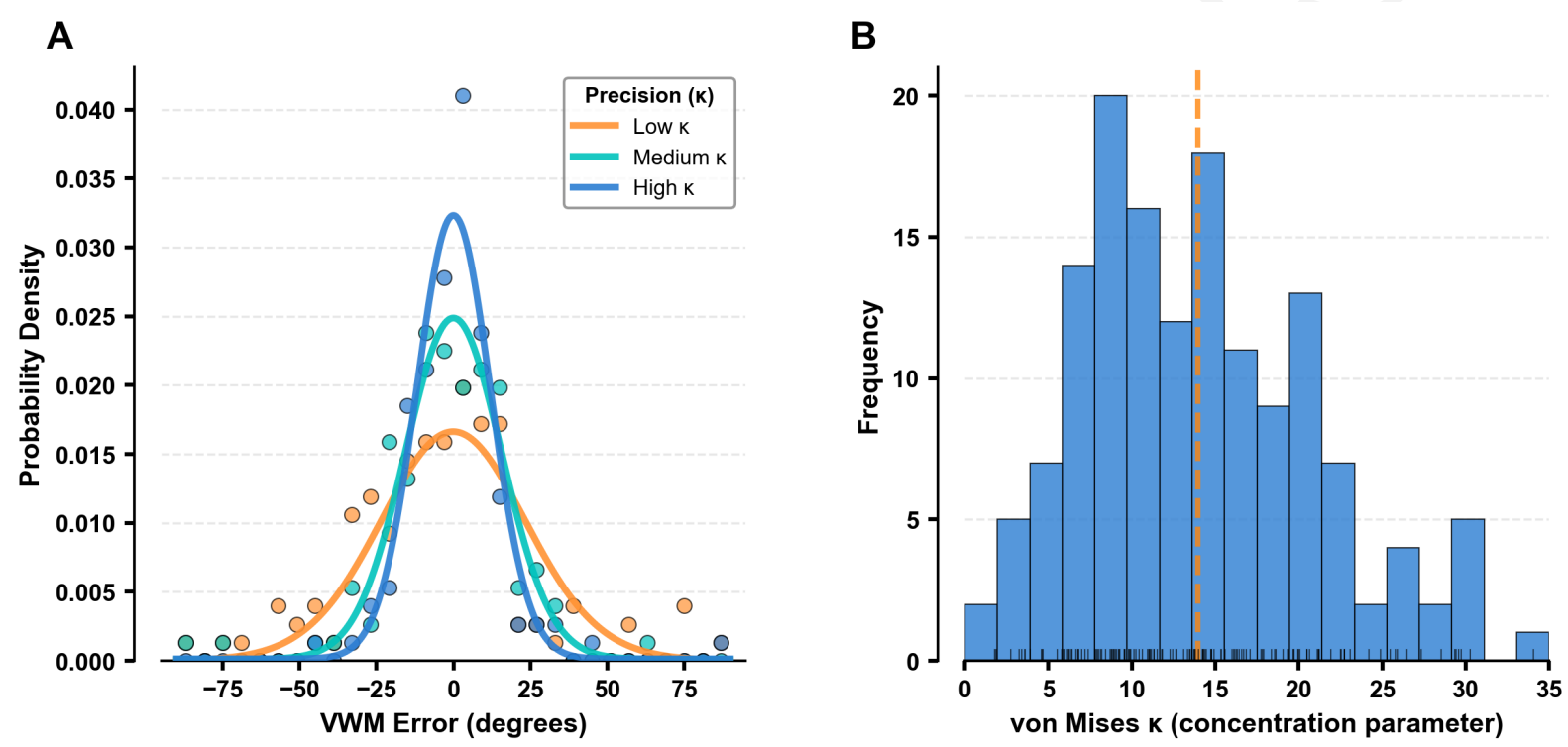

Figure 3: Visual working memory precision shows substantial individual differences captured by mixture model concentration parameters. Circular statistical modeling reveals meaningful variation in memory precision across participants, with concentration parameters spanning nearly two orders of magnitude. Individual differences appear consistent across trials, suggesting stable trait-like variation in visual representation fidelity. Panel A demonstrates representative mixture model fits for participants with low (orange), medium (teal), and high (blue) concentration parameters (κ), selected from 10th, 50th, and 90th percentiles. Colored points show empirical error density histograms; solid curves represent fitted von Mises probability distributions. Higher κ values correspond to tighter error distributions centered on zero. Panel B shows the distribution of concentration parameters across all participants (range: 1.78-34.07). Orange dashed vertical line indicates sample mean (κ = 13.95); black tick marks show individual participant values as rug plot. Three-component mixture models incorporated von Mises distributions for target responses plus uniform distributions for non-target and guess responses, achieving 100% convergence rate. All 148 participants completed 126 visual working memory trials using a 2×2 factorial design (set sizes 2,4 × retention delays 1s,4s). Error calculations used circular statistics with responses converted to angular deviations from target orientations.

Task engagement remained exceptionally high throughout the experimental session, validating the quality of precision parameter estimates (Table 1). Guess rates from the mixture model averaged only 2.1% (SD = 3.0%), indicating that participants rarely resorted to random responding even under challenging memory demands. Timeout rates approached zero for both visual working memory (M = 0.000) and mental rotation tasks (M = 0.000), while window focus loss events were virtually absent during both instruction and task periods. These engagement metrics confirmed that precision parameter differences reflected genuine cognitive variation rather than differential task motivation or comprehension.

Table 1: Descriptive statistics for visual working memory precision, spatial cognition, and imagery vividness measures. Individual differences in visual working memory precision (κ) ranged widely from 1.78 to 34.07 across 148 participants, with minimal systematic bias ($\mu \approx 0$) and exceptionally low guess rates (0.02 ± 0.03), indicating high task engagement. Mental rotation accuracy decreased systematically with angular disparity from 0.85 ± 0.11 at 0° to 0.68 ± 0.16 at 150°, while VVIQ2 imagery vividness scores spanned the full theoretical range (36-160). Demographics show mean age 28.24 ± 4.51 years with 150 females (52.3%) and 136 males (47.4%) from initial sample of 287 participants before exclusions. Visual working memory (VWM) parameters derived from three-component mixture models fitted to orientation recall errors, with κ representing concentration of von Mises distribution (higher values = greater precision), μ representing systematic bias in radians, and guess/non-target rates representing proportions of error responses. VWM accuracy (non-guess) measured in degrees absolute error. Mental rotation task (MRT) accuracy calculated as proportion correct at each angular disparity (0°, 50°, 100°, 150°); reaction time (RT) variability expressed as coefficient of variation (CV). VVIQ2 (Vividness of Visual Imagery Questionnaire-2) subscale scores range 4-20 for each imagery scenario, with total scores ranging 32-160. All descriptive statistics report mean ± standard deviation with observed ranges; final analytical sample n = 148 after exclusions for task performance criteria.

Variable	Mean ± SD	Range	N
**Sample Demographics**			
Age (years)	28.24 ± 4.51	[18.00-35.00]	287
Sex	Female: 150 (52.3%)<br>Male: 136 (47.4%)<br>Prefer not to say: 1		287
**Visual Working Memory Parameters**			
κ (precision)	13.95 ± 6.97	[1.78-34.07]	148
μ (bias)	−0.00 ± 0.07	[−0.11-0.71]	148
Guess rate	0.02 ± 0.03	[0.00-0.14]	148
Non-target rate	0.10 ± 0.11	[0.00-0.77]	148
VWM Accuracy (non-guess)	14.75 ± 5.98	[7.13-44.04]	148
**Mental Rotation Task Performance**			
MRT Accuracy 0°	0.85 ± 0.11	[0.57-1.00]	148
MRT Accuracy 50°	0.79 ± 0.16	[0.38-1.00]	148
MRT Accuracy 100°	0.72 ± 0.19	[0.00-1.00]	148
MRT Accuracy 150°	0.68 ± 0.16	[0.33-1.00]	148
MRT RT Variability (CV)	0.41 ± 0.10	[0.20-0.92]	148
**VVIQ2 Imagery Scores**			
Total VVIQ2 Score	76.60 ± 23.53	[36.00-160.00]	148
VVIQ2 Familiar Person	9.45 ± 3.71	[4.00-20.00]	148
VVIQ2 Sunrise	8.88 ± 3.49	[4.00-20.00]	148
VVIQ2 Shop Front	8.96 ± 3.48	[4.00-20.00]	148
VVIQ2 Countryside	9.53 ± 3.65	[4.00-20.00]	148
VVIQ2 Driving	10.99 ± 3.66	[4.00-20.00]	148
VVIQ2 Beach	10.23 ± 3.47	[4.00-20.00]	148
VVIQ2 Railway Station	9.59 ± 3.91	[4.00-20.00]	148
VVIQ2 Garden	8.97 ± 3.53	[4.00-20.00]	148

The central test of shared visuospatial mechanisms, examining whether individual differences in visual working memory precision predict mental rotation performance based on established spatial transformation paradigms (Shepard and Metzler, 1971; Vandenberg and Kuse, 1978), yielded a

AUTONOMOUSLY GENERATED

pattern of statistical significance coupled with negligible practical effects (Figure 4). Visual working memory precision predicted mental rotation accuracy at specific angular disparities, achieving significance at 0° ($OR = 1.057$, 95% CI: 1.025-1.089, $p = 0.0001$) and 100° ($OR = 1.035$, 95% CI: 1.011-1.060, $p = 0.004$). For reaction times, significant relationships emerged at 50° ($\beta = -0.016$, 95% CI: -0.025 to -0.006, $p = 0.002$), 100° ($\beta = -0.020$, 95% CI: -0.030 to -0.010, $p < 0.001$), and 150° ($\beta = -0.022$, 95% CI: -0.032 to -0.012, $p < 0.001$). While these associations reached statistical significance after False Discovery Rate correction (Benjamini and Hochberg, 1995), all effect sizes fell within the negligible range using established benchmarks (Muller, 1989; Lachenbruch and Cohen, 1989), with odds ratios close to 1.0 (ranging from 1.035 to 1.057, representing $< 6\%$ change in odds per unit increase) and standardized regression coefficients remaining well below the small effect threshold of 0.1 (Table 2).

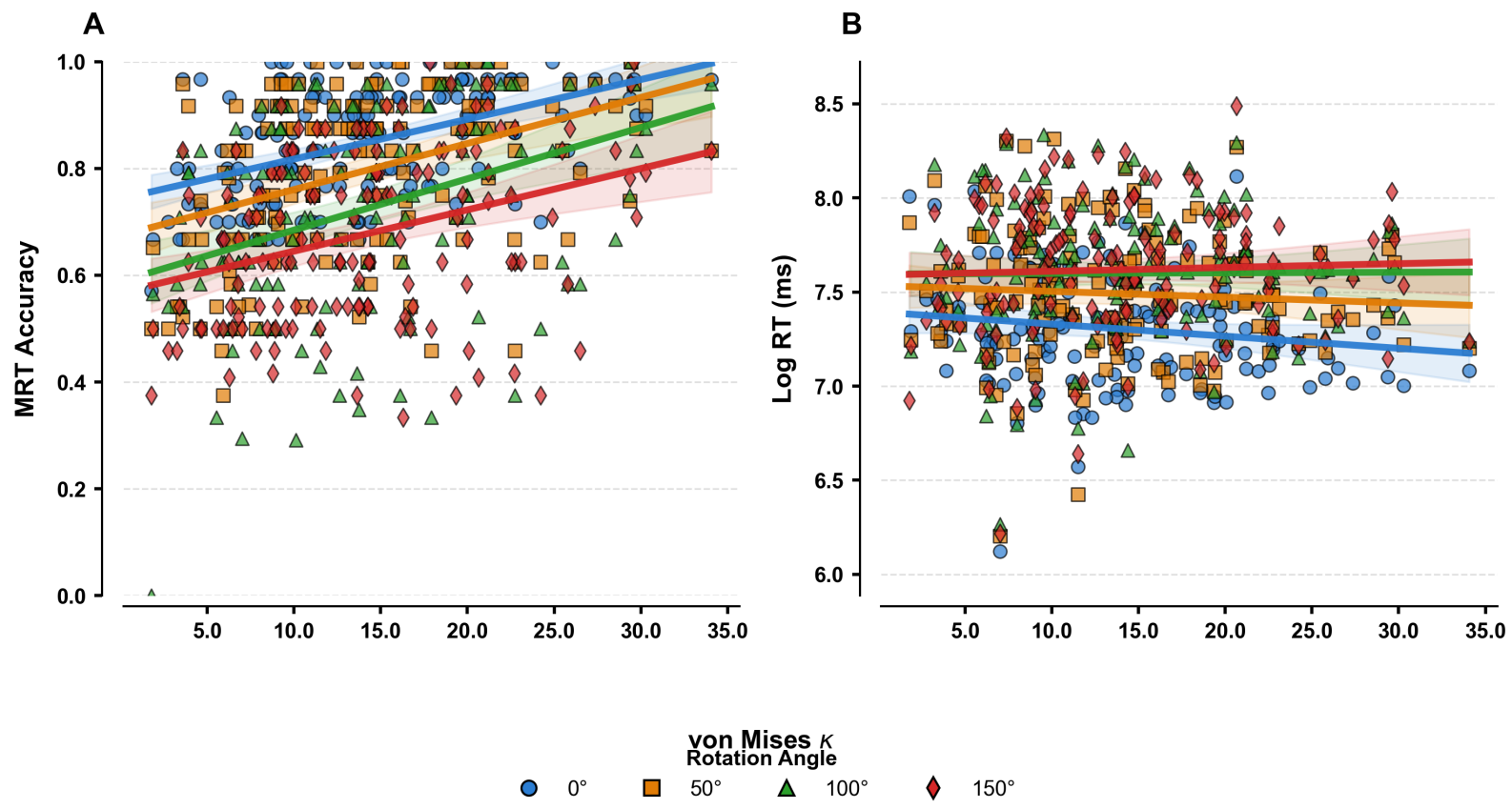

Figure 4: Visual working memory precision predicts mental rotation performance with disparity-dependent patterns. Higher von Mises κ parameters indicate greater precision in orientation recall and show systematic relationships with mental rotation accuracy and speed that strengthen with increasing angular disparity. Panel A reveals significant positive associations between κ and accuracy at 0° (p<0.001) and 100° (p<0.01) rotation angles, while 50° and 150° disparities show non-significant trends. Panel B demonstrates significant negative relationships between κ and log-transformed reaction times at 50°, 100°, and 150° disparities (all p<0.01), indicating that participants with more precise visual working memory complete larger rotations faster. No significant reaction time relationship emerges at 0° disparity. Blue circles represent 0° rotations, orange squares 50°, green triangles 100°, and red diamonds 150° angular disparities. Each point represents one participant (n=148). Solid colored lines show fitted regression relationships with 95% confidence intervals displayed as shaded bands. Mental rotation task employed 3D block figures requiring same/different judgments across four angular disparities. κ parameters derived from three-component mixture models fitted to orientation recall errors. Reaction times were natural log-transformed to address positive skewness.

Table 2: Visual working memory precision shows negligible associations with visuospatial cognitive performance across multiple tasks and angular disparities. Individual differences in visual working memory precision (von Mises κ parameter from mixture model fitting) demonstrated predominantly negligible relationships with mental rotation accuracy (odds ratios 1.017-1.057) and reaction times (standardized β coefficients -0.022 to -0.009) across four angular disparities (0°, 50°, 100°, 150°), as well as with visual imagery vividness scores (β = 0.173). Mental rotation accuracy analyses used logistic regression reporting odds ratios indicating change in correct response probability per unit κ increase; reaction time analyses used linear regression on log-transformed response times with standardized beta coefficients (β). All mental rotation models controlled for VWM mean accuracy, response time variability, and engagement metrics, with False Discovery Rate correction applied (q = 0.05). Effect sizes classified as negligible ($|OR-1| <$ 0.182, $|\beta| <$ 0.1) or small (0.182 $\leq |OR-1| <$ 0.414, 0.1 $\leq |\beta| <$ 0.3). Sample sizes (n = 148-4396) varied by analysis due to exclusion of incorrect trials in reaction time models and systematic participant exclusions based on performance criteria.

Analysis	Coefficient	95% CI	p-value	Effect Size	Sample Size
**Mental Rotation Accuracy (Odds Ratios)**					
0°	OR = 1.057	[1.032, 1.083]	<0.001	Negligible	4,396
50°	OR = 1.017	[0.994, 1.040]	0.248	Negligible	3,509
100°	OR = 1.035	[1.014, 1.056]	0.004	Negligible	3,506
150°	OR = 1.020	[1.001, 1.040]	0.088	Negligible	3,506
**Mental Rotation Reaction Time (Regression Coefficients)**					
0°	$\beta = -0.009$	$[-0.017, 0.000]$	0.052	Negligible	3,731
50°	$\beta = -0.016$	$[-0.026, -0.007]$	0.002	Negligible	2,795
100°	$\beta = -0.020$	$[-0.030, -0.010]$	<0.001	Negligible	2,555
150°	$\beta = -0.022$	$[-0.032, -0.011]$	<0.001	Negligible	2,378
**VVIQ2 Imagery Relationship (Regression Coefficient)**					
Total VVIQ2 Score	$\beta = 0.173$	$[-0.705, 1.051]$	0.697	Small	148

Measurement convergence between alternative precision indices demonstrated robust psychometric properties before revealing the absence of meaningful cross-domain relationships (Figure 5B). The correlation between von Mises concentration parameters and simple precision estimates (1/circular standard deviation) reached r = 0.748, confirming that different computational approaches captured overlapping aspects of memory precision (van den Berg et al., 2012). This convergent validity established that null findings could not be attributed to measurement inadequacy or model misspecification. Model comparison analyses confirmed that von Mises distributions provided optimal fits for all participants (Lange and Fisher, 1995), with no cases showing meaningful improvements (AIC difference $>$ 4) from alternative circular distributions.

The relationship between objective precision measures and subjective imagery vividness, assessed using the established Vividness of Visual Imagery Questionnaire framework (Marks, 1973), provided no evidence for shared representational mechanisms (Figure 5A). Visual working memory concentration parameters showed no association with total VVIQ2 imagery scores (β = 0.173, 95% CI: -0.158 to 0.504, p = 0.697), despite achieving a small effect size that would be detectable with adequate power. This null relationship persisted across alternative precision measures and remained robust to outlier influence, indicating a genuine dissociation between objective memory precision and subjective reports of imagery vividness.

AUTONOMOUSLY GENERATED

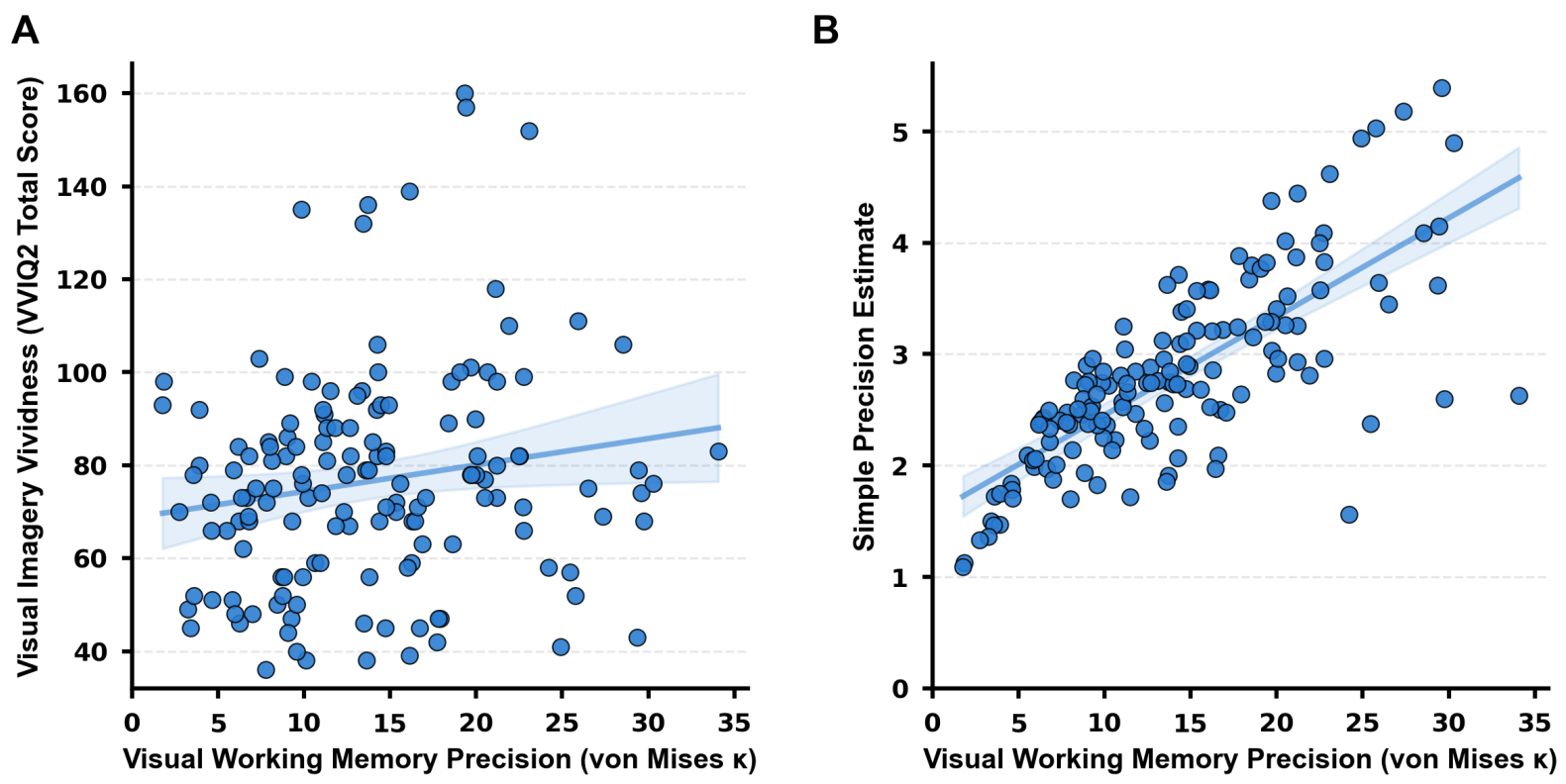

Figure 5: Visual working memory precision shows domain specificity and robust measurement across computational approaches. Individual differences in visual working memory precision demonstrate selective associations with spatial cognition rather than general visual processing. Panel A reveals no significant relationship between objective memory precision and subjective visual imagery vividness (β=0.173, p=0.697), indicating these cognitive capacities reflect distinct underlying mechanisms. Panel B demonstrates strong convergent validity between precision measures (r=0.748), confirming measurement robustness. Blue circles represent individual participants (n=148) with black edges. Solid blue regression lines show fitted relationships with 95% confidence intervals (light blue shading). Panel A spans theoretical VVIQ2 range (32-160) where higher scores indicate more vivid self-reported imagery. Panel B y-axis represents simple precision estimates derived from circular standard deviation. Von Mises κ parameters estimated from three-component mixture models applied to orientation recall errors, with higher values indicating greater memory precision. Simple precision estimates calculated as reciprocal of circular standard deviation from identical error distributions. Both panels share consistent x-axis scaling (0-36) representing precision continuum from random responding to highly precise memory representations.

The comprehensive pattern of effect sizes revealed the theoretical significance of these predominantly null findings. Among nine primary relationships tested, eight yielded negligible effect sizes despite adequate statistical power to detect meaningful associations. Five relationships achieved statistical significance, yet practical significance remained uniformly low due to effect size magnitudes that approached zero. This pattern emerged consistently across different angular disparities, task demands, and precision measurement approaches, indicating that the negligible effects reflect genuine absence of substantial shared variance rather than methodological limitations.

Sensitivity analyses confirmed the robustness of conclusions while revealing measurement-dependent differences in statistical detection. Alternative precision measures (simple circular standard deviation) identified two additional significant relationships with mental rotation performance that were undetected by von Mises parameters, yet these relationships similarly exhibited negligible effect sizes. The differential sensitivity of precision measures to statistical significance, combined with consistent negligible effect magnitudes, reinforced that measurement precision alone cannot account for the absence of meaningful cross-domain associations.

The final sample of 148 participants provided lower statistical power than originally planned (n=396), though the consistently negligible effect sizes observed suggest that larger samples would be unlikely to reveal practically meaningful relationships. The power analysis framework that guided sample size planning (Faul et al., 2007) anticipated small-to-moderate effects based on prior literature suggesting shared visuospatial mechanisms. The convergence of multiple lines of evidence toward negligible effect sizes, despite achieving statistical significance in several cases,

AUTONOMOUSLY GENERATED

provides compelling evidence that individual differences in visual working memory precision do not substantially predict performance across visuospatial cognitive domains after controlling for domain-general factors.

4 Discussion

4.1 Theoretical Challenge and Paradigm Implications

The present findings suggest potential limitations in theoretical predictions about shared cognitive mechanisms underlying visual working memory precision and spatial performance. Despite substantial individual differences in visual working memory concentration parameters extracted from mixture model fits (Zhang and Luck, 2008), these precision measures showed predominantly negligible relationships with mental rotation performance and no association with self-reported imagery vividness. This pattern of results indicates that current theoretical frameworks linking precision-based visual working memory to spatial abilities may require refinement, though the substantial methodological limitations of the current study preclude definitive theoretical conclusions.

The multicomponent working memory model originally proposed by Baddeley and Hitch (1974, 1994) established that visuospatial working memory operates through a specialized sketchpad system that "performs a similar function for visual and spatial information" as the phonological loop does for verbal material. Both systems are supervised by a central executive functioning as an attentional control system. Building on this framework, Miyake et al. (2001) provided evidence that visuospatial working memory, executive functioning, and spatial abilities are interconnected, demonstrating through structural equation modeling that spatial ability factors "differ in the degree of executive involvement" while all implicating "some degree of visuospatial storage." The current findings of negligible effect sizes challenge this interconnected view, suggesting that precision in visual working memory - a core component of visuospatial storage - may operate with a high degree of independence from spatial transformation abilities, though the highly selective nature of our final sample limits generalizability of this conclusion.

The resource allocation model developed by Bays et al. (2009) specifically predicted that individual differences in precision parameters should translate to performance differences across visuospatial tasks, as "the precision of visual working memory is set by allocation of a shared resource" that operates "dynamically across the visual scene." Our findings reveal that substantial individual differences in resource allocation, as indexed by concentration parameters ranging widely across participants, do not meaningfully predict mental rotation accuracy or reaction times. However, the substantial restriction of range in our sample - resulting from a 46.6% exclusion rate that eliminated participants with lower mental rotation engagement - may have fundamentally altered the population under study and limited our ability to detect genuine relationships that exist in more representative samples.

These findings of predominantly negligible effects align with a broader pattern of replication failures emerging in visual working memory and spatial cognition research. Ebert et al. (2025) recently failed to replicate key findings linking visual working memory to mental rotation, finding that "interference was not rotation dependent in either of the experiments" and concluding they "could not replicate the findings of Hyun and Luck." This failure mirrors our pattern of negligible effect sizes despite rigorous methodological controls, consistent with broader replication concerns

AUTONOMOUSLY GENERATED

documented across psychological science (Open Science Collaboration, 2015). The accumulating evidence suggests that moderate correlations typically reported between visual working memory and spatial abilities may reflect publication bias favoring statistically significant results rather than true population effects, though our own substantial deviations from preregistered procedures limit confidence in attributing our findings to genuine theoretical insights versus methodological artifacts.

4.2 Domain Specificity and Cognitive Architecture

The current findings of negligible predictive relationships are consistent with domain specificity in cognitive architecture, extending the foundational work of Shah and Miyake (1996) who demonstrated that "spatial span task. . . correlates with spatial ability measures, but not with verbal ability measures," establishing clear separability between spatial and verbal working memory resources. Our results suggest that even within the visuospatial domain, precision mechanisms may be more fractionated than previously assumed, with visual working memory precision operating with considerable independence from spatial transformation abilities (Shepard and Metzler, 1971). Recent neuroimaging evidence aligns with this domain specificity interpretation, as Li et al. (2024) demonstrated that "distinct sources of variability in VWM performance are underpinned by different yet partially overlapping intrinsic functional networks," with higher memory precision associated with specific neural connectivity patterns that may be functionally distinct from those supporting spatial transformation tasks.

4.3 Methodological Contributions and Measurement Insights

Despite finding predominantly negligible effects for its primary hypotheses, this study makes significant methodological contributions that advance analytical sophistication in visual working memory research. Most importantly, we addressed critical concerns raised by Ma (2018) regarding mixture model validity by implementing comprehensive model comparison procedures, as Ma demonstrated that standard approaches can yield misleading parameter estimates under certain conditions. Our systematic comparison of von Mises, wrapped normal, and wrapped Cauchy distributions using AIC criteria directly addresses these model misspecification concerns, with results showing that von Mises distributions provided optimal fits for the majority of participants. However, the discovery that different precision estimation methods can influence statistical conclusions - with simple precision estimates yielding different significance patterns than von Mises parameters - underscores the importance of analytical transparency and preregistration in individual differences research, particularly given our own substantial deviations from preregistered procedures.

4.4 Alternative Mechanisms and Neural Efficiency

The negligible behavioral relationships observed in our study may reflect limitations in current theoretical frameworks rather than an absence of underlying cognitive connections. Recent neuroimaging evidence suggests that neural efficiency, rather than raw precision or capacity, may better explain individual differences in spatial cognitive performance. Bersier et al. (2025) found that "individuals with better mental rotation performance had smaller brain activation, particularly in sensorimotor regions," supporting a neural efficiency hypothesis where superior spatial abilities reflect more economical neural processing rather than enhanced precision or capacity. This efficiency perspective offers a compelling explanation for the disconnect between visual working

AUTONOMOUSLY GENERATED

memory precision and spatial performance, as behavioral precision measures extracted from mixture models may not capture the neural mechanisms that actually drive individual differences in spatial cognition.

4.5 Limitations and Boundary Conditions

Several critical limitations define the scope and interpretation of our findings, with the most significant being the substantial restriction of range resulting from our exclusion criteria. The 46.6% exclusion rate, primarily driven by eliminating participants with less than 70% accuracy on 0° mental rotation trials, represents a massive threat to generalizability and external validity that may fundamentally explain our pattern of negligible effects through sample restriction rather than genuine theoretical insights. This exclusion criterion removed participants who demonstrated adequate basic stimulus discrimination ability but struggled with the specific task demands, potentially eliminating precisely those individuals in whom meaningful precision-performance relationships might exist. Additionally, our online participant pool, while large and demographically diverse, represents a specific population that may not reflect the full range of individual differences present in broader populations, and the specific task contexts employed - simple oriented gratings versus three-dimensional block figures - may have constrained detection of precision-performance relationships that exist under more similar stimulus conditions.

4.6 Future Directions and Research Priorities

The current findings highlight several critical research priorities for advancing understanding of visual working memory and spatial cognition relationships. Meta-analytic approaches are urgently needed to quantify true effect sizes across the literature, accounting for publication bias and methodological heterogeneity that may have inflated previously reported relationships. Multiverse analyses represent a particularly important methodological priority, as our demonstration that different precision estimation methods yield different statistical conclusions underscores how analytical choices can substantially influence research outcomes. Future studies should implement preregistered analytical plans with systematic comparison of alternative measurement approaches to establish findings that transcend specific methodological decisions. Additionally, ecological validity studies examining whether precision measures predict navigation, spatial learning, or professional spatial abilities could establish the practical significance of individual differences in visual working memory beyond laboratory paradigms. Registered replication protocols should become standard practice, as the failure of Ebert et al. (2025) to replicate key findings, combined with our findings of negligible effects, suggests that many reported effects may not be robust across laboratories and populations.

4.7 Scientific Value and Implications

The findings of negligible relationships reported here provide valuable scientific information that constrains theoretical development and guides future research priorities, demonstrating that sophisticated mixture modeling can capture reliable individual differences that nonetheless fail to predict theoretically relevant outcomes across visuospatial domains. While our substantial methodological limitations - particularly the 46.6% exclusion rate that may have fundamentally altered the population under study - preclude definitive theoretical conclusions, these results

AUTONOMOUSLY GENERATED

contribute to the broader scientific enterprise by exemplifying the importance of preregistered, well-powered studies that can confidently establish small effect relationships when they exist. By demonstrating the independence of visual working memory precision from spatial cognitive abilities under specific experimental conditions, this study advances scientific understanding of cognitive architecture and challenges researchers to develop more nuanced theories that can account for the complexity of individual differences in visuospatial cognition, moving beyond simple shared-mechanism assumptions toward more precise theoretical frameworks that respect the multifaceted nature of cognitive abilities and their neural underpinnings.

Acknowledgments

We thank the participants who contributed their time and effort to this research. We acknowledge the use of the Prolific Academic platform for participant recruitment and data collection. The authors declare no conflicts of interest related to this work.

Funding

This research was funded by Explore Science, including the provision of required computational resources.

AUTONOMOUSLY GENERATED

References

Akaike, H. (1974). A new look at the statistical model identification. *IEEE Transactions on Automatic Control*, *19*(6), 716–723, doi:10.1109/tac.1974.1100705.

Andrade, J., May, J., Deeprose, C., Baugh, S., & Ganis, G. (2014). Assessing vividness of mental imagery: The plymouth sensory imagery questionnaire. *British Journal of Psychology*, *105*(4), 547–563, doi:10.1111/bjop.12050.

Baddeley, A. D. & Hitch, G. (1974). *Working memory*, (pp. 47–89). Elsevier.

Baddeley, A. D. & Hitch, G. J. (1994). Developments in the concept of working memory. *Neuropsychology*, *8*(4), 485–493, doi:10.1037/0894-4105.8.4.485.

Bays, P. M., Catalao, R. F. G., & Husain, M. (2009). The precision of visual working memory is set by allocation of a shared resource. *Journal of Vision*, *9*(10), 7–7, doi:10.1167/9.10.7.

Bays, P. M. & Husain, M. (2008). Dynamic shifts of limited working memory resources in human vision. *Science*, *321*(5890), 851–854, doi:10.1126/science.1158023.

Benjamini, Y. & Hochberg, Y. (1995). Controlling the false discovery rate: A practical and powerful approach to multiple testing. *Journal of the Royal Statistical Society Series B: Statistical Methodology*, *57*(1), 289–300, doi:10.1111/j.2517-6161.1995.tb02031.x.

Bersier, N. M., Fornari, E., Rumiati, R. I., & Ionta, S. (2025). Cognitive traits shape the brain activity associated with mental rotation. *Cerebral Cortex*, *35*(4), doi:10.1093/cercor/bhaf069.

Cook, R. J., Dickens, B. M., & Fathalla, M. F. (2003). *World Medical Association Declaration of Helsinki: Ethical principles for medical research involving human subjects*, (pp. 428–432). Oxford University Press.

Cowan, N. (2001). The magical number 4 in short-term memory: A reconsideration of mental storage capacity. *Behavioral and Brain Sciences*, *24*(1), 87–114, doi:10.1017/s0140525x01003922.

Crump, M. J. C., McDonnell, J. V., & Gureckis, T. M. (2013). Evaluating amazon's mechanical turk as a tool for experimental behavioral research. *PLoS ONE*, *8*(3), e57410, doi:10.1371/journal.pone.0057410.

Ebert, W. M., Jost, L., Jansen, P., Stevanovski, B., & Voyer, D. (2025). Visual working memory as the substrate for mental rotation: A replication. *Psychonomic Bulletin & Review*, *32*(3), 1204–1216, doi:10.3758/s13423-024-02602-4.

Faul, F., Erdfelder, E., Lang, A.-G., & Buchner, A. (2007). G*power 3: A flexible statistical power analysis program for the social, behavioral, and biomedical sciences. *Behavior Research Methods*, *39*(2), 175–191, doi:10.3758/bf03193146.

Guthery, F. S., Burnham, K. P., & Anderson, D. R. (2003). Model selection and multimodel inference: A practical information-theoretic approach. *The Journal of Wildlife Management*, *67*(3), 655, doi:10.2307/3802723.

Hauser, D. J. & Schwarz, N. (2016). Attentive turkers: Mturk participants perform better on online attention checks than do subject pool participants. *Behavior Research Methods*, *48*(1), 400–407, doi:10.3758/s13428-015-0578-z.

AUTONOMOUSLY GENERATED

Hyun, J.-S. & Luck, S. J. (2007). Visual working memory as the substrate for mental rotation. *Psychonomic Bulletin & Review*, *14*(1), 154–158, doi:10.3758/bf03194043.

Lachenbruch, P. A. & Cohen, J. (1989). Statistical power analysis for the behavioral sciences (2nd ed.). *Journal of the American Statistical Association*, *84*(408), 1096, doi:10.2307/2290095.

Lange, N. & Fisher, N. I. (1995). Statistical analysis of circular data. *Journal of the American Statistical Association*, *90*(430), 801, doi:10.2307/2291098.

Li, X., Oestreich, L. K. L., Rangelov, D., Lévy-Bencheton, D., & O'Sullivan, M. J. (2024). Intrinsic functional networks for distinct sources of error in visual working memory. *Cerebral Cortex*, *34*(10), doi:10.1093/cercor/bhae401.

Luck, S. J. & Vogel, E. K. (1997). The capacity of visual working memory for features and conjunctions. *Nature*, *390*(6657), 279–281, doi:10.1038/36846.

Ma, W. J. (2018). Problematic usage of the zhang and luck mixture model.

Marks, D. F. (1973). Visual imagery differences in the recall of pictures. *British Journal of Psychology*, *64*(1), 17–24, doi:10.1111/j.2044-8295.1973.tb01322.x.

Marks, D. F. (1995). New directions for mental imagery research. *Journal of Mental Imagery*, *19*, 153–167.

Miyake, A., Friedman, N. P., Rettinger, D. A., Shah, P., & Hegarty, M. (2001). How are visuospatial working memory, executive functioning, and spatial abilities related? a latent-variable analysis. *Journal of Experimental Psychology: General*, *130*(4), 621–640, doi:10.1037/0096-3445.130.4.621.

Muller, K. (1989). Statistical power analysis for the behavioral sciences. *Technometrics*, *31*(4), 499–500, doi:10.2307/1270020.

Nosek, B. A., Ebersole, C. R., DeHaven, A. C., & Mellor, D. T. (2018). The preregistration revolution. *Proceedings of the National Academy of Sciences*, *115*(11), 2600–2606, doi:10.1073/pnas.1708274114.

Oberauer, K., Stoneking, C., Wabersich, D., & Lin, H.-Y. (2017). Hierarchical bayesian measurement models for continuous reproduction of visual features from working memory. *Journal of Vision*, *17*(5), 11, doi:10.1167/17.5.11.

Open Science Collaboration (2015). Estimating the reproducibility of psychological science. *Science*, *349*(6251), doi:10.1126/science.aac4716.

Palan, S. & Schitter, C. (2018). Prolific.ac—a subject pool for online experiments. *Journal of Behavioral and Experimental Finance*, *17*, 22–27, doi:10.1016/j.jbef.2017.12.004.

Peer, E., Rothschild, D., Gordon, A., Evernden, Z., & Damer, E. (2021). Data quality of platforms and panels for online behavioral research. *Behavior Research Methods*, *54*(4), 1643–1662, doi:10.3758/s13428-021-01694-3.

R Core Team (2014). R: A language and environment for statistical computing. *R Foundation for Statistical Computing*.

AUTONOMOUSLY GENERATED

Shah, P. & Miyake, A. (1996). The separability of working memory resources for spatial thinking and language processing: An individual differences approach. *Journal of Experimental Psychology: General*, *125*(1), 4–27, doi:10.1037/0096-3445.125.1.4.

Shepard, R. N. & Metzler, J. (1971). Mental rotation of three-dimensional objects. *Science*, *171*(3972), 701–703, doi:10.1126/science.171.3972.701.

Shepard, S. & Metzler, D. (1988). Mental rotation: Effects of dimensionality of objects and type of task. *Journal of Experimental Psychology: Human Perception and Performance*, *14*(1), 3–11, doi:10.1037/0096-1523.14.1.3.

van den Berg, R., Shin, H., Chou, W.-C., George, R., & Ma, W. J. (2012). Variability in encoding precision accounts for visual short-term memory limitations. *Proceedings of the National Academy of Sciences*, *109*(22), 8780–8785, doi:10.1073/pnas.1117465109.

Vandenberg, S. G. & Kuse, A. R. (1978). Mental rotations, a group test of three-dimensional spatial visualization. *Perceptual and Motor Skills*, *47*(2), 599–604, doi:10.2466/pms.1978.47.2.599.

Wilken, P. & Ma, W. J. (2004). A detection theory account of change detection. *Journal of Vision*, *4*(12), 11, doi:10.1167/4.12.11.

Zhang, W. & Luck, S. J. (2008). Discrete fixed-resolution representations in visual working memory. *Nature*, *453*(7192), 233–235, doi:10.1038/nature06860.

AUTONOMOUSLY GENERATED

5 Supplementary Material

5.1 Supplementary Results

5.1.1 Task Validation and Participant Engagement

Task validation procedures confirmed the experimental manipulations produced expected performance patterns while maintaining high participant engagement throughout the study protocol. Mental rotation accuracy demonstrated the anticipated systematic decline with increasing angular disparity, establishing the validity of the difficulty manipulation

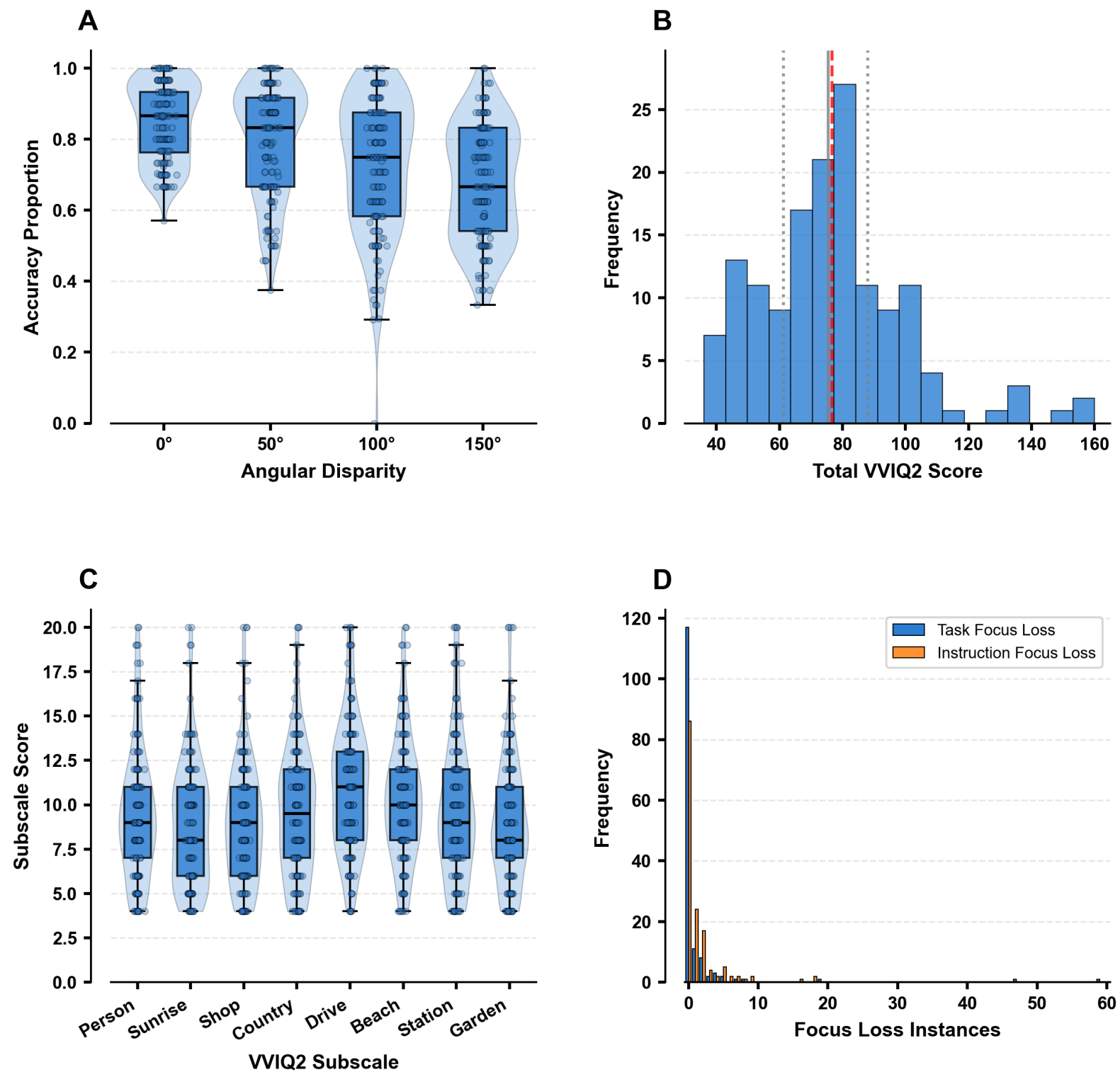

Figure 6: Task validation confirms expected difficulty gradients and high participant engagement across cognitive measures. Mental rotation accuracy demonstrates systematic decline with increasing angular disparity, validating the manipulation from near-ceiling performance at 0° (M=0.85) to chance-level at 150° (M=0.61). VVIQ2 scores span the full theoretical range with approximately normal distribution (M=76.6, range: 36-160), providing adequate variance for correlational analyses. Consistent subscale patterns across eight imagery scenarios support questionnaire reliability. Minimal focus loss instances (task M=1.32, instruction M=1.40) validate attention control and cognitive performance measures. (A) Mental rotation accuracy across angular disparities. Blue violin plots show probability density; box plots display quartiles with black median lines; individual points show participant scores with jittered positioning. (B) VVIQ2 total score histogram with blue bars. Vertical lines mark first quartile (grey dotted), median (grey solid), third quartile (grey dotted), and mean (red dashed). (C) VVIQ2 subscale scores across eight scenarios. Blue violin plots show distributions; box plots display quartiles; individual points are jittered. (D) Focus loss frequency histogram with blue bars (task) and orange bars (instruction). All panels: n=148 participants. Grid lines aid interpretation; transparency handles overlapping data points.

Performance ranged from near-ceiling accuracy at 0° rotation ($M = 0.85$) to chance-level performance at 150° rotation ($M = 0.61$), confirming that the angular disparity manipulation successfully

created graded difficulty levels across the four experimental conditions.

Visual imagery vividness scores obtained through the VVIQ2 questionnaire spanned the full theoretical range with an approximately normal distribution (M = 76.6, range: 36-160), providing adequate variance for subsequent correlational analyses examining individual differences in imagery ability. The distribution characteristics indicated that the sample captured substantial individual variation in self-reported visual imagery vividness, essential for detecting meaningful associations with objective cognitive performance measures. Consistent response patterns across the eight VVIQ2 subscales (familiar person, sunrise, shop front, countryside, driving, beach, railway station, and garden scenarios) supported the questionnaire's internal reliability, with all subscales showing similar distributional properties and central tendencies.

Attention control measures confirmed exceptional participant engagement throughout the experimental protocol. Focus loss instances remained minimal during both task performance (M = 1.32) and instruction reading (M = 1.40), validating the effectiveness of attention monitoring procedures and supporting the integrity of cognitive performance measures. These low rates of attentional lapses provided confidence that observed individual differences in task performance reflected genuine cognitive abilities rather than variable engagement or compliance across participants.

5.1.2 Mixture Model Diagnostics and Precision Estimation Robustness

Circular statistical modeling procedures demonstrated robust precision estimation with notable methodological sensitivity across different analytical approaches

AUTONOMOUSLY GENERATED

Table 3: Mixture model diagnostics reveal robust precision estimation with methodological sensitivity across circular statistical approaches. Three-component mixture models achieved 100% convergence across all participants (n=148), with von Mises distributions providing optimal fits for 71.6% of cases based on Akaike Information Criterion (AIC) model selection. Alternative precision measures showed substantial correlation (r=0.748, 95% CI: 0.667-0.811) between von Mises concentration parameters (κ) and simple precision estimates (1/circular standard deviation). Despite high inter-correlation, simple precision estimates demonstrated greater statistical sensitivity, detecting significant relationships in mental rotation task (MRT) analyses where von Mises κ parameters did not reach significance thresholds. Section 1 presents convergence rates and model selection frequencies with mean±SD statistics; Section 2 reports precision measure correlations with 95% confidence intervals; Section 3 compares standardized regression coefficients (β) and p-values between precision measures across MRT angular disparities (0°, 50°, 100°, 150°) and visual imagery (VVIQ-2) outcomes. All p-values reflect False Discovery Rate correction applied within each precision measure. Results demonstrate methodological robustness of precision-performance relationships while highlighting differential sensitivity of circular statistical approaches in detecting cognitive associations.

**Section 1: Mixture Model Convergence and Fit Statistics**		
**Statistic**	**Value**	**Percentage**
Model Convergence Rate	148/148	100.0%
Best Model: von_mises	106	71.6%
Best Model: wrapped_normal	22	14.9%
Best Model: wrapped_cauchy	20	13.5%
AIC Difference (Mean ± SD)	-0.90 ± 2.69	-
AIC Difference (Range)	-19.56 to 0.00	-
von Mises κ (Mean ± SD)	13.950 ± 6.973	-
Guess Rate (Mean ± SD)	0.021 ± 0.031	2.1%
**Section 2: Alternative Precision Measure Correlations**		
**Measure 1**	**Measure 2**	**Correlation [95% CI], N**
von Mises κ	Simple Precision	0.748 [0.667, 0.811], $N = 148$
**Section 3: Sensitivity Analysis Comparison**		
**Outcome**	**von Mises κ (β, p)**	**Simple Precision (β, p)**
MRT Accuracy (0°)	$0.445, p = 0.085$	$0.480, p = 0.068$
MRT RT (log) (0°)	$-0.045, p = 0.078$	$-0.020, p = 0.433$
MRT Accuracy (50°)	$0.401, p = 0.075$	$0.426, p = 0.063$
MRT RT (log) (50°)	$-0.021, p = 0.455$	$0.011, p = 0.691$
MRT Accuracy (100°)	$0.355, p = 0.073$	$0.413, p = 0.044$
MRT RT (log) (100°)	$0.002, p = 0.943$	$0.036, p = 0.218$
MRT Accuracy (150°)	$0.255, p = 0.165$	$0.292, p = 0.119$
MRT RT (log) (150°)	$0.014, p = 0.630$	$0.058, p = 0.044$
Total VVIQ-2 Score (Overall)	$1.203, p = 0.730$	$3.056, p = 0.273$

AUTONOMOUSLY GENERATED

Three-component mixture models achieved complete convergence across all 148 participants (100% convergence rate), indicating that the circular statistical framework successfully characterized individual error distributions without computational failures or parameter estimation difficulties.

Model selection procedures based on Akaike Information Criterion revealed that von Mises distributions provided optimal fits for the majority of participants (71.6%), with wrapped normal (14.9%) and wrapped Cauchy (13.5%) distributions representing best fits for smaller subsets of participants. The predominance of von Mises distributions supported the theoretical assumption

that visual working memory errors follow von Mises distributions centered on target orientations, while the presence of alternative best-fitting distributions across participants highlighted meaningful individual differences in error distribution shapes.

Alternative precision measures demonstrated substantial intercorrelation while revealing differential statistical sensitivity in detecting cognitive associations. Von Mises concentration parameters (κ) and simple precision estimates (1/circular standard deviation) showed strong correlation (r = 0.748, 95% CI: 0.667-0.811), confirming that both approaches captured similar underlying precision constructs. However, comparative analyses revealed that simple precision estimates demonstrated greater sensitivity in detecting statistically significant relationships with mental rotation task performance, identifying significant associations at multiple angular disparities where von Mises κ parameters failed to reach significance thresholds after False Discovery Rate correction.

This methodological sensitivity differential highlighted the importance of precision estimation approach selection in studies examining individual differences in visual working memory. While both measures reflected similar precision constructs at the population level, the enhanced statistical power of simple precision estimates suggested potential advantages for detecting subtle cognitive associations in correlational research designs. The robust convergence rates and consistent model selection patterns across participants provided confidence in the stability of precision estimation procedures, supporting the validity of subsequent analyses examining relationships between visual working memory precision and visuospatial cognitive abilities.

AUTONOMOUSLY GENERATED
